# Fast Solution-Phase and Liquid-Phase Peptide Syntheses (SolPSS and LPPS) Mediated by Biomimetic Cyclic Propylphosphonic Anhydride (T3P^®^)

**DOI:** 10.3390/molecules28207183

**Published:** 2023-10-19

**Authors:** Alexia Mattellone, Dario Corbisiero, Paolo Cantelmi, Giulia Martelli, Chiara Palladino, Alessandra Tolomelli, Walter Cabri, Lucia Ferrazzano

**Affiliations:** 1 Tolomelli-Cabri/P4I Lab—Peptidomimetics and Peptides Targeting Protein-Protein Interaction, Department of Chemistry “Giacomo Ciamician”, Alma Mater Studiorum—University of Bologna, Via Gobetti 87, 40129 Bologna, Italy; alexia.mattellone2@unibo.it (A.M.); dario.corbisiero2@unibo.it (D.C.); pa.cantelmi@gmail.com (P.C.); giulia.martelli8@unibo.it (G.M.); chiara.palladino5@unibo.it (C.P.); lucia.ferrazzano4@unibo.it (L.F.); 2 Consorzio C.I.N.M.P.I.S. (National Interuniversity Research Consortium in Innovative Synthesis Methodologies and Processes) c/o, Alma Mater Studiorum—University of Bologna, Via Gobetti 87, 40129 Bologna, Italy

**Keywords:** biomimetic reaction, peptide synthesis, homogeneous reaction, SolPPS, LPPS, T3P^®^

## Abstract

The growing applications of peptide-based therapeutics require the development of efficient protocols from the perspective of an industrial scale-up. T3P^®^ (cyclic propylphosphonic anhydride) promotes amidation in the solution-phase through a biomimetic approach, similar to the activation of carboxylic moiety catalyzed by ATP-grasp enzymes in metabolic pathways. The T3P^®^ induced coupling reaction was applied in this study to the solution-phase peptide synthesis (SolPPS). Peptide bond formation occurred in a few minutes with high efficiency and no epimerization, generating water-soluble by-products, both using *N*-Boc or *N*-Fmoc amino acids. The optimized protocol, which was successfully applied to the iterative synthesis of a pentapeptide, also allowed for a decrease in the solvent volume, thus improving process sustainability. The protocol was finally extended to the liquid-phase peptide synthesis (LPPS), where the isolation of the peptide was performed using precipitation, thus also showing the suitability of this coupling reagent to this emerging technique.

## 1. Introduction

The importance of peptide-based therapeutics is nowadays recognized and witnessed through the number of molecules launched on the market (>80), in clinical development (>150), and in the preclinical phase (400–600) [1,2]. The increasing potential of this class of compounds, which allows for forecasting a market size of >USD 50 billion in 2026 [3], is related to their established high clinical development success rate and likelihood of approval (LoA), when compared to small molecules [4]. Despite the growing potential in therapeutic applications, native peptides often display an unfavorable pharmaceutical profile and, to overcome limitations such as short half-lives and poor oral bioavailability, structural modifications like the introduction of unnatural amino acids, cyclization, synthesis of stapled peptides and cyclotides, and conjugation with stabilizing lipids or pegylated chains have been applied [5]. The presence of unusual motifs does not allow one to obtain these sequences by recombinant techniques, and for this reason, the development of stable and sustainable approaches to peptide synthesis and purification is needed to deliver pharmaceutical-grade peptides and accomplish regulatory and environmental health and safety (EHS) requirements. In this context, optimizing all the components of peptide synthesis and purification by applying, when possible, a sustainable approach has been explored in the last few years by several groups [6,7,8] by taking into consideration, one at a time, all the actors playing a role in the upstream and downstream productive phases [9,10].

Although the solid-phase peptide synthesis (SPPS) is nowadays the most applied and reliable technology, the use of large amounts of reagents and large volumes of solvents represents the most critical issue for an industrial upscale. Recently, renewed attention has been paid to the solution-phase peptide synthesis (SolPPS), which allows for the use of stoichiometric amounts of reagents but still requires large volumes of solvents during the unavoidable isolation and purification of each intermediate. The introduction of anchors enables researchers to exploit the solubility of the growing peptide, perform reactions in a homogeneous phase, and remove side products after every coupling/deprotection sequence via aqueous washings or through the precipitation of the intermediate peptides. This technology has opened the so called “third wave” of peptide synthesis, the liquid-phase approach (LPPS) [11]. It takes advantage of the combination of the Fmoc iterative protocol with a “tagged” *C*-terminal residue, usually bearing a benzyl ester decorated with long lipophilic chains.

The selection of a proper coupling reagent plays a crucial role in the design of a green, safe, and efficient protocol, mainly in the aspects of large-scale chemical processes. The development of sustainable methods for the formation of amide bonds has indeed been included as one of the 10 key green chemistry research areas by the ACS Green Chemistry Institute Pharmaceutical Roundtable (GCIPR) [12].

Amide bond formation is carried out using a diverse array of activating reagents and additives, as different peptides may require specific conditions for an optimal synthesis. And, to date, there is still no universally preferred reagent. Combinations of the available reagents on the market and their efficacy under different conditions have been extensively investigated, but very few examples can be described as green approaches [13]. Defining general sustainability criteria for coupling reagents in peptide synthesis remains challenging. Researchers consider factors like environmental impact, safety, and toxicity that need to match the cost-effectiveness, efficiency, and reproducibility of the amide formation. Achieving a balance among these aspects is crucial for promoting more sustainable and efficient peptide synthesis processes. Efforts are ongoing to establish comprehensive guidelines for greener and more sustainable practices. [14], but classification criteria were exclusively based on the thermal stability of the reagents, and for this reason, only the process safety point of view was taken into consideration. Other hazardous features or disadvantages have emerged in recent decades, thus limiting the applicability of the most popular coupling reagents in many cases.

Since the 1960s, peptide bond formation in SPPS has been performed by activating the carboxylic moiety with a carbodiimide in the presence of an additive, which was initially with the use of dicyclohexylcarbodiimide (DCC) that was soon replaced by diisopropylcarbodiimide (DIC) due to the higher solubility of the urea side-product [15,16]. As for the additives, benzotriazoles (HOBt, HOAt, HBTU) have been widely applied, but the risk of explosion and the skin sensitization due to long-term exposure represent major concerns [17]. Despite new members of this family displaying enhanced stability, the development of industrial processes using benzotriazoles has been stopped by big pharma to shift toward safer alternatives. In this context, Albericio and the coworkers in 2009 introduced a new class of oxymes, reporting for the first time the reactivity of OxymaPure^®^ [18], an additive displaying excellent properties regarding yields, low racemization levels, and increased safety compared to benzotriazole-based reagents. Although many other analogue derivatives have been identified in the following years, for instance K-Oxyma, PyOxyma, Oxyma B and Oxyma T, the OxymaPure^®^ is still the first choice for manual, automated, and MW-assisted SPPS protocols. Among the number of possible additives, the combination with DIC is the benchmark in peptide synthesis due to the excellent safety profile, the relative cheapness, and the good solubility of the diisopropylurea (DIU) by-product in many organic green solvents. Recently, concerns related to the possible formation of HCN during amino acid activation with this protocol have been raised [19], and deeper investigations of the side reaction have allowed for developing methods to minimize this issue. Moreover, Albericio’s group evaluated alternatives, identifying N-tert-butyl-N′-ethylcarbodiimide (TBEC) as a new efficient additive able to avoid HCN formation [20].

In the field of LPPS, much attention has been paid to the structure and the physicochemical properties of the anchored tag, which is crucial for the isolation and purification of the growing peptide, but the choice of the coupling reagents has often been inspired by Fmoc-SPPS technology, and the combination of benzotriazole tetramethyluronium derivatives (HBTU/HATU) with hydroxybenzotriazoles or carbodiimides (EDC, DIC) with a base have been commonly used [11].

We recently became interested in a biomimetic approach by using T3P^®^ (cyclic Propylphosphonic anhydride), which was able to promote amidation in the solution-phase with a high efficiency and no epimerization, generating water-soluble by-products [21,22].

The formation of a phosphoric anhydride to activate a carboxylic moiety is a mechanism selected by nature in millions of years to overcome the thermodynamical barriers due to unfavorable leaving groups. In diverse primary and secondary metabolic processes, the amide bond formation is indeed catalyzed by enzymes belonging to the ATP-grasp family [23]. These enzymes share a common mechanism involving two half-reactions, wherein a carboxylic acid substrate is first activated as an acylphosphate intermediate prior to condensation with a co-substrate nucleophile. This mechanism allows for an efficient and specific amide bond formation in biological processes. A remarkable feature of the ATP-grasp family is the structurally diverse range of substrates that are utilized by each enzyme, since different members of the family can accept simple carboxylic acid substrates while others bind to large proteins serving as substrates. In these metabolic pathways, the enzyme lowers the activation energy to zero, causing the first step of the reaction to be spontaneous. The mixed anhydride thus obtained is extremely reactive in the presence of nucleophiles. Organophosphates, as acetyl phosphate, have also been suggested as acylating agents in protein-modifying reactions [24].

**Figure 1:**
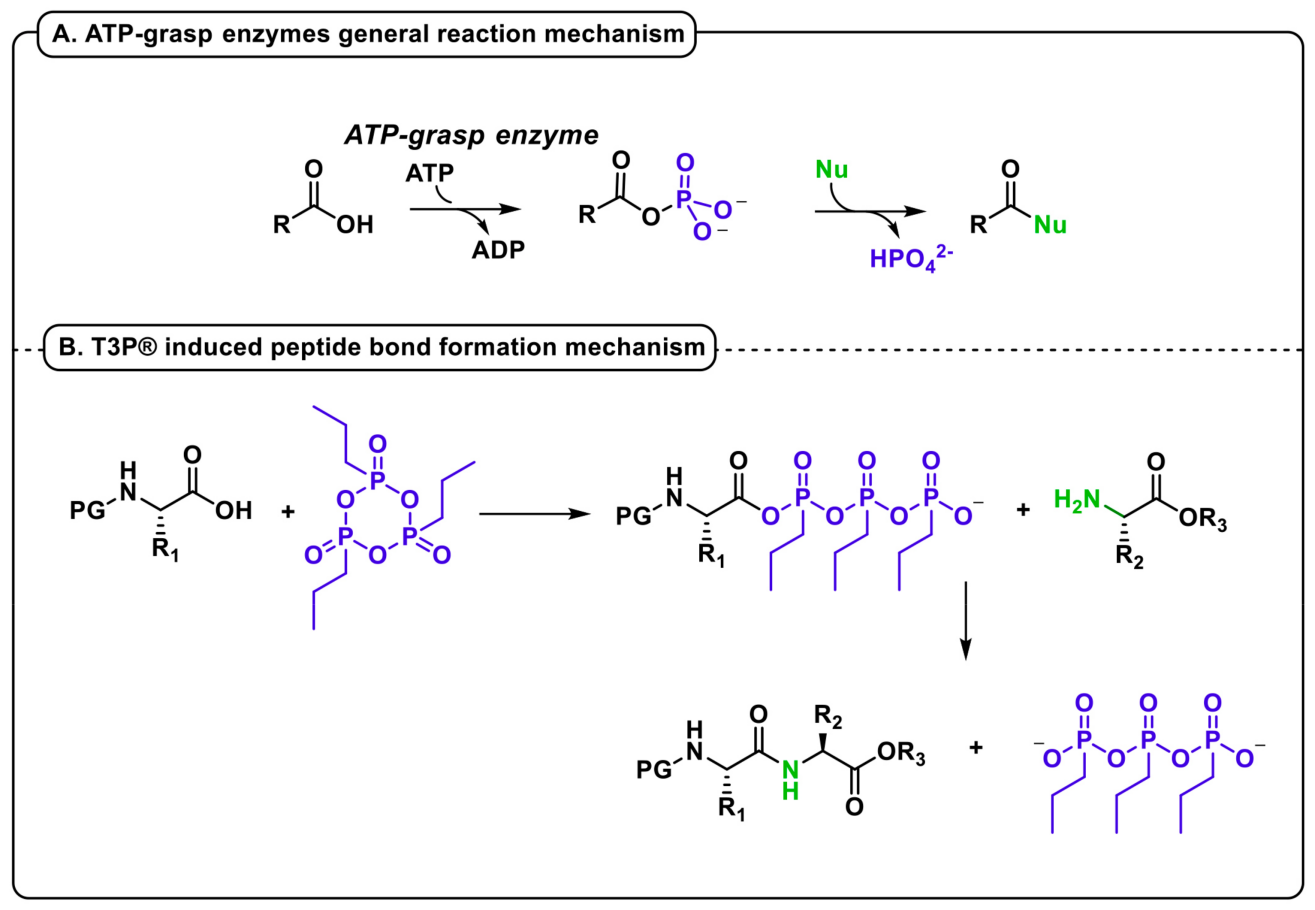
(**A**) General reaction mechanism of ATP-grasp enzymes (**B**) T3P^®^ induced peptide bond formation mechanism.

In the case of peptide synthesis with T3P^®^, its cyclic structure leads to a spontaneous reaction with the nucleophilic carboxylate of the *N-*protected amino acid, followed by a fast substitution with the amino group of a second residue (Figure 1B). The extreme reactivity of the acylphosphate intermediate with every nucleophile, including water, is the reason for the common application of T3P^®^ as a moisture scavenger [25]. Anyway, this behavior unfortunately represents a great limitation when this reagent is applied to iterative processes requiring intermediate treatments with water solutions. Indeed, although in the past T3P^®^ was claimed several times as a peptide coupling reagent, its use was only reported for the formation of a single amide bond; the first example of an iterative synthesis in SolPPS, without the isolation of intermediate oligomers, has been recently reported by our group [26]. The use of T3P^®^ in peptide syntheses, therefore, considers two main issues: the steric hinderance of the reagent itself and the extreme sensitivity to water traces. The application as a coupling reagent in SPPS did not afford the expected results because under these conditions, T3P^®^ worked only as an alternative to carbodiimides, and the reaction required large excesses of OxymaPure^®^ and diisopropyl ethyl amine (DIPEA) [27]. This result may be related to a difficult approach of the bulky reagent to the growing peptide anchored on the resin. On the contrary, in SolPPS, the main issue lies in the dryness of the solution during the iterative process. By avoiding aqueous work-up using benzylcarbamate as an amine protecting group and Pd-catalyzed hydrogenation for its removal, we developed a one-pot, sustainable, fast, and efficient methodology for a short peptide synthesis, using T3P^®^ as the only coupling reagent in the presence of a base [26].

The greatest advantage of this methodology was the speed of the coupling reaction, which achieved completeness in a few minutes. Therefore, due to the renewed interest in the homogeneous-phase peptide synthesis, we decided to investigate herein the extension of our biomimetic approach to in SolPPS and LPPS using *N*-Boc- and *N*-Fmoc-based protecting schemes, respectively.

## 2. Results

In our previous study, through an exhaustive investigation, we identified the best reaction conditions for the SolPPS of short peptides using *N*-Z-protected amino acids for sequence elongation and O*^t^*Bu-protected amino esters as the starting units, observing a complete formation of the amide bond after 5 min by using a 1.5/3 ratio of T3P^®^ and DIPEA as a coupling agent at room temperature. For this reason, we decided to apply these conditions in the same reaction using different protecting schemes, as described in detail in the following paragraphs.

- **Solvent suitability**

**Scheme 1:**
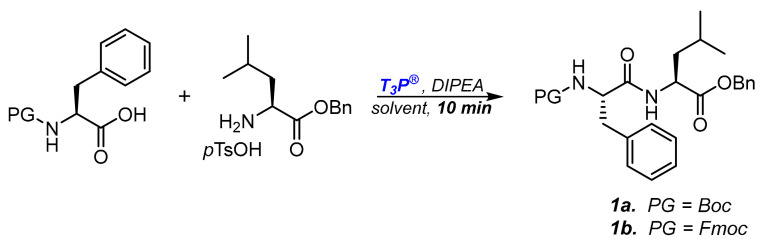
Model reaction for T3P^®^ induced peptide synthesis.

Moving from *N*-Z-protection to *N*-Boc- or *N*-Fmoc ones, we initially explored the efficiency of several solvents in the coupling step. In fact, together with the necessity to ensure complete and fast conversions during the couplings mediated by T3P^®^, we had to consider the reagent’s sensitivity to water: aqueous work-up is necessary to remove coproducts of the coupling but could affect the efficiency of the coupling reagent introduced later in the synthesis, even if in traces. To perform the screening, we chose as the standard coupling the reaction between *N*-protected-phenylalanine and leucine benzyl ester (Scheme 1), measuring the conversion to dipeptide **1a**,**b** after ten minutes. The reactions were performed under a nitrogen atmosphere in dry solvents. Since the reacting amino ester was commercially available as salified benzyl ester *p*-toluenesulfonate salt, one equivalent of DIPEA was always added to activate the nucleophilic amine moiety. The reaction was quenched after ten minutes by adding water and removing the phosphate coproducts with an organic solvent/water extraction. The results obtained are reported in Table 1.

In SPPS, the use of DMF is still considered convenient for the exceptional ability of this solvent to dissolve all the possible reagents in coupling reactions regardless of its toxicity. On the contrary, its application in solution is less recommended because of the issue related to its miscibility with water during work-up and its high boiling point. In our study, the standard reaction was initially run in DMF as a benchmark to compare the performance of the other organic solvents (entries 1, 2). To our satisfaction, conversions > 95% were observed after 10 min by using almost all selected solvents. Worse results were obtained only when the reaction of *N*-Boc-phenylalanine was performed in cyclopentylmetylether (CPME), tertbutyl acetate (*^t^*BuOAc), and *N*-Octyl pyrrolidone (NOP) due to solubility issues that probably affected the reaction speed. The solubility issue was overcome by performing the reaction with *N*-Fmoc-phenylalanine, which enabled an almost-complete conversion in the critical solvents (entries 8, 14, 22), being perfectly dissolved in the reaction medium.

T3P^®^ has been particularly studied for its valuable effect on preventing racemization in peptide bond formation, and to verify this behavior, the degree of racemization for the reaction between *N*-Boc-(L)-phenylglycine and leucine methyl ester was evaluated by investigating the possible presence of the (D)-phenylglycine containing dipeptide. The detected amount of racemized product was around 0.5% (see Supplementary Materials for details).

In view of an iterative oligopeptide synthesis, the presence of possible residual moisture traces derived from aqueous work-up has to be avoided, and the solubility of water in the reaction medium represents a critical parameter. By performing the coupling reaction using non-anhydrous solvents, we always observed a significant drop in yield, except in the case of dichloromethane (DCM), which resulted in yields > 95% in any condition because of the low solubility of water in this solvent (below 0.24%) [28].

- **Substrate scope**

The substrate scope extension was then performed, also taking into consideration the requirements for the further design of oligomers synthesis. The reactivity of *N*-Boc-amino acids was tested in the reaction with leucine, selecting methyl ester as the orthogonal protection in the final dipeptide. Moreover, since the *N*-protecting group removal had to be performed with trifluoroacetic acid (TFA) and washing the crude with water to remove salts is needed after this step, DCM was selected as the solvent of choice for the above reported low tendency to retain moisture traces. On the other hand, the *N*-Fmoc-protecting group is not suitable for SolPPS but is commonly used in LPPS, where the nucleophilic amino acid is functionalized as a modified benzylic ester. For this reason, the scope of *N*-Fmoc-amino acids reactivity was tested in the reaction with leucine benzyl ester. Selected results are reported in Table 2.

To verify if the exceptional speed of the T3P^®^ promoted coupling reaction was maintained regardless of the activated amino acid, we evaluated the conversion with all substrates after 10 min. Regarding the protection couple *N*-Boc/*C*-OMe, the coupling of *N*-Boc-Leu and *N*-Boc-Phe was performed at first affording excellent conversions (entries 1 and 2, Table 2). Moving to amino acids bearing a protected functionality in the side chain, as *N*-Boc-Arg(Pbf)-OH and *N*-Boc-Asp(Bzl)-OH, satisfactory results were observed as well, being the conversion almost complete with protected arginine (99%) and very good with the aspartic acid derivative (96%) (entries 3 and 4, Table 2). On the basis of these promising results, the coupling reaction was then attempted on *N*-Boc amino acids bearing a free functional group in the side chain. The resulting optimized conditions were also suitable to *N*-Boc-Ser-OH and *N*-Boc-Thr-OH, bearing a free hydroxyl group in the side chain (entries 5 and 6, Table 2). Moreover, the reaction of *N*-Boc-Trp-OH with leucine methyl ester afforded a 96% yield (entry 7, Table 2), thus suggesting that the coupling is kinetically faster than any other side reaction involving side chain functionalities. Finally, a complete conversion was also observed in the reaction of *N*-Boc-Aib-OH, an amino acid usually displaying a challenge related to the steric hindrance of the quaternary center (entry 8, Table 2).

In a similar way, a selection of *N*-Fmoc amino acids was submitted to the coupling reaction with H-Leu-ObBn, affording results comparable to those obtained with *N*-Boc monomers. In this case, commercially available *N*-Fmoc-Ser(*^t^*Bu)-OH and *N*-Fmoc-Thr(*^t^*Bu)-OH were used in the screening, enabling excellent conversions (entries 13 and 14, Table 2) always. It is worth noting that *N*-Fmoc-Arg-OH, bearing the free guanidine moiety as a salt with hydrochloric acid, enabled > 99% conversion (entry 15, Table 2), thus opening the possibility to avoid the problematic protection of the side chain with Pbf. Instead, the protocol was not suitable for the coupling involving *N*-Boc-Cys-OH since the reaction afforded a mixture of the dipeptide and oxidized derivatives of cysteine. On the other hand, the activation of *N*-Boc-Lys-OH with T3P^®^ exclusively enabled the cyclic product derived from the intramolecular attack of the amine of the side chain to the activated carboxylate.

- **SolPPS and LPPS of Leu-enkephalin with T3P^®^**

Based on the previous information, the protocol was applied to the synthesis of Leu-Enkephalin (H-Tyr-Gly-Gly-Phe-Leu-OH), a pentapeptide that has been commonly prepared to test the efficacy of a methodology in comparison with other synthetic techniques [29].

**Scheme 2:**
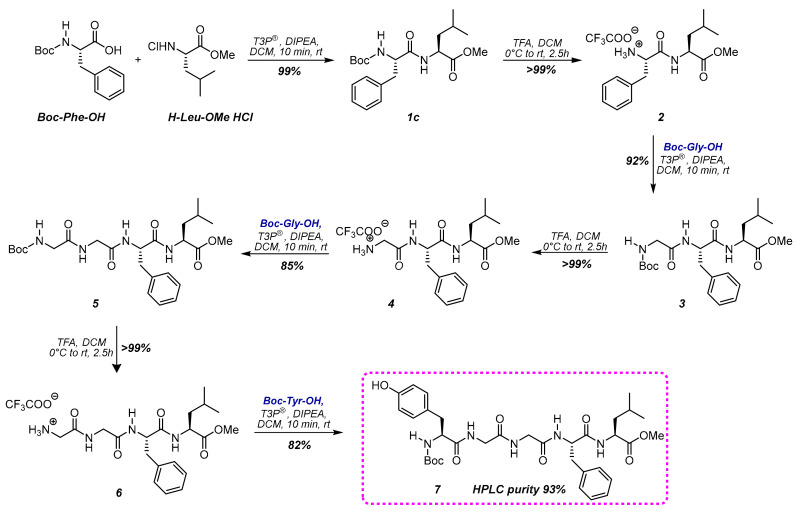
SolPPS of *N*-Boc-Leu-Enkephalin-OMe **7** (reported yields refer to crude reactions).

Firstly, we performed the SolPPS with *N*-Boc protected monomers in DCM (Scheme 2). The conditions applied for the coupling were the same used for the dipeptide formation reported in Table 1. The conversion of the coupling step was monitored using HPLC-MS, and reactions were always completed after ten minutes (HPLC data of each step are reported in the Supplementary Materials). The reaction mixture was then washed with hydrochloric acid (0.1 M) to remove possible residues of unreacted monomers and phosphate salts. The complete removal of the Boc group was induced through treatment with an excess of trifluoroacetic acid (TFA) in DCM from 0 °C to room temperature for 2.5 h. The solvent was then removed under vacuum to allow for isolation and analysis of each intermediate. This protocol produced *N*-Boc-Leu-enkephalin-OMe **7a** with a purity 93%. The overall yield of the seven steps before purification was 62%.

Peptide **7** was also prepared following the same reaction sequence, avoiding solvent evaporation to isolate intermediates, and submitting the initial DCM solution to all the iterative sequences of steps. The amount of T3P^®^ was slightly increased (2/4 ratio T3P^®^/DIPEA in respect to amino acids) to use it both as a scavenger for water traces and as an activating reagent in the coupling. Under these conditions, the purity of the final peptide was 95% (yield 62%), an excellent value considering that the final protected peptide was isolated only by solvent evaporation and not purified chromatographically.

Although the coupling conditions induced by T3P^®^ were completely compatible with *N*-Fmoc amino acids, the application of this protecting scheme for iterative SolPPS was limited by the need to remove the dibenzofulvene coproduct, which is not water soluble, before performing the following coupling step. For this reason, the T3P^®^ coupling protocol was applied to Fmoc-chemistry by using the LPPS approach, where an isolation of the peptide is performed through precipitation to keep nasty coproducts in the mother liquors. As a proof of concept to test our method, we applied the standard conditions to the Molecular Hiving technology developed by Jitsubo and Bachem [30,31] by selecting a 2,5-polycarbon substituted Hydrophobic Benzyl Alcohol (HBA) tag [32]. This specific anchor was designed for LPPS to achieve acidic colorimetric, photo-oxidative, and fluorometric properties that are useful for an easy quantitative assay of peptide synthesis reactions.

**Scheme 3:**
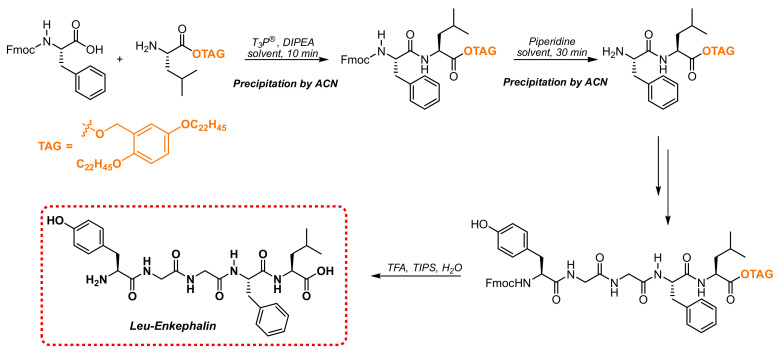
LPPS of Leu-Enkephalin using polycarbon tag.

The selected anchor, shown in Scheme 3, linked as an ester function to the carboxylic terminal leucine residue, was synthesized according to a method reported in the literature [31].

Building the oligopeptide involved a sequence of coupling reactions between *N*-Fmoc-amino acids and the growing peptide anchored on the selected lipophilic tag, which was under the previously optimized conditions and alternated to the removal of the *N*-Fmoc-protecting group with piperidine (Scheme 3). After each step, the isolation of the product could be performed through the addition of acetonitrile as a precipitating agent, followed by the filtration of the anchored growing chain and further washing with the same solvent. After the completion of the sequence, the simultaneous removal of the protecting groups and cleavage from the tag were obtained through treatment with a cocktail containing 95% TFA: 2.5% TIS: 2.5% H_2_O.

The LPPS protocol was initially performed in DCM using two methods: (i) isolating each intermediate through precipitation; (ii) inducing peptide isolation only after piperidine-mediated deprotection. Moreover, since in the LPPS approach the growing peptide is recovered through the filtration of the precipitate without any water treatment, the use of DCM could be avoided by switching to a greener solvent such as anisole.

In Table 3, a comparison of the results obtained with both solvents is reported. When the sequence was built in DCM, the final pentapeptide was obtained with purity > 99% both by isolating the product of each step and by performing precipitation only after the deprotection step. By performing the synthesis in anisole, slightly lower but excellent purity grades (95.9% and 91.4%) were obtained, with only small amounts of the shorter peptides derived from Tyr misincorporation being detectable in the last coupling step.

## 3. Discussion

The biomimetic approach to the formation of amide bonds in the solution-phase, which takes advantage of the extreme reactivity of the T3P^®^ coupling reagent, has been previously reported only regarding the formation of a single peptide bond. The extreme sensitivity of this cyclic phosphonic anhydride to water is indeed a problem when water traces may be released during work-up. For this reason, in a previous investigation, we matched this coupling reagent with *N*-Z protected amino acids, optimizing conditions that allowed for the removal of the protecting group by hydrogenation, thus avoiding aqueous work-up. In this investigation, the compatibility with *N*-Boc and *N*-Fmoc protecting groups and the suitability of several solvents have been established by expanding the scope of the coupling to a small library of amino acids, including monomers bearing protection to the side chain functionalities and monomers with free active moieties in the backbone. The excellent results prompted the application of this coupling reagent in the synthesis of a model pentapeptide via iterative SolPPS and LPPS.

In the first approach, DCM was the best-performing solvent, and *N*-Boc Leu-enkephalin methyl ester **7** was isolated in excellent yield and purity. Even if DCM is not a green solvent, the possibility to avoid solvent evaporation in each step while retaining the original solution during all the iterative sequences represents a great advantage for the decrease in the solvent volume, thus improving process sustainability. 

On the other hand, the LPPS protocol produced the free Leu-enkephalin with a purity grade comparable or even higher than those reported in the literature, both performing the iterative peptide synthesis in DCM or using the more sustainable anisole. The decrease in purity in the latter processes suggests that a trade-off between the purity of the peptide and the greenness of the solvent has to be accepted in this case when moving from DCM.

## 4. Conclusions

When choosing the coupling reagent, T3P^®^ has not commonly been considered useful in SPPS because its behavior is not comparable with the better-performing oxime reagents. 

Meanwhile, because of the renewed interest in SolPSS and LPPS, this safe and biomimetic coupling reagent may return to the limelight, and it deserves to be reconsidered not only for a single amide bond formation but also for iterative protocols. In particular, procedures suitable for its use in the solution of this biomimetic coupling reagent for *N*-Boc and *N*-Fmoc peptides have been identified, enabling a new tool for the production of peptides in view of the great success of this modality in the pharmaceutical and cosmetic segments. 

## 5. Materials and Methods

### 5.1. Methods

Unless otherwise stated, all materials, solvents, and reagents were obtained from commercial suppliers and used without further purification. High-performance liquid chromatography (HPLC) reagent-grade solvents were used. Specifically, *N-*fluorenylmethyloxycarbony (Fmoc), *N-*tert-butyloxycarbonyl (Boc) amino acids, and diisopropylethylenamine (DIPEA) were supplied by Iris Biotech, Merck, or Fluorochem. Ethyl Acetate (EtOAc), *N,N*-dimethylformamide (DMF), anisole, cyclopentyl methyl ether (CPME), dimethyl carbonate (DMC), *N*-octyl pyrrolidone (NOP), acetonitrile (ACN), tetrahydrofuran (THF), dichloromethane (DCM), propyl acetate (PrOAc), tertbutyl acetate (tBuOAc), and HPLC-quality acetonitrile (ACN), hexane, and propan-2-ol (iPrOH) were purchased from Merck. Trifluoroacetic acid (TFA), triisopropyl silane (TIPS), and diisopropyl ether (DIPE) were supplied by Iris Biotech and Merck. All other chemicals were purchased from Merck and Fluorochem. Solvents and coupling reagents were individually injected into HPLC using the same analysis methods employed for the evaluation of reactions progress to establish their retention time (Chapter S7 in the Supporting Information). T3P^®^ (50 wt. % in DCM) was supplied by Curia Global. 

### 5.2. Analytical Methods

Thin-layer chromatography (TLC) was performed on precoated silica gel 60 F254 plates (Merck, Darmstadt, Germany), and spot detection was carried out using UV light and/or by charring with a ninhydrin solution or permanganate solution. HPLC-MS analyses were performed on an Agilent 1260 Infinity II system coupled to an ESI mass spectrometer (positive-ion mode, *m*/*z* = 100–3000 amu, fragmentor 30 V) with the following parameters: column Phenomenex Luna C18 5 μm, 250 × 4.6 mm; temperature: 35 °C; injection volume: 10 µL; UV: 220 nm; H_2_O + 0.08%TFA (mobile phase A) and CAN + 0.08%TFA (mobile phase B). ChemStation software (OpenLAB CDS ChemStation 35900 A/D driver, OpenLAB CDS ChemStation 490 Micro GC driver) was used for data processing. Percentage areas of integrated peaks are reported in mAu. The racemization analyses were performed using HPLC employing Daicel Chiralpack IC column. ^1^H-NMR spectra were recorded with an INOVA 400 MHz instrument with a 5 mm probe and Bruker Avance 600 MHz. ^13^C-NMR spectra were recorded at 101 MHz with an INOVA 400 MHz instrument and at 151 MHz with the Bruker Avance 600 MHz instrument. All chemical shifts were quoted relative to deuterated solvent signals. Chemical shifts (δ) were referenced to the corresponding solvent peaks and are reported in parts per million (ppm). Coupling constants (J) are given in Hertz. Multiplicities are abbreviated as: s (singlet), d (doublet), t (triplet), q (quartet), m (multiplet), or combinations thereof.

### 5.3. Synthetic Procedures

#### 5.3.1. General Procedure of Coupling Step for the Solvent Screening and Substrate Scope

In an oven-dried Schlenk purged under a N_2_ atmosphere, *N-*Fmoc or *N-*Boc amino acid (0.125 mmol, 1.0 eq.) and H_2_N-Leu-OMe or H_2_N-Leu-OBn (0.125 mmol, 1.0 eq.) were dissolved in the desired solvent (1 mL, 0.125 M). DIPEA (0.5 mmol, 4 eq.) and T3P^®^ (92 µL, 50 wt. % in DCM; 0.188 mmol, 1.5 eq.) were then added at room temperature following this order. The solution was stirred at room temperature for 10 min, and the conversion was monitored by sampling the crude mixture and analysing it through HPLC-MS (method A or C in Chapter 1 of the supporting information). Chromatograms for all reactions are reported in Figures S1–S39. 

#### 5.3.2. Full SolPPS of Leu-Enkephalin via Boc chemistry

##### Protocol with Intermediate Isolation

An oven-dried, double-neck, round-bottomed flask equipped with a stirring bar was charged with *N*-Boc-Phe-OH (53 mg, 0.2 mmol, 1 eq.) and H-Leu-OMe hydrochloride (36 mg, 0.2 mmol, 1 eq.) in DCM (0.125M) under a N_2_ atmosphere. Subsequently, DIPEA (139 µL, 0.4 mmol, 4 eq.) and T3P^®^ (50 wt. % in DCM, 146 µL, 0.3 mmol, 1.5 eq.) were added following this order. The solution was stirred for 10 min, and the organic phase was washed once with H_2_O, HCl_(aq)_ (0.01 M). The organic layer was dried over anhydrous Na_2_SO_4_ and concentrated under vacuum, allowing for a white solid (77 mg, 0.196 mmol) in >98% yield. The crude product *N*-Boc-Phe-Leu-OMe was then dissolved with DCM (0.125 M), and TFA (273 µL, 3.6 mmol, 18 eq.) was added. The solution was stirred for 2.5 h, and the organic solvent was concentrated under vacuum, allowing for a white solid (57 mg, 0.196 mmol) in >99% yield. The same procedure mentioned above was used in subsequent steps, introducing: *N*-Boc-Gly-OH (1 eq.) and *N*-Boc-Tyr-OH (1 eq.). Each step was monitored through an HPLC-MS analysis (method A in the “HPLC methods” chapter in the supporting information). *N*-Boc-Tyr-Gly-Gly-Phe-Leu-OMe **7** was obtained as a white solid in 62% overall yield with 93% purity. Chromatograms for all reactions are reported in Figures S43–S49.

##### Continuous Protocol without Intermediate Isolation

An oven-dried, double-neck, round-bottomed flask equipped with a stirring bar was charged with *N*-Boc-Phe-OH (133 mg, 0.5 mmol, 1 eq.) and H-Leu-OMe hydrochloride (91 mg, 0.5 mmol, 1 eq.) in DCM (0.125M) under a N_2_ atmosphere. Subsequently, DIPEA (353 µL, 2 mmol, 4 eq.) and T3P^®^ (50 wt. % in DCM, 365 µL, 0.75 mmol, 1.5 eq.) were added following this order. The solution was stirred for 10 min, and the organic phase was washed once with H_2_O, HCl_(aq)_ (0.01 M) and NaHCO_3(aq)_ (0.01 M). The organic layer was dried over anhydrous Na_2_SO_4_ and was directly used in the next step, adding TFA (690 µL, 9 mmol, 18 eq.). The solution was stirred for 2.5 h and then the organic solvent was washed twice with sat. NaHCO_3(aq)_. The resulting solution was dried over anhydrous Na_2_SO_4_ and directly used in further steps following the procedure mentioned above, increasing the equivalents of DIPEA and T3P^®^ to 4 and 2 eq., respectively. *N*-Boc-Tyr-Gly-Gly-Phe-Leu-OMe **7** was obtained as a white solid in 62% overall yield with 95% purity. Each step was monitored by HPLC-MS analysis (method A in the chapter of “HPLC methods” in the supporting information). Chromatograms for all reactions are reported in Figures S50–S56.

#### 5.3.3. General Procedure for the Synthesis of 2,5-Polycarbon Substituted Hydrophobic Benzyl Alcohols (HBA) Tag

An oven-dried, double-neck, 50 mL round-bottomed flask equipped with a stirring bar, 2,5-dihydroxybenzaldehyde (0.5 g, 3.6 mmol, 1 eq.), and potassium carbonate (3.9 g, 28.2 mmol, 7.8 eq.) was suspended in dry DMF (0.16 M), and the mixture was stirred at 110 °C. Subsequently, 1-bromodocosane (4.2 g, 10.9 mmol, 3 eq.) was added dropwise, and the mixture was stirred at the same temperature for 3 h, followed by dilution with water (22 mL). The aqueous layer was extracted with toluene (30 mL × 3). The organic layer was dried over Na_2_SO_4_ and concentrated in vacuo. The crude product was washed with acetonitrile at 60 °C (50 mL × 3) to obtain 2,5-di(dococyloxy)benzaldehyde in 82% yield (2.1 g, 3 mmol). ^1^H NMR (400 MHz, CDCl_3_) δ (ppm): 10.47 (s, 1H), 7.31 (d, *J* = 3.2 Hz, 1H), 7.11 (dd, *J* = 9.1, 3.2 Hz, 1H), 6.92 (d, *J* = 9.1 Hz, 1H), 4.02 (t, *J* = 6.5 Hz, 2H), 3.94 (t, *J* = 6.6 Hz, 2H), 1.85–1.72 (m, 4H), 1.50–1.19 (m, 76H), 0.88 (t, *J* = 6.7 Hz, 6H). ^13^C NMR (101 MHz, CDCl_3_) δ (ppm): 189.91, 156.47, 153.18, 125.24, 124.27, 114.53, 110.97, 69.38, 68.84, 32.08, 29.86, 29.81, 29.75, 29.71, 29.54, 29.52, 29.38, 26.22, 26.15, 22.85, 22.14, 22.09, 14.27.

2,5-di(dococyloxy)benzaldehyde (2.1 g, 3 mmol, 1 eq.) was dissolved with THF (0.03 M) and 2-propanol (0.3 M), followed by the addition of NaBH_4_ (0.2 g, 5.5 mmol, 2 eq.). The resulting reaction mixture was stirred at room temperature for 60 min, followed by dilution with brine (55 mL). The aqueous layer was extracted with THF (25 × 3 mL), dried over Na_2_SO_4_, and concentrated in vacuo. The crude product was washed with MeOH (100 mL) to obtain (2,5-bis(docosyloxy)phenyl)methanol in 97% yield (2.2 g, 2.9 mmol). Analytical characterization was fully consistent with data in the literature [31]. ^1^H NMR (400 MHz, CDCl_3_) δ (ppm): 6.85 (s, 1H), 6.79–6.74 (m, 2H), 4.65 (s, 2H), 3.95 (t, *J* = 6.6 Hz, 2H), 3.90 (t, *J* = 6.6 Hz, 2H), 1.76 (hept, *J* = 7.4 Hz, 4H), 1.48–1.11 (m, 76H), 0.88 (t, *J* = 6.5 Hz, 6H). ^13^C NMR (101 MHz, CDCl_3_) δ 153.19, 151.11, 130.32, 115.57, 113.96, 112.27, 68.81, 68.74, 62.62, 32.09, 29.86, 29.82, 29.77, 29.57, 29.52, 26.33, 26.21, 22.85, 14.28. 

#### 5.3.4. Full LPPS of Leu-Enkephaline *via* Fmoc Chemistry 

##### Loading of the First Amino Acid on the Tag: Synthesis of H2N-Leu-Tag 

To a solution of (2,5-bis(docosyloxy)phenyl)methanol (500 mg, 0.66 mmol, 1 eq.) in DCM (100 g/L), *N*-Fmoc-Leu-OH (582 mg, 1.7 mmol, 2.5 eq), DIC (128 µL, 0.8 mmol, 1.25 eq.), and DMAP (8 mg, 0.07 mmol, 0.1 eq) were added. The resulting reaction mixture was stirred at room temperature for 1 h and was monitored using TLC (Hex:EtOAC 9:1; Rf = 0.5), followed by dilution with ACN (10 mL). The resulting precipitate was recovered by vacuum filtration and washed with ACN (5 mL × 3) to obtain the *N*-Fmoc-Leu-Tag in 94% yield (680 mg, 0.62 mmol). ^1^H NMR (400 MHz, CDCl_3_) δ (ppm): 7.83–7.64 (m, 6H), 7.60 (d, *J* = 7.4 Hz, 2H), 7.40 (t, *J* = 7.4 Hz, 2H), 7.31 (t, *J* = 7.5 Hz, 2H), 6.89 (s, 1H), 6.78 (s, 2H), 5.22 (d, *J* = 34.5 Hz, 2H, NH), 4.51–4.34 (m, 3H), 4.23 (t, *J* = 7.2 Hz, 1H), 3.94–3.82 (m, 4H), 1.81–1.66 (m, 6H), 1.43–1.16 (m, 77H), 0.96–0.88 (m, 12H). ^13^C NMR (101 MHz, CDCl_3_) δ (ppm): 173.14, 156.06, 153.01, 151.15, 144.12, 143.89, 141.43, 127.79, 127.18, 125.26, 125.22, 124.88, 120.07, 120.06, 116.17, 115.05, 112.70, 69.01, 68.77, 67.11, 62.81, 52.79, 47.35, 42.09, 32.07, 29.85, 29.81, 29.77, 29.74, 29.58, 29.53, 29.51, 26.22, 26.20, 24.87, 23.00, 22.83, 22.05, 14.25. 

*N*-Fmoc-Leu-Tag (680 mg, 0.62 mmol, 1 eq.) was solubilized with DCM or anisole (100 g/L), and piperidine (1 mL, 10.6 mmol, 16 eq) was added. After 30 min, the reaction was monitored by TLC (Hex:EtOAC 9:1; Rf = 0.2), and the solution was diluted with ACN (10 mL). The resulting precipitates were recovered through vacuum filtration and washed with ACN (5 mL × 3) to obtain the H_2_N-Leu-Tag in 99% yield (524 mg, 0.61 mmol). ^1^H NMR (600 MHz, CDCl_3_) δ (ppm): 6.88 (s, 1H), 6.81–6.74 (m, 2H), 5.17 (s, 2H), 3.92–3.88 (m, 4H), 3.51 (dd, *J* = 5.8, 2.7 Hz, 1H), 1.82–1.72 (m, 6H), 1.62–1.58 (m, 2H), 1.47–1.41 (m, 6H), 1.35–1.25 (m, 69H), 0.94–0.87 (m, 12H). ^13^C NMR (151 MHz, CDCl_3_) δ (ppm): 176.78, 153.01, 151.23, 125.39, 116.29, 114.84, 112.76, 69.06, 68.83, 68.76, 62.27, 53.15, 44.20, 32.08, 29.86, 29.83, 29.81, 29.77, 29.75, 29.58, 29.56, 29.54, 29.52, 26.24, 26.22, 24.91, 23.15, 22.85, 22.04, 14.27. 

##### Elongation of the Peptide: Protocol with Precipitation after Each Step

H_2_N-Leu-tag was dissolved with DCM or anisole (100 g/L), and *N*-Fmoc-Phe-OH (1 eq) was added, followed by the addition of DIPEA (4 eq) and T3P^®^ (50% in DCM; 1.5 eq) in this order. After 5 min, the reaction was monitored through TLC (Hex:EtOAC 8:2; Rf = 0.5), and the solution was diluted with ACN (10 mL). The resulting precipitates were recovered through vacuum filtration and washed with ACN (5 mL × 3), enabling the *N*-Fmoc-Phe-Leu-Tag in 91–98% yield as a white solid. ^1^H NMR (400 MHz, CDCl_3_) δ (ppm): 7.76 (d, *J* = 7.6 Hz, 2H), 7.56–7.53 (m, 2H), 7.40 (t, *J* = 7.4 Hz, 2H), 7.31 (t, *J* = 7.4 Hz, 2H), 7.27–7.17 (m, 5H), 6.86 (s, 1H), 6.82–6.76 (m, 2H), 6.11 (s, NH), 5.35 (s, NH), 5.17 (s, 2H), 4.63–4.58 (m, 1H), 4.46–4.41 (m, 2H), 4.33–4.29 (m, 1H), 4.19 (t, *J* = 6.9 Hz, 2H), 3.92–3.87 (m, 4H), 3.11 (s, 1H), 3.03 (s, 1H), 1.79–1.11 (m, 83H), 0.90–0.85 (m, 12H). ^13^C NMR (101 MHz CDCl_3_) δ (ppm): 172.31, 170.44, 153.00, 151.11, 143.91, 143.84, 141.43, 129.54, 128.83, 127.87, 127.22, 125.16, 124.79, 120.11, 116.23, 115.00, 112.66, 77.48, 77.16, 76.84, 69.00, 68.79, 67.21, 62.78, 47.26, 41.80, 32.08, 29.86, 29.81, 29.79, 29.76, 29.60, 29.52, 26.22, 24.84, 22.85, 22.21, 22.19, 22.17, 22.16, 22.14, 14.28. 

*N*-Fmoc-Phe-Leu-Tag was solubilized in DCM or anisole (100 g/L), and piperidine (16 eq) was added. The solution was stirred for 30 min (TLC: Hex:EtOAC 8:2; Rf = 0.2), followed by dilution with ACN (10 mL). The resulting precipitates were recovered by vacuum filtration and washed with ACN (10 mL × 3) to obtain the H-Phe-Leu-Tag in 98–99% yield. ^1^H NMR (600 MHz, CDCl_3_) δ(ppm): 7.70 (d, *J* = 8.5 Hz, NH), 7.33–7.29 (m, 2H), 7.25–7.21 (m, 2H), 6.89–6.86 (m, 1H), 6.81–6.76 (m, 2H), 5.18 (dd, *J* = 17.1, 12.4 Hz, 2H), 4.72–4.64 (m, 1H), 3.90 (q, *J* = 6.6 Hz, 4H), 3.65 (dd, *J* = 9.2, 4.0 Hz, 1H), 3.25 (dd, *J* = 13.7, 4.0 Hz, 1H), 2.73 (dd, *J* = 13.7, 9.2 Hz, 1H), 1.78–1.70 (m, 4H), 1.63–1.53 (m, 3H), 1.46–1.39 (m, 4H), 1.35–1.26 (m, 72H), 0.94–0.86 (m, 12H). ^13^C NMR (151 MHz, CDCl_3_) δ (ppm): 174.11, 172.98, 153.22, 153.04, 151.12, 151.08, 137.91, 129.48, 128.84, 126.98, 125.14, 116.03, 114.88, 112.70, 69.06, 68.82, 68.76, 62.62, 62.58, 56.51, 50.66, 41.76, 40.99, 32.08, 29.86, 29.81, 29.78, 29.76, 29.60, 29.56, 29.54, 29.51, 26.32, 26.23, 26.22, 26.20, 24.99, 23.03, 22.84, 22.12, 14.27. 

The above procedure was repeated until H_2_N-Tyr(tBu)-Gly-Gly-Phe-Leu-tag was obtained.

##### Elongation of the Peptide: Protocol with One-Pot Coupling–Deprotection Steps

H_2_N-Leu-tag was dissolved with DCM or anisole (100 g/L), and *N*-Fmoc-Phe-OH (1 eq) was added, followed by the addition of DIPEA (3 eq) and T3P^®^ (50% in DCM; 1.5 eq) in this order. After 5 min, the reaction was completed, and piperidine (16 eq) was added. The solution was stirred for 30 min followed by dilution with ACN (10 mL). The resulting precipitates were recovered through vacuum filtration and washed with ACN (10 mL × 3) to obtain the *N*-Fmoc-Leu-Tag in 98–99% yield. The above procedure was repeated until H_2_N-Tyr(tBu)-Gly-Gly-Phe-Leu-tag was obtained.

*N-Fmoc-Gly-Gly-Phe-Leu-OTag* (Y = 89–94%): TLC Hex:EtOAc (1:1) Rf = 0.2. ^1^H NMR (600 MHz, CDCl_3_) δ (ppm): 7.74 (d, *J* = 7.6 Hz, 2H), 7.60–7.57 (m, 2H), 7.38 (t, *J* = 7.5 Hz, 2H), 7.30–7.27 (m, 2H), 7.23–7.14 (m, 5H), 6.86 (s, 1H), 6.79–6.72 (m, 2H, NH), 5.63 (bs, NH), 5.15 (dd, *J* = 25.7, 12.7 Hz, 2H), 4.80 (bs, NH), 4.61–4.58 (m, 1H), 4.47–4.39 (m, 2H), 4.21–4.19 (m, 1H), 3.98–3.73 (m, 8H), 3.10–3.01 (m, 2H), 1.80–1.48 (m, 10H), 1.43–1.25 (m, 73H), 0.92–0.82 (m, 12H). ^13^C NMR (151 MHz, CDCl_3_) δ (ppm): 172.49, 170.44, 169.53, 168.33, 156.84, 153.00, 151.12, 151.09, 143.88, 143.80, 141.47, 136.39, 129.49, 128.74, 128.71, 127.89, 127.23, 127.16, 125.17, 124.89, 120.14, 116.26, 115.59, 114.92, 114.03, 113.98, 112.67, 112.30, 69.02, 68.83, 68.76, 67.38, 62.72, 62.60, 54.45, 51.24, 47.26, 44.54, 43.22, 41.61, 38.63, 38.52, 32.08, 29.86, 29.81, 29.76, 29.61, 29.57, 29.54, 29.51, 26.32, 26.21, 26.20, 24.88, 22.84, 22.13, 14.27.

*H_2_N-Gly-Gly-Phe-Leu-OTag* (Y = 92–94%): TLC Hexane:EtOAc (2:8) Rf = 0.2. ^1^H NMR (400 MHz, 20%MeOD in CDCl_3_) δ (ppm): 7.09–6.99 (m, 5H), 6.77–6.63 (m, 3H), 5.03–4.94 (m, 2H), 4.48–4.45 (m, 1H), 4.38–4.35 (m, 1H), 3.82–3.62 (m, 6H), 3.51 (s, 2H), 2.95–2.91 (m, 1H), 2.74 (dd, *J* = 13.6, 8.1 Hz, 1H), 1.69–1.38 (m, 10H), 1.36–1.04 (m, 83H), 0.75–0.65 (m, 12H). ^13^C NMR (151 MHz, 20%MeOD in CDCl_3_) δ (ppm): 172.30, 171.06, 168.82, 152.60, 150.77, 136.31, 129.00, 128.17, 126.57, 124.50, 115.99, 114.56, 112.37, 68.70, 68.52, 62.32, 60.38, 54.09, 50.77, 42.21, 40.77, 37.79, 31.67, 29.45, 29.36, 29.17, 29.11, 25.79, 24.47, 22.42, 22.37, 21.48, 13.77.

*N-Fmoc-Tyr-Gly-Gly-Phe-Leu-OTag* (Y = 75–80%): TLC Hexane:EtOAc (3:7) Rf = 0.5. ^1^H NMR (400 MHz, 20%MeOD in CDCl_3_) δ (ppm): 7.84 (s, NH), 7.58–7.52 (m, 2H, NH), 7.46 (s, NH), 7.39–7.31 (m, 2H, NH), 7.20 (t, *J* = 7.4 Hz, 2H), 7.11 (t, *J* = 7.5 Hz, 2H), 7.05–6.98 (m, 5H), 6.92 (d, *J* = 8.0 Hz, 2H), 6.71–6.68 (m, 3H), 6.60 (s, 2H), 6.52 (d, *J* = 7.2 Hz, NH), 4.96 (q, *J* = 12.4 Hz, 2H), 4.47–4.35 (m, 2H), 4.19–4.13 (m, 3H), 3.96 (t, *J* = 7.0 Hz, 1H), 3.73–3.50 (m, 8H), 2.93 (dd, *J* = 14.1, 6.2 Hz, 2H), 2.79–2.71 (m, 2H), 1.59–1.36 (m, 8H), 1.25–1.07 (m, 84H), 0.71–0.66 (m, 12H). ^13^C NMR (151 MHz, 20%MeOD in CDCl_3_) δ (ppm): 172.76, 172.39, 171.13, 169.95, 169.35, 156.76, 154.04, 152.76, 150.94, 143.64, 141.21, 136.43, 131.48, 129.64, 129.37, 129.20, 128.35, 127.68, 127.05, 126.75, 124.95, 124.72, 124.22, 119.87, 116.11, 114.77, 112.58, 78.67, 68.88, 68.70, 67.03, 62.46, 60.88, 56.57, 54.25, 50.99, 46.99, 42.77, 42.39, 40.90, 37.85, 37.09, 31.84, 29.61, 29.52, 29.35, 29.28, 28.59, 25.95, 24.60, 22.59, 22.54, 21.65, 13.94.

*H_2_N-Tyr-Gly-Gly-Phe-Leu-OTag* (Y = 90–96%): TLC Hexane:EtOAc (1:9) Rf = 0.2. ^1^H NMR (400 MHz, CDCl_3_) δ 8.08 (s, NH), 7.37 (s, NH), 7.26–7.17 (m, 5H), 7.08 (d, *J* = 8.3 Hz, 2H), 6.97–6.86 (m, 3H, 2NH), 6.77 (s, 2H), 5.16 (dd, *J* = 22.3, 12.8 Hz, 2H), 4.76 (q, *J* = 7.0 Hz, 1H), 4.53 (s, 1H), 4.01–3.67 (m, 9H), 3.19–3.03 (m, 3H), 2.74–2.68 (m, 1H), 1.77–1.52 (m, 8H), 1.41–1.16 (m, 84H), 0.89–0.86 (m, 12H). ^13^C NMR (151 MHz, CDCl_3_) δ 172.49, 171.00, 169.69, 168.93, 154.51, 153.01, 151.05, 136.70, 129.83, 129.52, 128.65, 127.03, 124.92, 124.48, 116.21, 114.85, 112.66, 78.55, 69.01, 68.81, 62.67, 56.29, 54.66, 51.32, 44.43, 43.57, 43.08, 41.42, 38.42, 32.06, 29.84, 29.75, 29.60, 29.50, 28.97, 26.20, 26.18, 24.84, 22.87, 22.82, 14.25.

##### Peptide Cleavage from the Tag

In an oven-dried Schlenk tube, the fully protected peptide-tag was dissolved in a cleavage cocktail (200 g/mL) composed of: 95.0% TFA, 2.5% H_2_O, and 2.5% TIS. The reaction mixture was stirred at room temperature for 2 h under a nitrogen atmosphere, and then DIPE (V_DIPE_ = 8V_TFA_) was added dropwise, inducing the formation of a white solid. After the complete addition of the solvent, the reaction mixture was cooled to 0 °C and slowly stirred for 30 min. Subsequently, the white solid was recovered by filtration. The purity of the target peptide was monitored through HPLC-MS analysis (method B in Chapter 1 of the supporting information). Analytical characterization was fully consistent with data in the literature [26,33].

## Figures and Tables

**Table 1:** Suitability of T3P^®^ protocol in different solvents ^a^.

Entry	PG	Solvent	Product	Conversion ^b^ (%)
1	Boc	DMF	**1a**	97
2	Fmoc	DMF	**1b**	>99
3	Boc	DCM	**1a**	>99
4	Fmoc	DCM	**1b**	>99
5	Boc	Anisole	**1a**	98
6	Fmoc	Anisole	**1b**	>99
7	Boc	CPME ^c^	**1a**	82
8	Fmoc	CPME	**1b**	99
9	Boc	EtOAc	**1a**	97
10	Fmoc	EtOAc	**1b**	99
11	Boc	nPrOAc	**1a**	95
12	Fmoc	nPrOAc	**1b**	99
13	Boc	*^t^*BuOAc ^c^	**1a**	85
14	Fmoc	*^t^*BuOAc	**1b**	>99
15	Boc	DMC	**1a**	92
16	Fmoc	DMC	**1b**	>99
17	Boc	THF	**1a**	96
18	Fmoc	THF	**1b**	>99
19	Boc	ACN	**1a**	93
20	Fmoc	ACN	**1b**	>99
21	Boc	NOP	**1a**	57
22	Fmoc	NOP	**1b**	97

^a^ The reactions were performed with dry solvents under a nitrogen atmosphere. ^b^ Conversion calculated on the basis of the HPLC peaks area, comparing the UV signal at 220 nm of H_2_N-Leu-OBn with the dipeptide without an RRF correction. ^c^ The presence of undissolved material was observed.

**Table 2:** Synthesis of *N*-Boc/*N*-Fmoc dipeptides under optimized conditions ^a^.

Entry	*N*-PG-Amino Acid	Amino Ester	Conversion ^b^ (%)
1	*N*-Boc-Leu-OH	H-Leu-OMe	98
2	*N*-Boc-Phe-OH	H-Leu-OMe	98
3	*N*-Boc-Arg(Pbf)-OH	H-Leu-OMe	99
4	*N*-Boc-Asp(Bn)-OH	H-Leu-OMe	96
5	*N*-Boc-Ser-OH	H-Leu-OMe	>99
6	*N*-Boc-Thr-OH	H-Leu-OMe	99
7	*N*-Boc-Trp-OH	H-Leu-OMe	96
8	*N*-Boc-Aib-OH	H-Leu-OMe	>99
9	*N*-Fmoc-Leu-OH	H-Leu-OBn	>99
10	*N*-Fmoc-Trp-OH	H-Leu-OBn	>99
11	*N*-Fmoc-Arg(Pbf)-OH	H-Leu-OBn	>99
12	*N*-Fmoc-Asp(*^t^*Bu)-OH	H-Leu-OBn	>99
13	*N*-Fmoc-Ser(*^t^*Bu)-OH	H-Leu-OBn	>99
14	*N*-Fmoc-Thr(*^t^*Bu)-OH	H-Leu-OBn	>99
15	*N*-Fmoc-Arg-OH	H-Leu-OBn	98
16	*N*-Fmoc-Aib-OH	H-Leu-OBn	99

^a^ The reactions were performed in DCM under a nitrogen atmosphere. ^b^ Conversion calculated on the basis of the HPLC peaks area, comparing the UV signal at 220 nm of the *N*-Boc-and *N*-Fmoc-amino acid with the dipeptide without an RRF correction.

**Table 3:** Comparison of LPPS of Leu-Enkephalin following different protocols.

Solvent	DCM	DCM	Anisole	Anisole
Base (eq)	PIP (16)	PIP (16)	PIP (16)	PIP (16)
Precipitation	Each step	After depr only	Each step	After depr only
TM	>99	>99	95.9	91.4
des-Phe	-	-	-	-
des-Tyr	-	-	4.1	8.6
des-Gly_2_	-	-	-	-
Leu-Enkephalin purity	**>99**	**>99**	**95.9**	**91.4**

## Data Availability

The data presented in this study are available in the Supplementary Materials file.

## Supplementary Materials

The following supporting information can be downloaded at: https://www.mdpi.com/article/10.3390/molecules28207183/s1, LC-MS chromatograms for all the tables’ entries, complete characterization of isolated intermediates.
