# Supplementary material for: Fast Solution-Phase and Liquid-Phase Peptide Syntheses (SolPSS and LPPS) Mediated by Biomimetic Cyclic Propylphosphonic Anhydride (T3P®)
Source: Molecules. 2023 Oct 19;28(20):7183. doi: 10.3390/molecules28207183 (PMC10609394; doi:10.3390/molecules28207183)
Supplement: Supplementary file 1 [file molecules-28-07183-s001.zip › molecules-2663184-supplementary.pdf]

Fast Solution-Phase and Liquid-Phase Peptide Syntheses (SolPSS and LPPS) Mediated by Biomimetic Cyclic Propylphosphonic Anhydride (T3P®)

Alexia Mattellone [1], Dario Corbisiero [1], Paolo Cantelmi [1], Giulia Martelli [1], Chiara Palladino [1], Alessandra Tolomelli [1,2,*], Walter Cabri [1,2,*] and Lucia Ferrazzano [1]

[1] Tolomelli-Cabri/P4I Lab—Peptidomimetics and Peptides Targeting Protein-Protein Interaction, Department of Chemistry "Giacomo Ciamician", Alma Mater Studiorum—University of Bologna, Via Gobetti 87, 40129 Bologna, Italy; alexia.mattellone2@unibo.it (A.M.); dario.corbisiero2@unibo.it (D.C.); pa.cantelmi@gmail.com (P.C.); giulia.martelli8@unibo.it (G.M.); chiara.palladino5@unibo.it (C.P.); lucia.ferrazzano4@unibo.it (L.F.)

[2] Consorzio C.I.N.M.P.I.S. (National Interuniversity Research Consortium in Innovative Synthesis Methodologies and Processes) c/o, Alma Mater Studiorum—University of Bologna, Via Gobetti 87, 40129 Bologna, Italy

* Correspondence: alessandra.tolomelli@unibo.it (A.T.); walter.cabri@unibo.it (W.C.); Tel.: +39-0512099575 (A.T.)

Table of Contents

1. HPLC Methods S2
2. HPLC Chromatogram: Solvent suitability S2
3. HPLC Chromatogram: Substrate scope S10
4. Racemization evaluation S16
5. HPLC chromatogram: SolPPS of Leu-Enkephalin precursor *via N*-Boc chemistry S17
6. HPLC Chromatograms: Full LPPS of Leu-Enkephalin via *N*-Fmoc chemistry S22
    6.1 Epimers of Leu-Enkephalin synthetized by SPPS S24
7. HPLC Chromatograms: Solvents and amino acids S25
8. NMR spectra S31

1. HPLC Methods

The gradient of analytical methods reported across the paper are defined as follow:

**Method A**		
Iorz =4 p o2p lq		
Wlp h +p lq,	P relh skdvh D +(,	P relh skdvh E +(,
3	<3	43
48	43	<3
4;	43	<3
63	<3	43

**Method B**		
Iorz =318 p o2p lq		
Wlp h +p lq,	P relh skdvh D +(,	P relh skdvh E +(,
3	;3	53
43	93	73
53	93	73
58	:3	63
63	:3	63

**Method C**		
Iorz =4 p o2p lq		
Wlp h +p lq,	P relh skdvh D +(,	P relh skdvh E +(,
3	;3	53
5	;3	53
43	53	;3
4;	53	;3
4<	8	<8
68	8	<8
73	;3	53
75	;3	53

2. HPLC Chromatogram: Solvent suitability

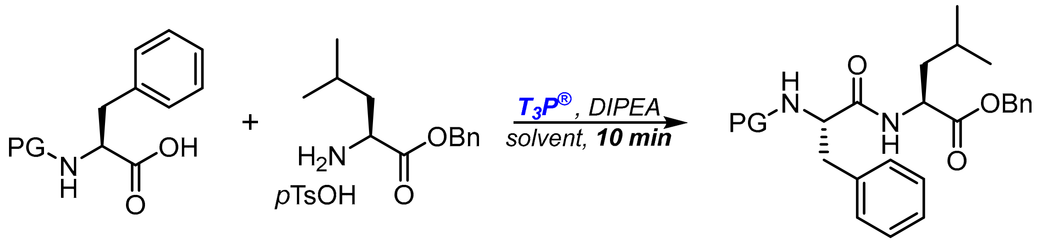

1a. *PG = Boc*
1b. *PG = Fmoc*

Scheme S1. Model reaction for T3P® induced peptide synthesis.

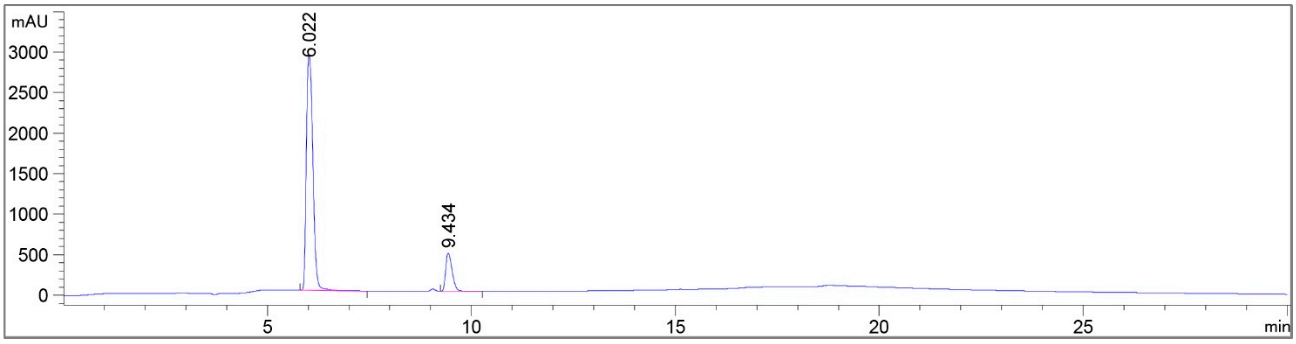

Figure S1. Chromatogram of H_2N-Leu-OBn at 220 nm (reference). The peak at 6.022 min is associated with p-Toluenesulphonic acid, as the counterion of the commercial leucine benzyl ester, and the peak at 9.434 min is related to H_2*N*-Leu-OBn.

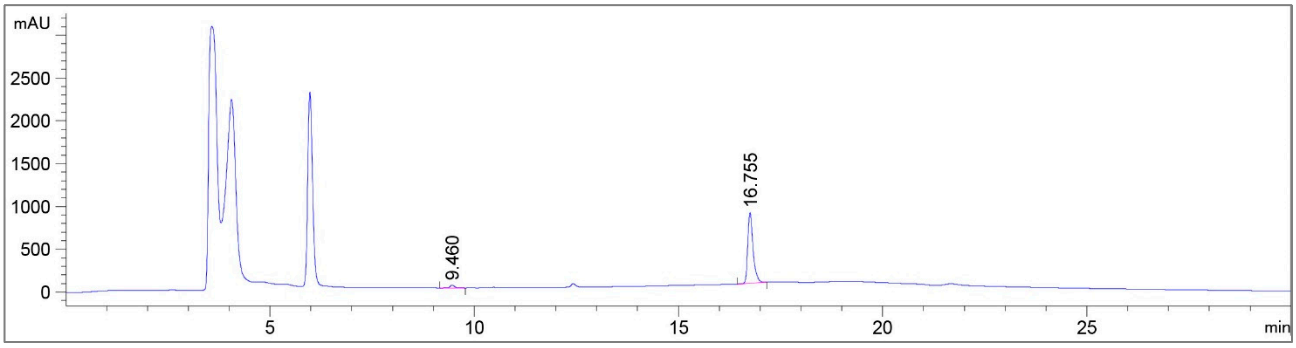

Product	m/z observed	Rt (min)	Area (%)
N-Boc-Phe-Leu- OBn	469.0	16.755	96.8475
H_2N-Leu-OBn	222.1	12.460	3.1525

Figure S2. Chromatogram of *N*-Boc-Phe-Leu-OBn (**1a**) in DMF at 220 nm (entry 1, Table 1 in the article). The peaks around 3 min are associated with DMF (see chapter 7).

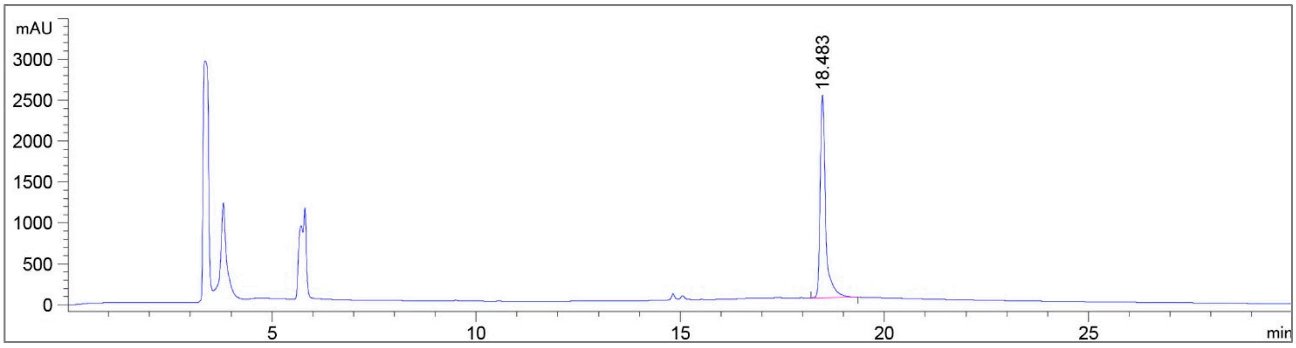

Product	m/z observed	Rt (min)
N-Fmoc-Phe-Leu-OBn	591.3	18.483

Figure S3. Chromatogram of *N*-Fmoc-Phe-Leu-OBn (**1b**) in DMF at 220 nm (entry 2, Table 1 in the article). The peaks around 3 min are associated with DMF (see chapter 7).

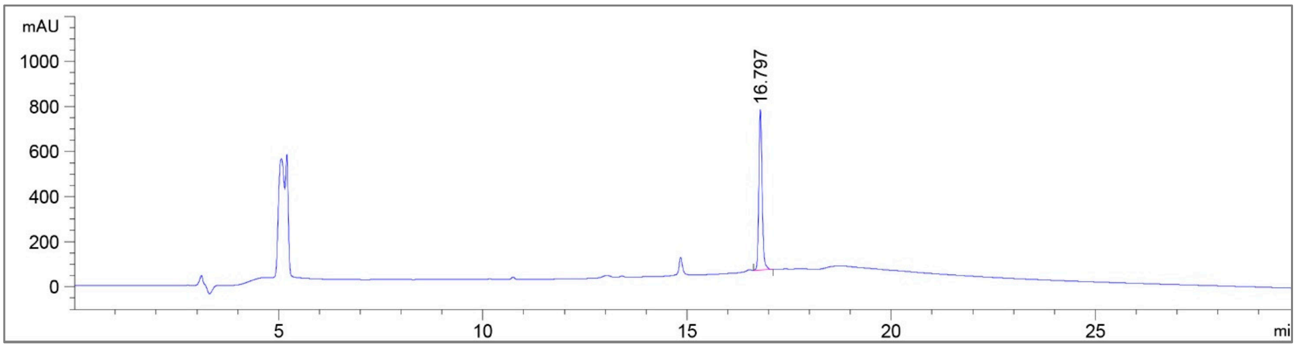

Product	m/z observed	Rt (min)
N-Boc-Phe-Leu-OBn	469.1	16.797

Figure S4. Chromatogram of *N*-Boc-Phe-Leu-OBn (**1a**) in DCM at 220 nm (entry 3, Table 1 in the article).

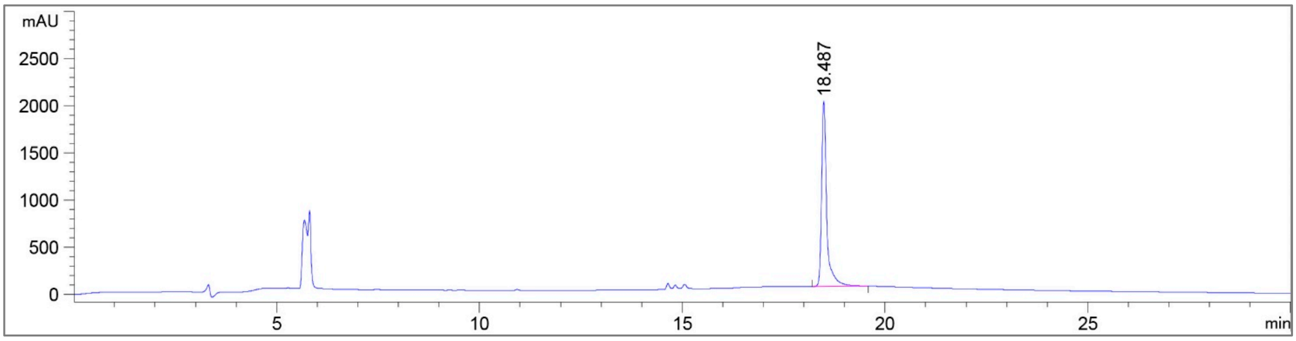

Product	m/z observed	Rt (min)
N-Fmoc-Phe-Leu-OBn	591.3	18.487

Figure S5. Chromatogram of *N*-Fmoc-Phe-Leu-OBn (**1b**) in DCM at 220 nm (entry 4, Table 1 in the article).

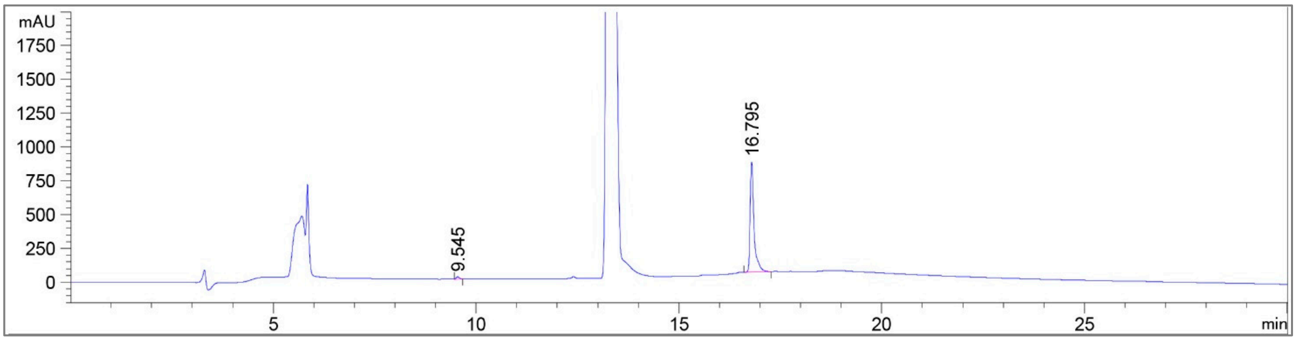

Product	m/z observed	Rt (min)	Area (%)
N-Boc-Phe-Leu-OBn	491.2 [M+Na$^+$]	16.795	97.8563
H_2N-Leu-OBn	222.1	9.545	2.1437

Figure S6. Chromatogram of *N*-Boc-Phe-Leu-OBn (**1a**) in Anisole at 220 nm (entry 5, Table 1 in the article). The peak at 13 min is associated with Anisole (see chapter 7).

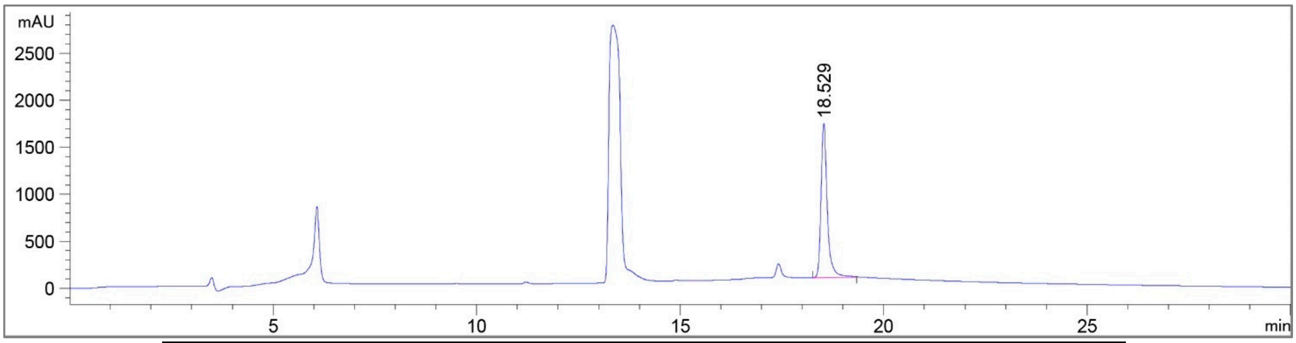

Product	m/z observed	Rt (min)
N-Fmoc-Phe-Leu-OBn	591.2	18.529

Figure S7. Chromatogram of *N*-Fmoc-Phe-Leu-OBn (**1b**) in Anisole at 220 nm (entry 6, Table 1 in the article). The peak at 13 min is associated with Anisole (see chapter 7).

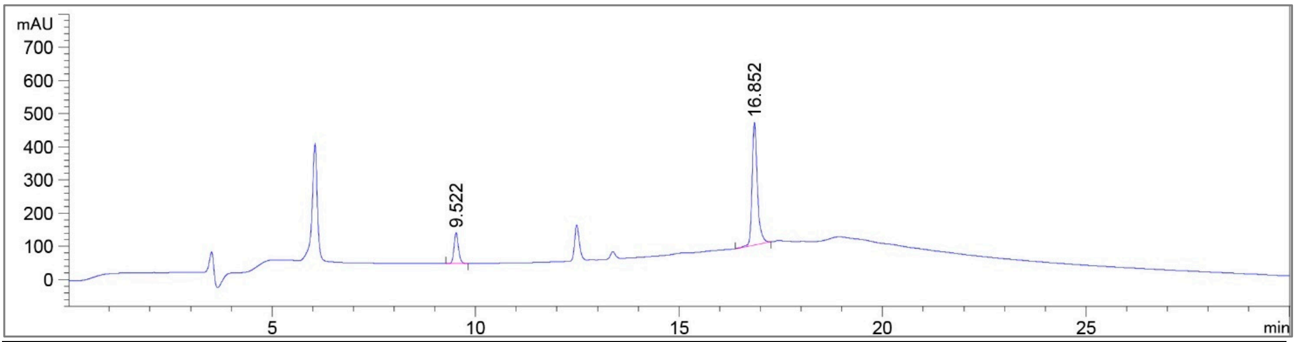

Product	m/z observed	Rt (min)	Area (%)
N-Boc-Phe-Leu-OBn	469.1	16.852	82.1634
H_2N-Leu-OBn	222.1	9.522	17.8366

Figure S8. Chromatogram of *N*-Boc-Phe-Leu-OBn (**1a**) in CPME at 220 nm (entry 7, Table 1 in the article).

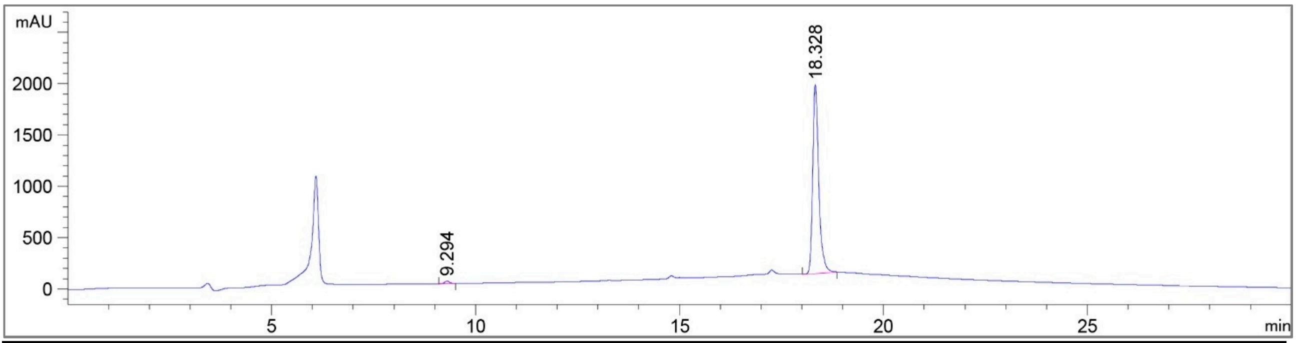

Product	m/z observed	Rt (min)	Area (%)
N-Fmoc-Phe-Leu-OBn	591.2	18.328	99.0193
H_2N-Leu-OBn	222.3	9.294	0.9807

Figure S9. Chromatogram of *N*-Fmoc-Phe-Leu-OBn (**1b**) in CPME at 220 nm (entry 8, Table 1 in the article).

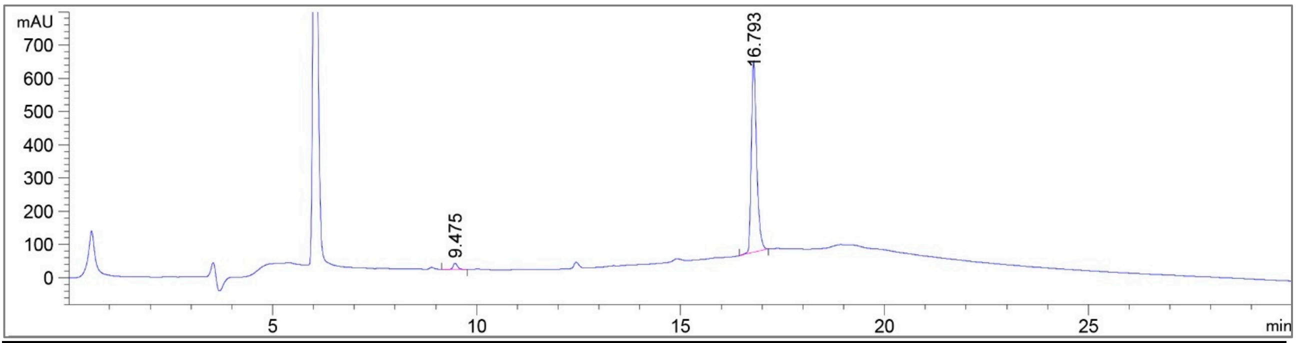

Product	m/z observed	Rt (min)	Area (%)
N-Boc-Phe-Leu-OBn	469.3	16.793	97.2385
H_2N-Leu-OBn	222.2	9.475	2.7615

Figure S10. Chromatogram of *N*-Boc-Phe-Leu-OBn (**1a**) in EtOAc at 220 nm (entry 9, Table 1 in the article).

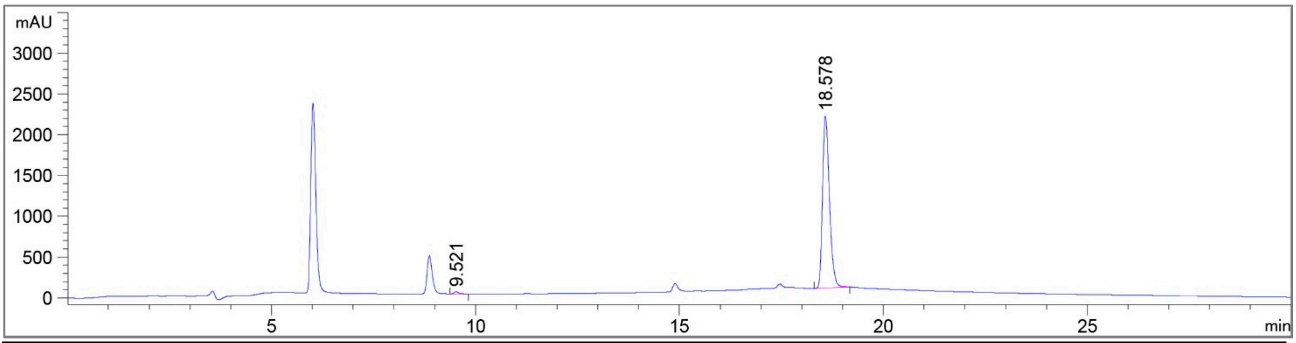

Product	m/z observed	Rt (min)	Area (%)
N-Fmoc-Phe-Leu-OBn	591.1	18.578	99.1823
H_2N-Leu-OBn	222.1	9.521	0.8177

Figure S11. Chromatogram of *N*-Fmoc-Phe-Leu-OBn (**1b**) in EtOAc at 220 nm (entry 10, Table 1 in the article). The peak at 8 min is associated with EtOAc (see chapter 7).

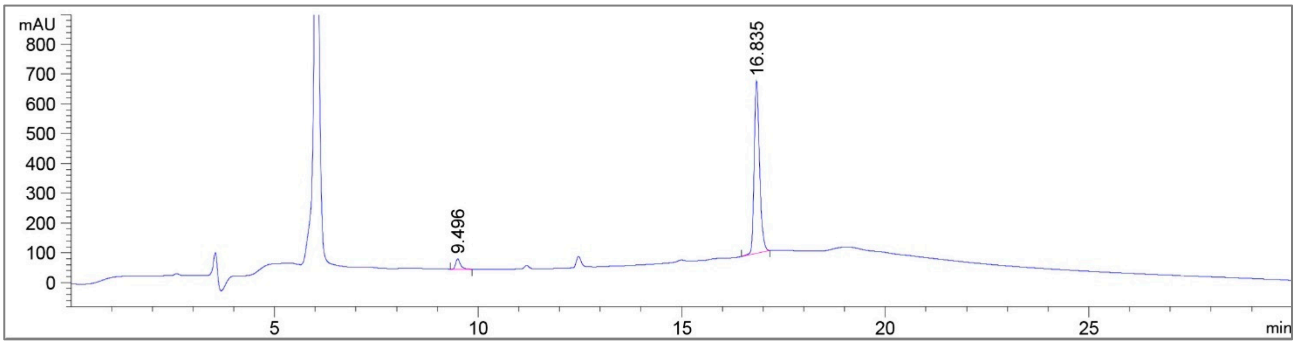

Product	m/z observed	Rt (min)	Area (%)
N-Boc-Phe-Leu-OBn	469.1	16.835	95.0755
H_2N-Leu-OBn	222.4	9.496	4.9245

Figure S12. Chromatogram of *N*-Boc-Phe-Leu-OBn (**1a**) in PrOAc at 220 nm (entry 11, Table 1 in the article).

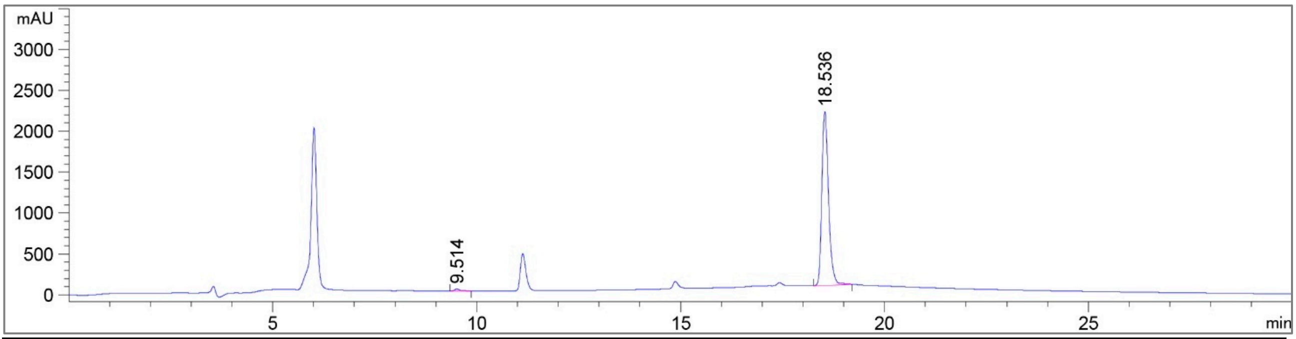

Product	m/z observed	Rt (min)	Area (%)
N-Fmoc-Phe-Leu-OBn	591.6	18.536	99.2164
H_2N-Leu-OBn	222.1	9.514	0.7836

Figure S13. Chromatogram of *N*-Fmoc-Phe-Leu-OBn (**1b**) in PrOAc at 220 nm (entry 12, Table 1 in the article). The peak at 11 min is associated with PrOAc (see chapter 7).

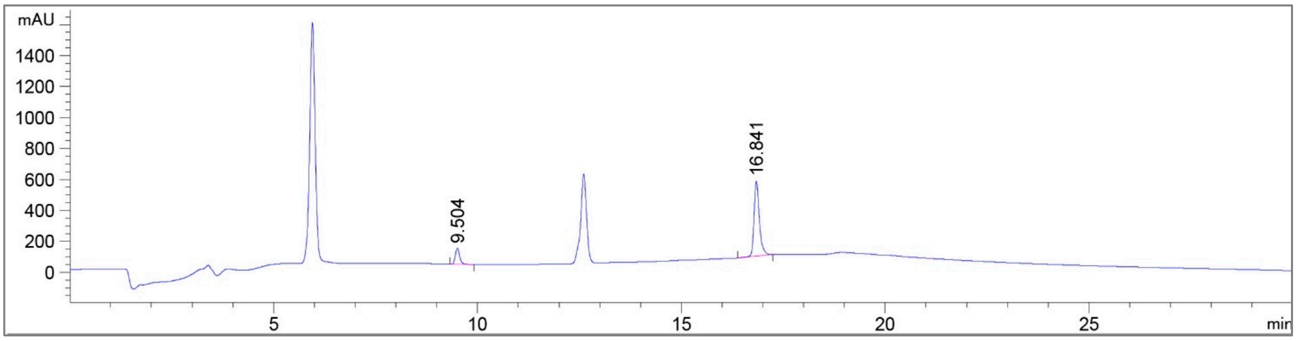

Product	m/z observed	Rt (min)	Area (%)
N-Boc-Phe-Leu-OBn	469.1	16.841	84.9259
H_2N-Leu-OBn	222.1	9.504	15.0741

Figure S14. Chromatogram of *N*-Boc-Phe-Leu-OBn (**1a**) in tBuOAc at 220 nm (entry 13, Table 1 in the article). The peak at 12 min is associated with tBuOAc (see chapter 7).

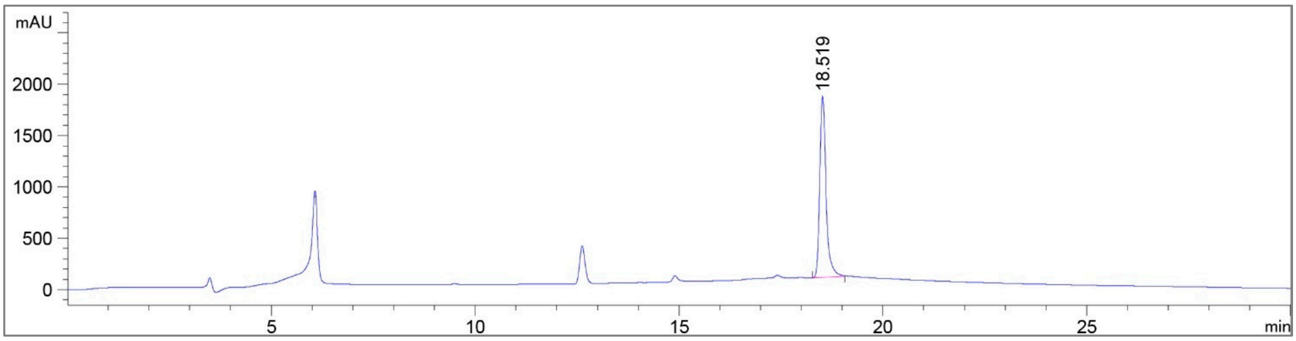

Product	m/z observed	Rt (min)
N-Fmoc-Phe-Leu-OBn	591.1	18.519

Figure S15. Chromatogram of *N*-Fmoc-Phe-Leu-OBn (**1b**) in tBuOAc at 220 nm (entry 14, Table 1 in the article). The peak at 12 min is associated with tBuOAc (see chapter 7).

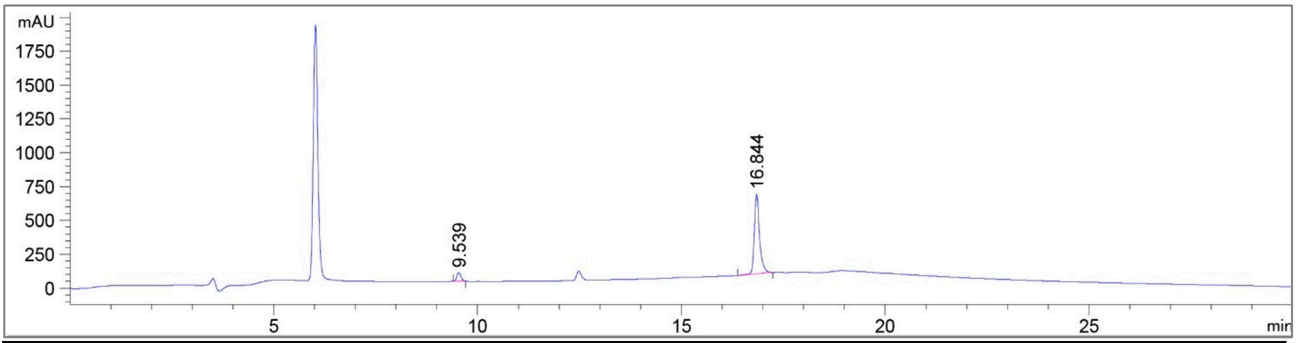

Product	m/z observed	Rt (min)	Area (%)
N-Boc-Phe-Leu-OBn	469.1	16.844	92.0263
H_2N-Leu-OBn	222.0	9.539	7.9737

Figure S16. Chromatogram of *N*-Boc-Phe-Leu-OBn (**1a**) in DMC at 220 nm (entry 15, Table 1 in the article).

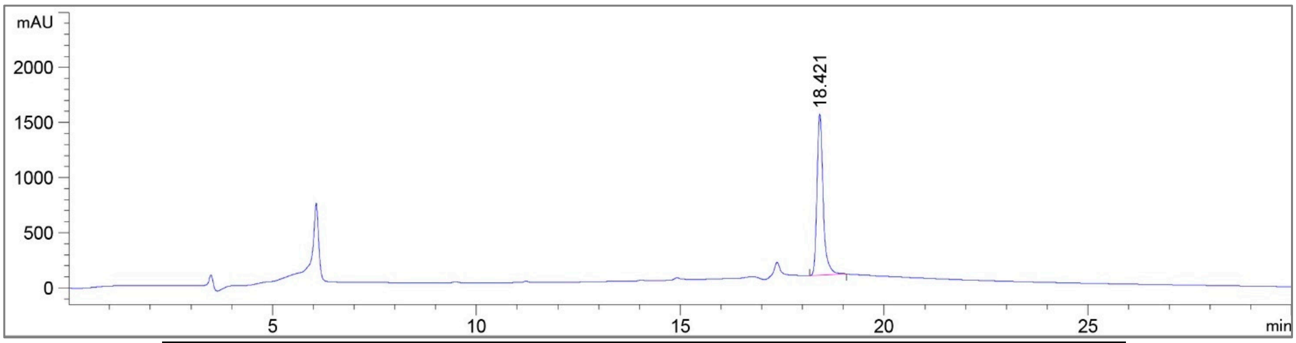

Product	m/z observed	Rt (min)
N-Fmoc-Phe-Leu-OBn	591.2	18.421

Figure S17. Chromatogram of *N*-Fmoc-Phe-Leu-OBn (**1b**) in DMC at 220 nm (entry 16, Table 1 in the article).

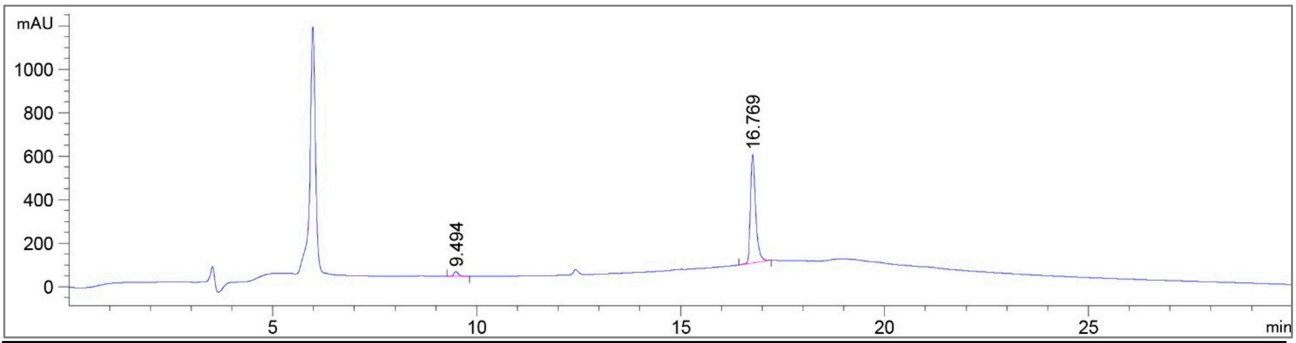

Product	m/z observed	Rt (min)	Area (%)
N-Boc-Phe-Leu-OBn	469.0	16.769	96.2627
H_2N-Leu-OBn	222.3	9.494	3.7373

Figure S18. Chromatogram of *N*-Boc-Phe-Leu-OBn (**1a**) in THF at 220 nm (entry 17, Table 1 in the article).

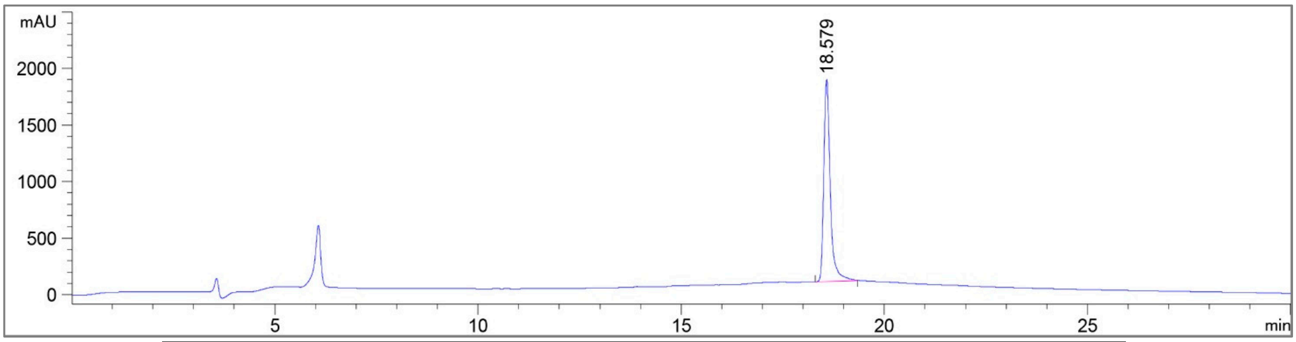

Product	m/z observed	Rt (min)
N-Fmoc-Phe-Leu-OBn	591.4	18.579

Figure S19. Chromatogram of *N*-Fmoc-Phe-Leu-OBn (**1b**) in THF at 220 nm (entry 18, Table 1 in the article).

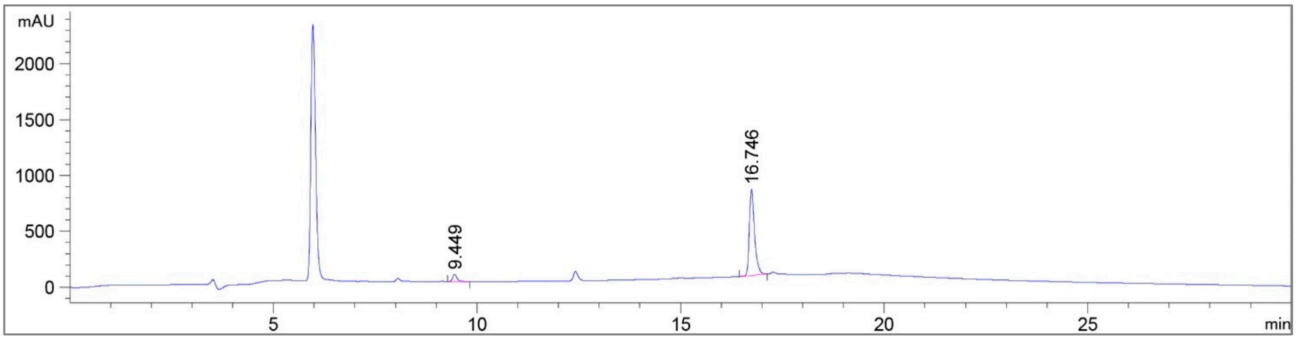

Product	m/z observed	Rt (min)	Area (%)
N-Boc-Phe-Leu-OBn	469.2	16.746	93.2679
H_2N-Leu-OBn	222.1	9.449	6.7321

Figure S20. Chromatogram of *N*-Boc-Phe-Leu-OBn (**1a**) in ACN at 220 nm (entry 19, Table 1 in the article).

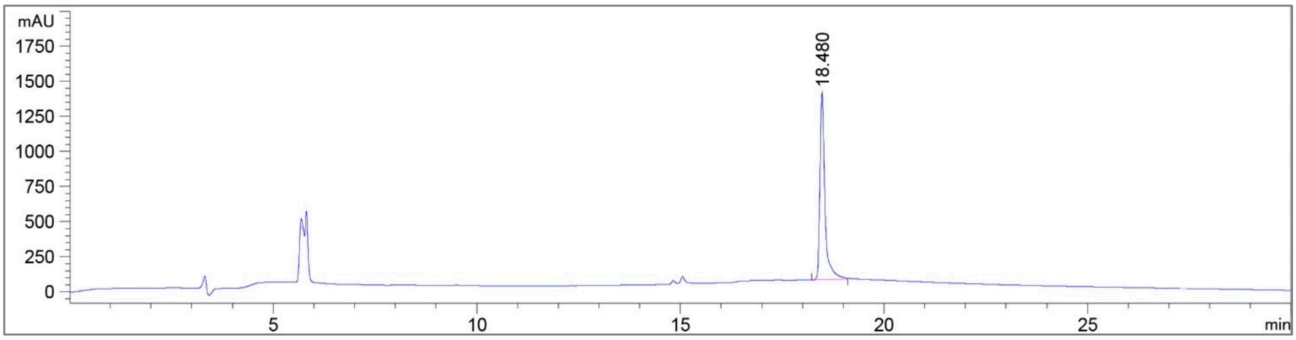

Product	m/z observed	Rt (min)
N-Fmoc-Phe-Leu-OBn	591.2	18.480

Figure S21. Chromatogram of *N*-Fmoc-Phe-Leu-OBn (**1b**) in ACN at 220 nm (entry 20, Table 1 in the article).

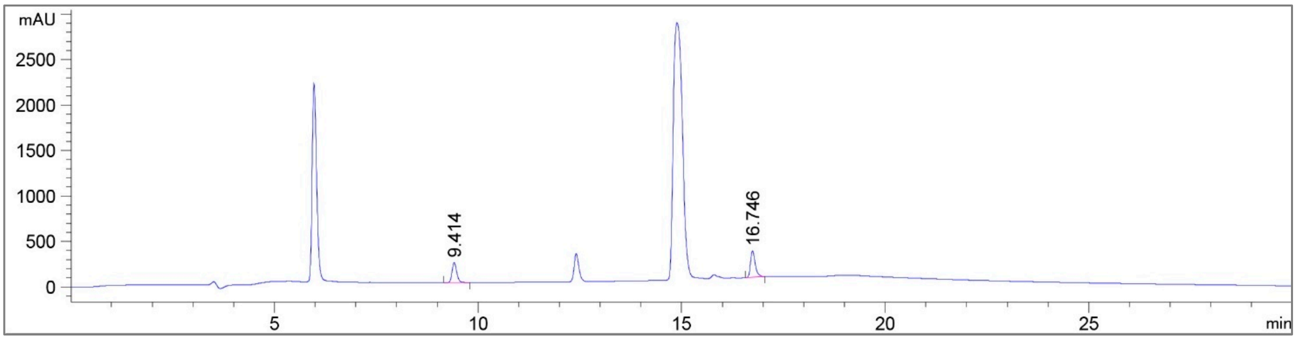

Product	m/z observed	Rt (min)	Area (%)
N-Boc-Phe-Leu-OBn	469.1	16.746	56.8488
H_2N-Leu-OBn	222.0	9.414	43.1512

Figure S22. Chromatogram of *N*-Boc-Phe-Leu-OBn (**1a**) in NOP at 220 nm (entry 21, Table 1 in the article). The peak at 15 min is associated with NOP (see chapter 7).

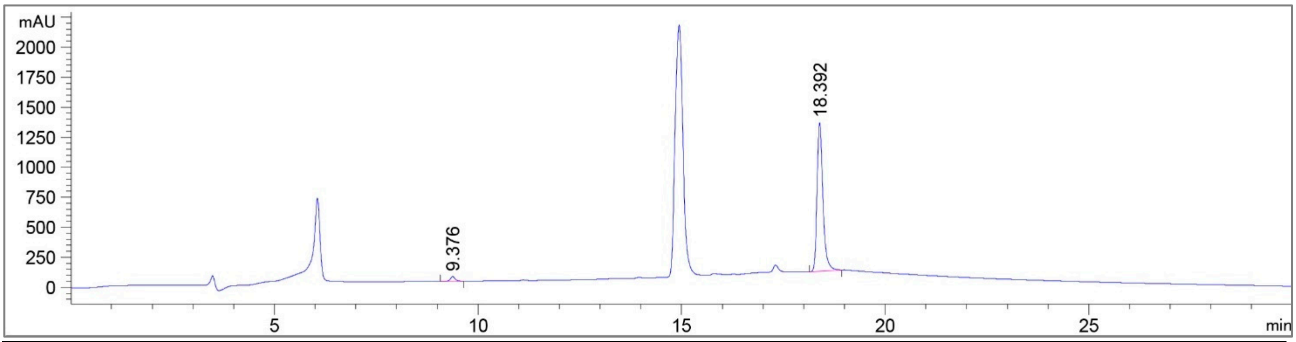

Product	m/z observed	Rt (min)	Area (%)
N-Fmoc-Phe-Leu-OBn	591.3	18.579	97.4887
H_2N-Leu-OBn	222.0	9.376	2.5113

Figure S23. Chromatogram of *N*-Fmoc-Phe-Leu-OBn (**1b**) in NOP at 220 nm (entry 22, Table 1 in the article). The peak at 15 min is associated with NOP (see chapter 7).

3. HPLC Chromatogram: Substrate scope

Chromatograms

The PG-amino acids were injected into the HPLC-MS with the same methods used for each reaction. In addition, for the reaction of 12, 13 and 14 entries (Table S2) samples were analyzed using a different method (Method C) due to elution difficulties.

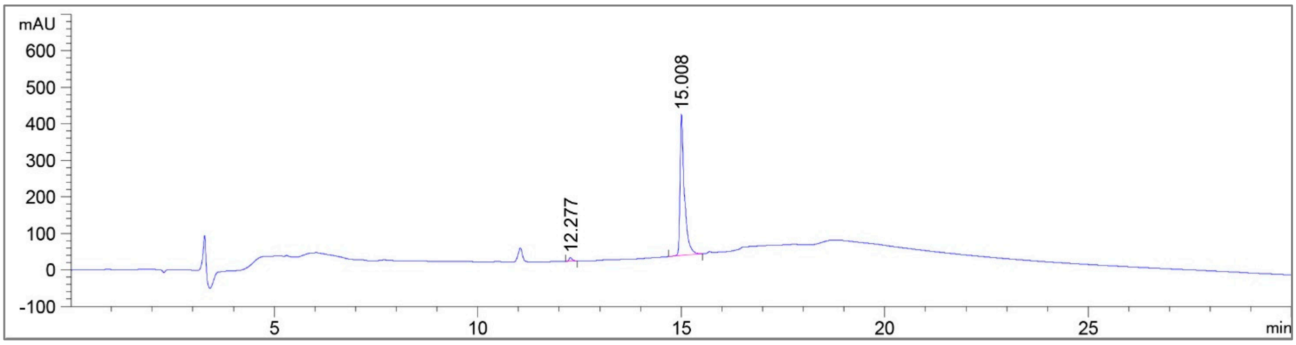

Product	m/z observed	Rt (min)	Area (%)
N-Boc-Leu-Leu-OMe	359.2	15.008	98.1632
N-Boc-Leu-OH	254.1 [M+Na$^+$]	12.277	1.8368

Figure S24. Chromatogram of *N*-Boc-Leu-Leu-OMe in DCM at 220 nm (entry 1, Table 2 in the article).

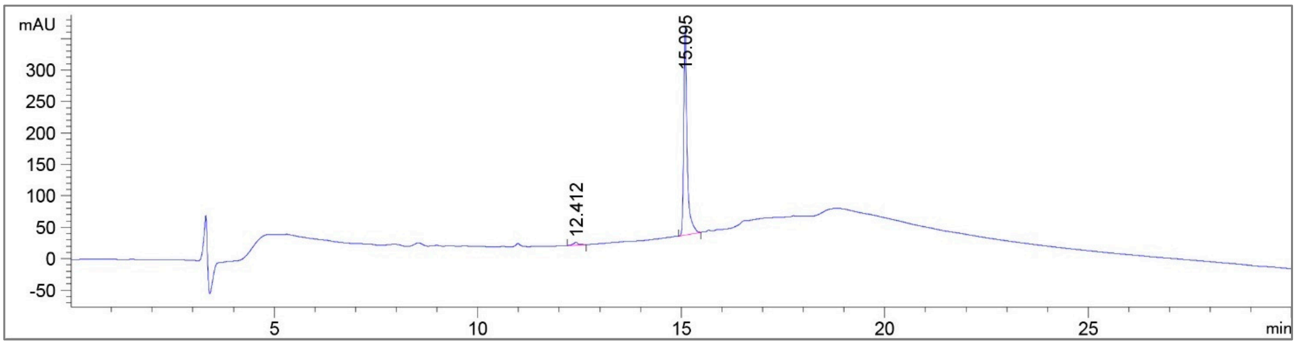

Product	m/z observed	Rt (min)	Area (%)
N-Boc-Phe-Leu-OMe	415.2 [M+Na$^+$]	15.095	98.1025
N-Boc-Phe-OH	288.0 [M+Na$^+$]	12.412	1.8975

Figure S25. Chromatogram of *N*-Boc-Phe-Leu-OMe in DCM at 220 nm (entry 2, Table 2 in the article).

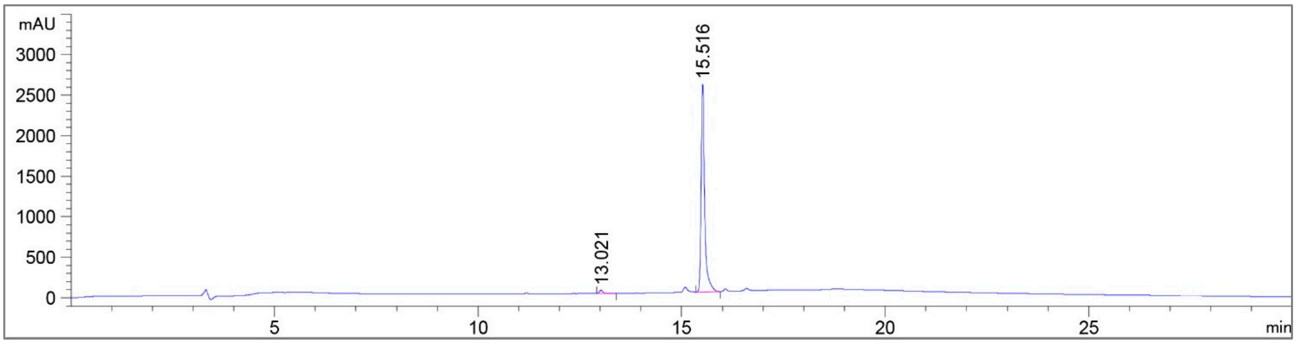

Product	m/z observed	Rt (min)	Area (%)
N-Boc-Arg(Pbf)-Leu-OMe	654.3	15.516	98.6870
N-Boc-Arg(Pbf)-OH	527.1	13.021	1.3130

Figure S26. Chromatogram of *N*-Boc-Arg(Pbf)-Leu-OMe in DCM at 220 nm (entry 3, Table 2 in the article).

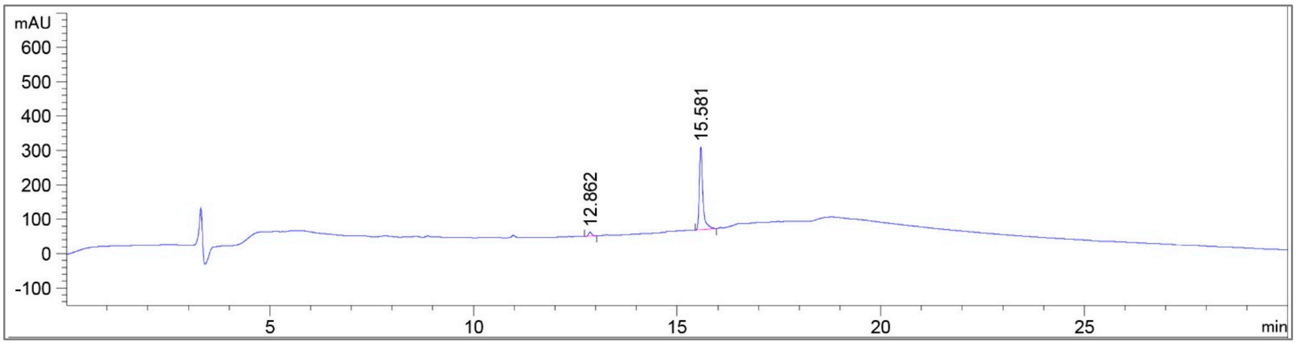

Product	m/z observed	Rt (min)	Area (%)
N-Boc-Asp(Bzl)-Leu-OMe	473.2 [M+Na$^+$]	15.581	95.7777
N-Boc-Asp(Bzl)-OH	346.1 [M+Na$^+$]	12.862	4.2223

Figure S27. Chromatogram of *N*-Boc-Asp(Bzl)-Leu-OMe in DCM at 220 nm (entry 4, Table 2 in the article).

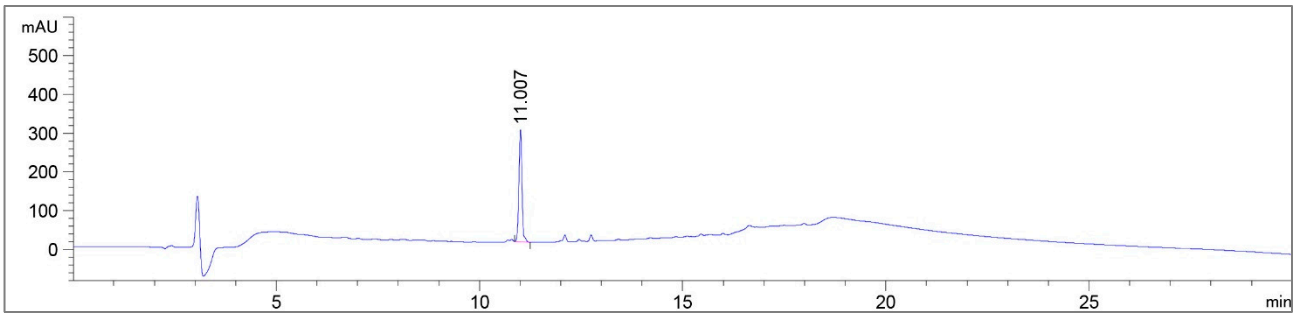

Product	m/z observed	Rt (min)
N-Boc-Ser-Leu-OMe	355.1 [M+Na$^+$]	11.007

Figure S28. Chromatogram of *N*-Boc-Ser-Leu-OMe in DCM at 220 nm (entry 5, Table 2 in the article).

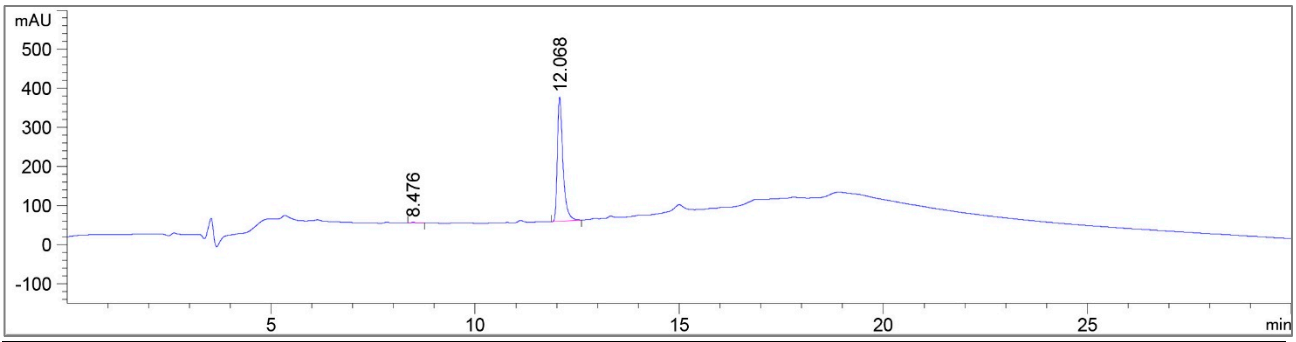

Product	m/z observed	Rt (min)	Area (%)
N-Boc-Thr-Leu-OMe	347.2	12.068	99.4711
N-Boc-Thr-OH	242.3 [M+Na$^+$]	8.476	0.5289

Figure S29. Chromatogram of *N*-Boc-Thr-Leu-OMe in DCM at 220 nm (entry 6, Table 2 in the article).

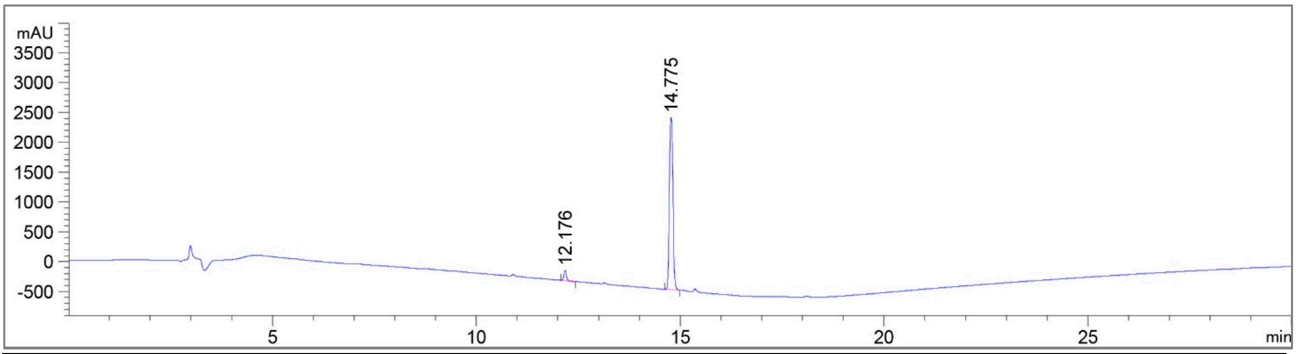

Product	m/z observed	Rt (min)	Area (%)
N-Boc-Trp-Leu-OMe	432.2	17.775	95.8005
N-Boc-Trp-OH	327.	12.176	4.1995

Figure S30. Chromatogram of *N*-Boc-Trp-Leu-OMe in DCM at 220 nm (entry 7, Table 2 in the article).

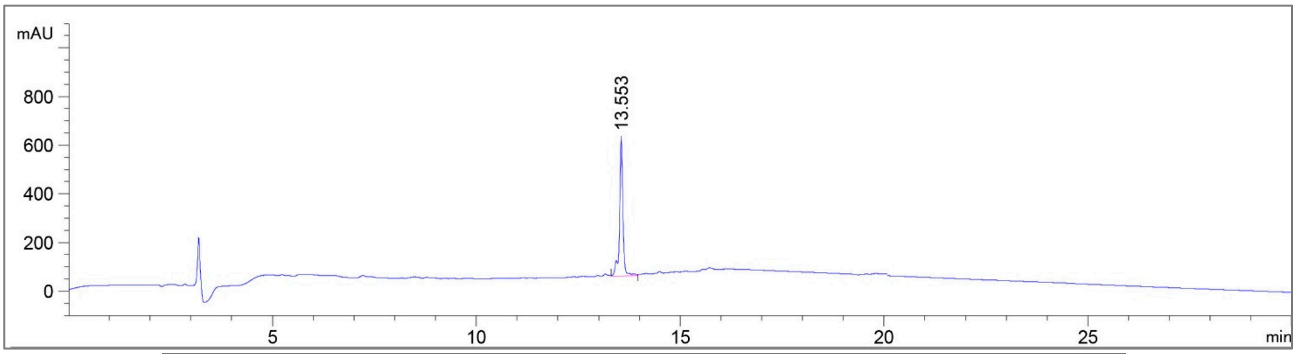

Product	m/z observed	Rt (min)
N-Boc-Aib-Leu-OMe	331.2	13.553

Figure S31. Chromatogram of *N*-Boc-Aib-Leu-OMe in DCM at 220 nm (entry 8, Table 2 in the article).

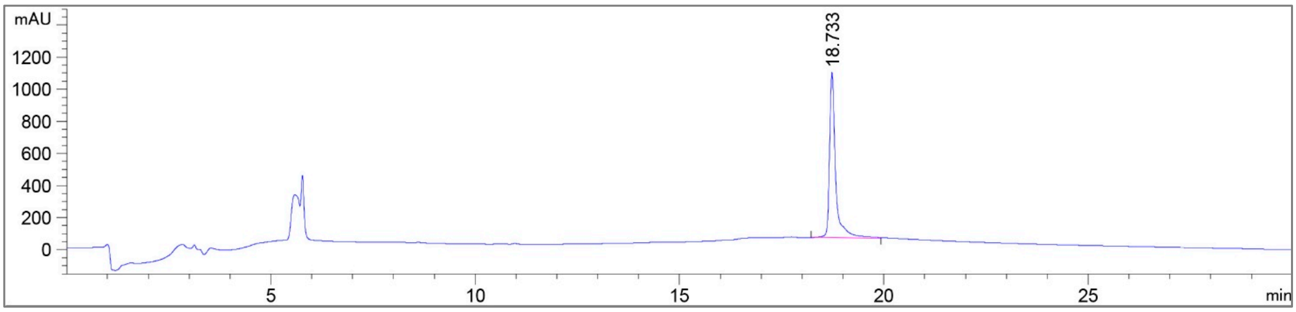

Product	m/z observed	Rt (min)	Area (%)
N-*N*-Fmoc-Leu-Leu-OBn	557.2	18.733	100.0000

Figure S32. Chromatogram of *N*-*N*-Fmoc-Leu-Leu-OBn in DCM at 220 nm (entry 9, Table 2 in the article).

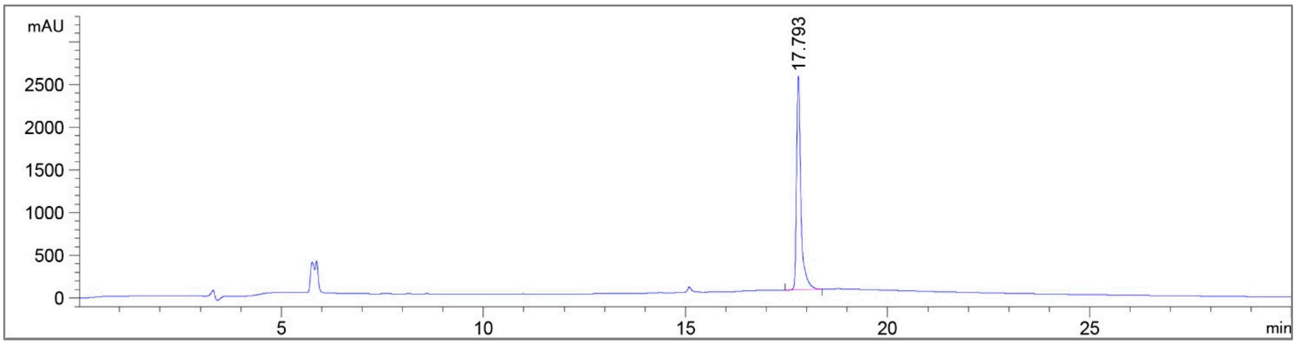

Product	m/z observed	Rt (min)
N-*N*-Fmoc-Trp-Leu-OBn	630.2	17.793

Figure S33. Chromatogram of *N*-Fmoc-Trp-Leu-OBn in DCM at 220 nm (entry 10, Table 2 in the article).

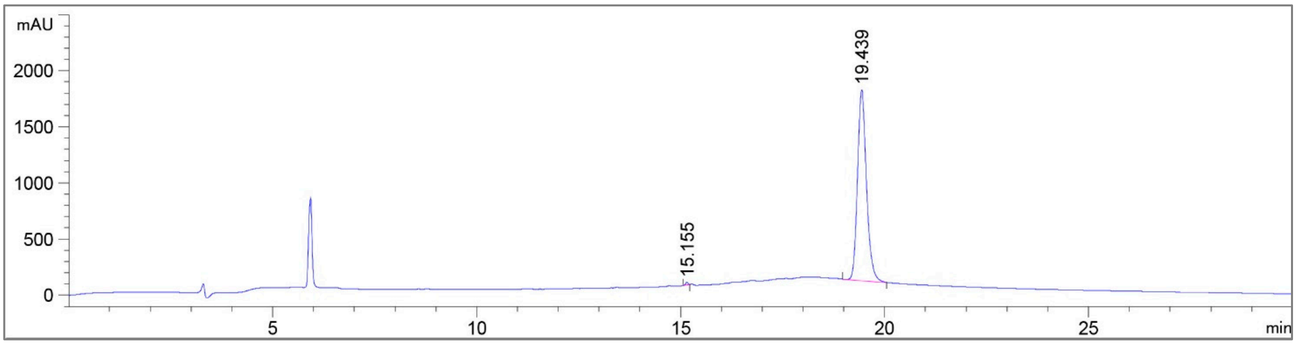

Product	m/z observed	Rt (min)	Area (%)
N-Fmoc-Arg(Pbf)-Leu-OBn	852.3	19.439	99.6125
N-Fmoc-Arg(Pbf)-OH	649.3	15.155	0.3875

Figure S34. Chromatogram of *N*-Fmoc-Arg(Pbf)-Leu-OBn in DCM at 220 nm (entry 11, Table 2 in the article).

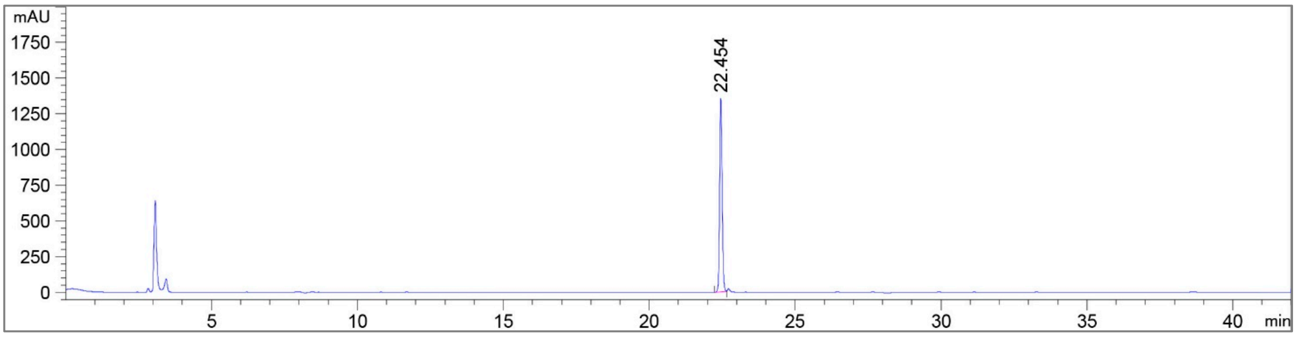

Product	m/z observed	Rt (min)
N-Fmoc-Asp(tBu)-Leu-OBn	637.3 [M+Na$^+$]	22.454

Figure S35. Chromatogram of *N*-Fmoc-Asp(tBu)-Leu-OBn in DCM at 220 nm (entry 12, Table 2 in the article). The peak at 3 min is associated with p-Toluenesulfonic acid which is salified with H_2N-Leu-OBn.

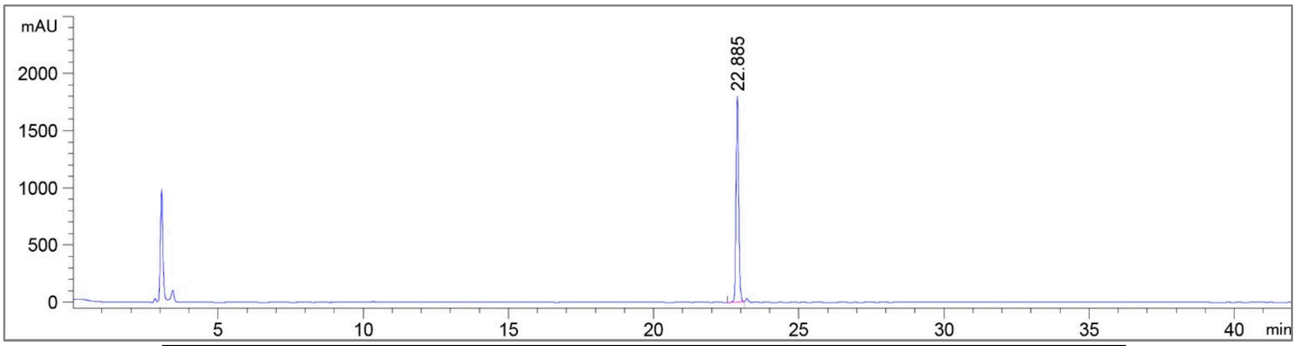

Product	m/z observed	Rt (min)
N-Fmoc-Ser(tBu)-Leu-OBn	587.3	22.885

Figure S36. Chromatogram of *N*-Fmoc-Ser(tBu)-Leu-OBn in DCM at 220 nm (entry 13, Table 2 in the article). The peak at 3 min is associated with p-Toluenesulfonic acid which is salified with H_2N-Leu-OBn.

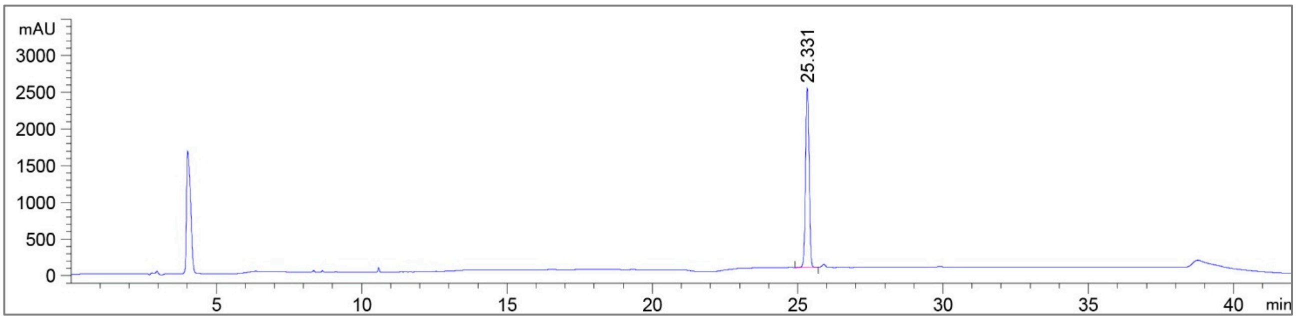

Product	m/z observed	Rt (min)
N-Fmoc-Thr(tBu)-Leu-OBn	623.3 [M+Na$^+$]	17.384

Figure S37. Chromatogram of *N*-Fmoc-Thr(tBu)-Leu-OBn in DCM at 220 nm (entry 14, Table 2 in the article). The peak at 3 min is associated with p-Toluenesulfonic acid which is salified with H_2N-Leu-OBn.

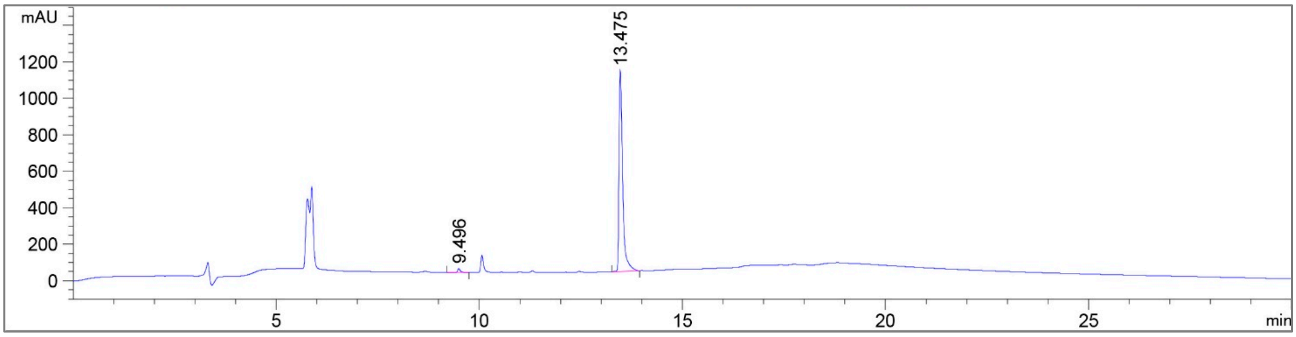

Product	m/z observed	Rt (min)	Area (%)
N-Fmoc-Arg-Leu-OBn	600.3	13.475	98.2780
H_2*N*-Leu-OBn	222.1	9.496	1.7220

Figure S38. Chromatogram of *N*-Fmoc-Arg-Leu-OBn in DCM at 220 nm (entry 15, Table 2 in the article).

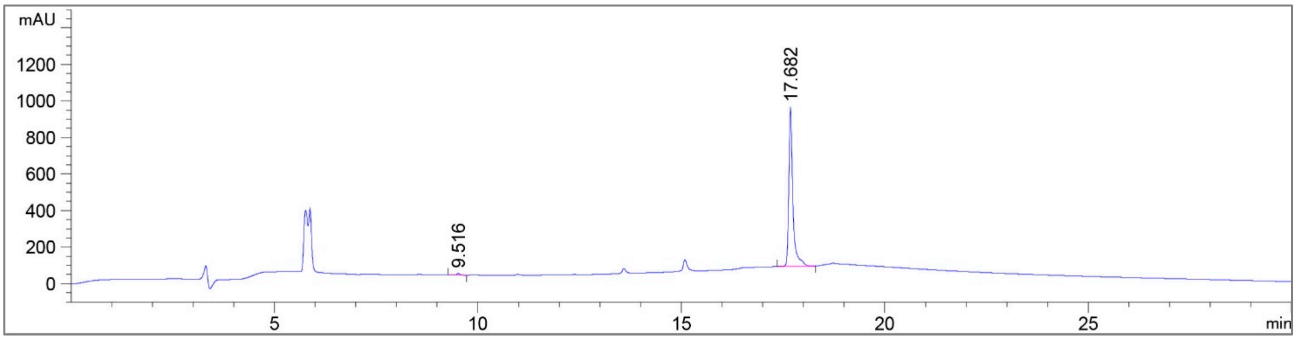

Product	m/z observed	Rt (min)	Area (%)
N-Fmoc-Aib-Leu-OBn	529.2	17.682	99.0814
H_2*N*-Leu-OBn	222.1	9.516	0.9186

Figure S39. Chromatogram of *N*-Fmoc-Aib-Leu-OBn in DCM at 220 nm (entry 16, Table 2 in the article).

4. Racemization evaluation

N-Boc-L/D-Phg-Leu-OMe was synthetized as described in the chapter "Materials and Methods" in the main paper. Each diastereomer and their mixture were injected into HPLC using chiral column working at 1 ml/min with Hexane and iPrOH (90:10).

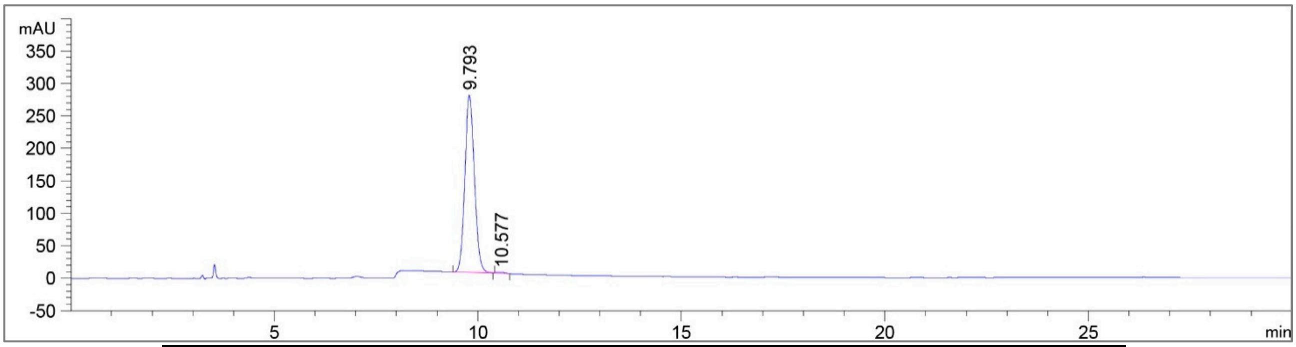

Product	Rt (min)	Area (%)
N-Boc-L-Phg-Leu-OMe	9.793	99.547
N-Boc-D-Phg-Leu-OMe	10.577	0.453

Figure S40. Chromatogram of *N*-Boc-L-Phg-Leu-OMe in DCM at 220 nm.

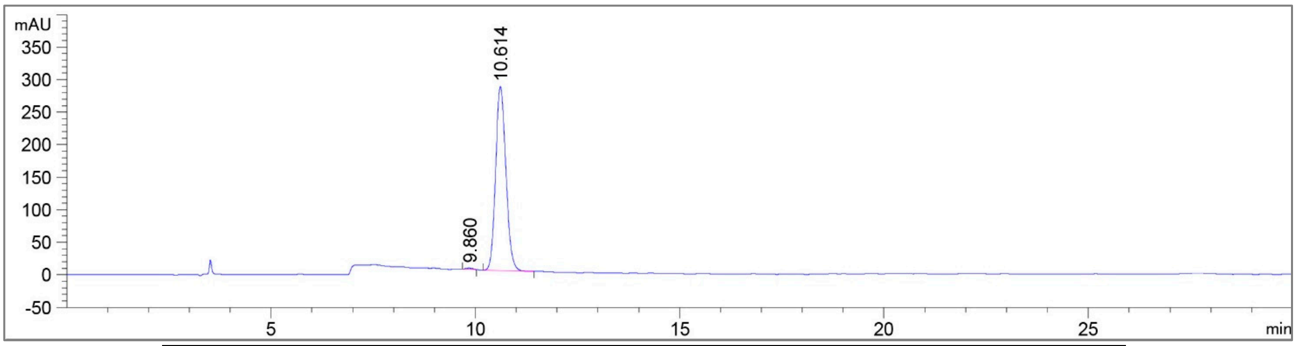

Product	Rt (min)	Area (%)
N-Boc-D-Phg-Leu-OMe	10.614	99.448
N-Boc-L-Phg-Leu-OMe	9.860	0.552

Figure S41. Chromatogram of *N*-Boc-D-Phg-Leu-OMe in DCM at 220 nm.

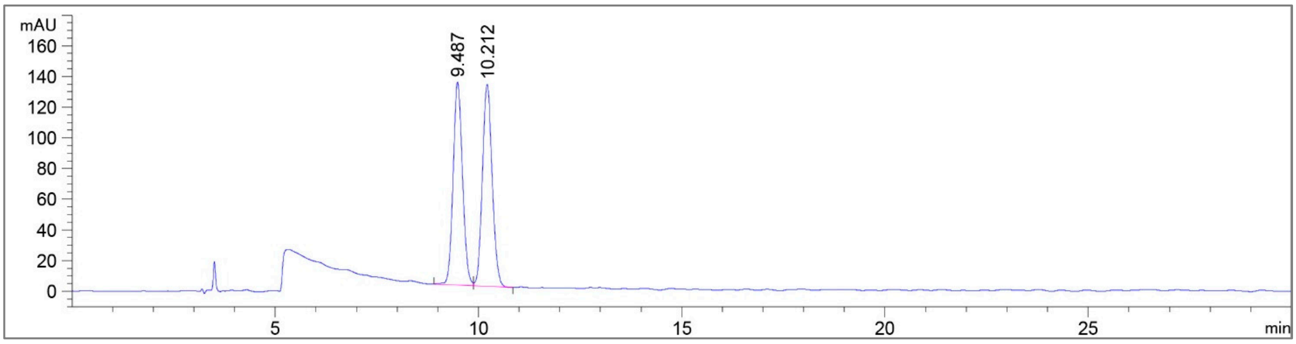

Product	Rt (min)	Area (%)
N-Boc-L-Phg-Leu-OMe	9.487	49.148
N-Boc-D-Phg-Leu-OMe	10.212	50.852

Figure S42. Chromatogram of a mixture between *N*-Boc-L-Phg-Leu-OMe and *N*-Boc-D-Phg-Leu-OMe at 220 nm.

5. HPLC chromatogram: SolPPS of Leu-Enkephalin precursor *via N*-Boc chemistry

Intermediate isolation

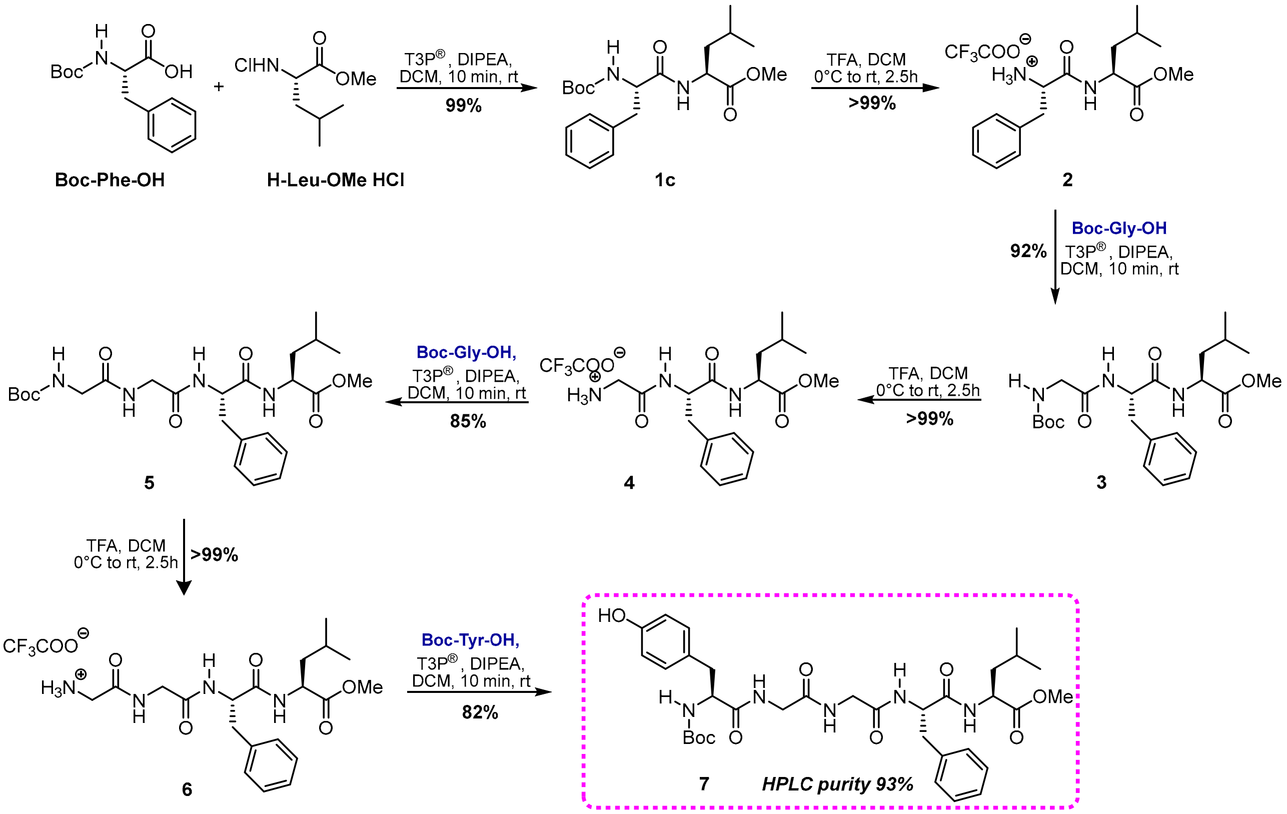

Scheme S2. SolPPS of N-Boc-Leu-Enkephalin-OMe **7** (reported yields refer to crude reactions).

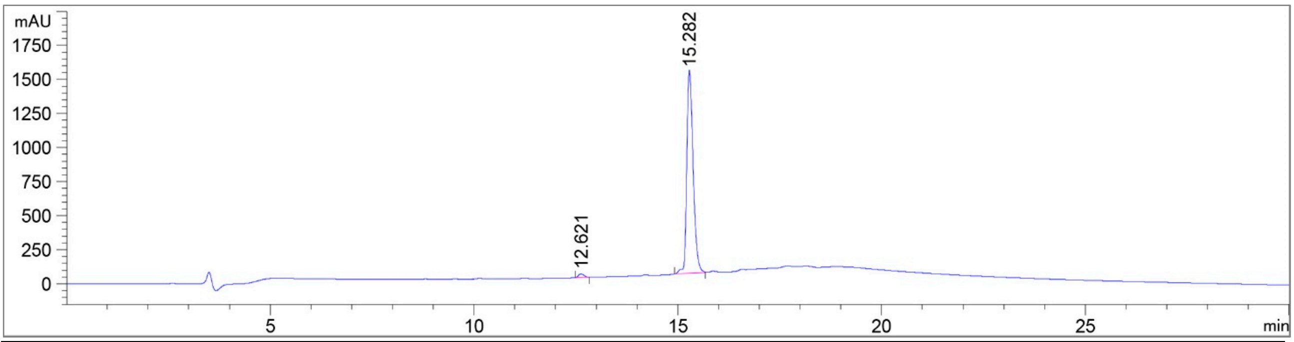

Product	m/z observed	Rt (min)	Area (%)
N-Boc-Phe-Leu-OMe (**1c**)	392.9	15.282	97.4034
N-Boc-Phe-OH	288.0	12.621	2.5966

Figure S43. Chromatogram of intermediate *N*-Boc-Phe-Leu-OMe (**1c**) in the synthesis of Leu-Enkephalin precursor.

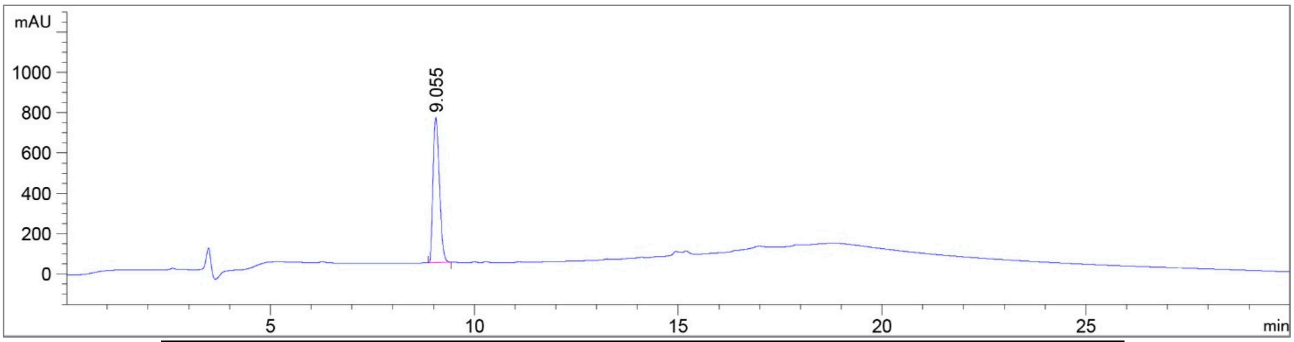

Product	m/z observed	Rt (min)
TFA·H_2N-Phe-Leu-OMe (**2**)	293.0	9.055

Figure S44. Chromatogram of intermediate TFA·H_2N-Phe-Leu-OMe (**2**) in the synthesis of Leu-Enkephalin precursor.

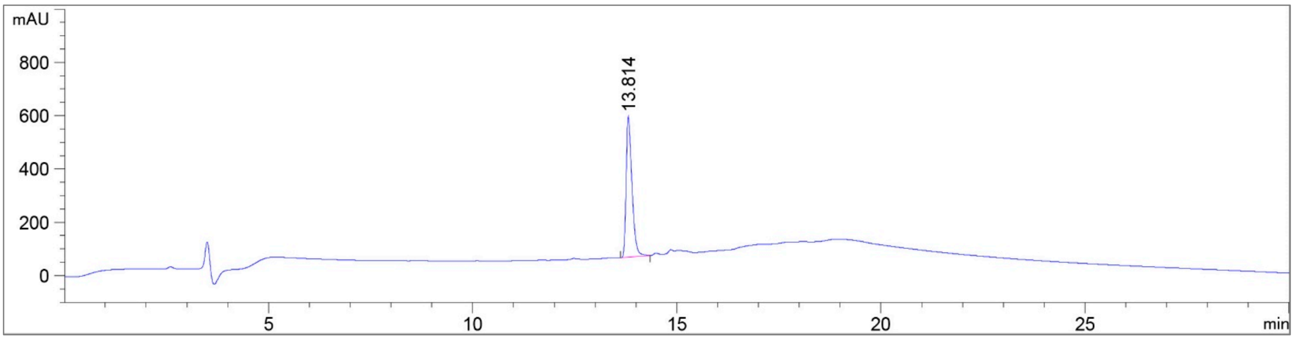

Product	m/z observed	Rt (min)
N-Boc-Gly-Phe-Leu-OMe (**3**)	450.2	13.814

Figure S45. Chromatogram of intermediate *N*-Boc-Gly-Phe-Leu-OMe (**3**) in the synthesis of Leu-Enkephalin precursor.

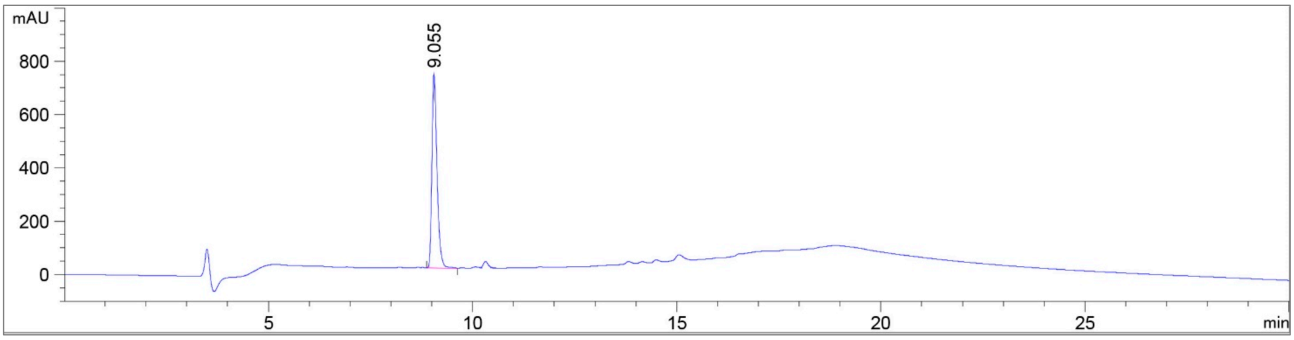

Product	m/z observed	Rt (min)
TFA·H_2N-Gly-Phe-Leu-OMe (**4**)	350.2	9.055

Figure S46. Chromatogram of intermediate TFA·H_2N-Gly-Phe-Leu-OMe (**4**) in the synthesis of Leu-Enkephalin precursor.

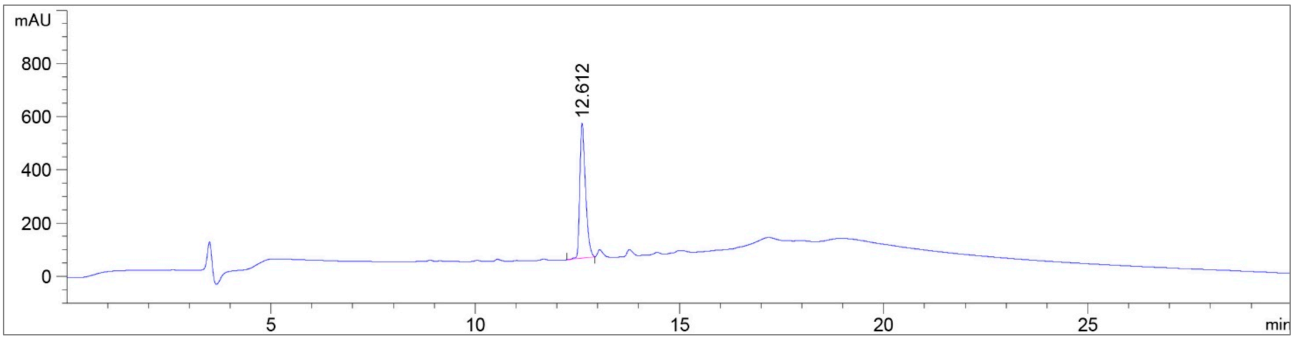

Product	m/z observed	Rt (min)
N-Boc-Gly-Gly-Phe-Leu-OMe (**5**)	507.2	12.612

Figure S47. Chromatogram of intermediate *N*-Boc-Gly-Gly-Phe-Leu-OMe (**5**) in the synthesis of Leu-Enkephalin precursor.

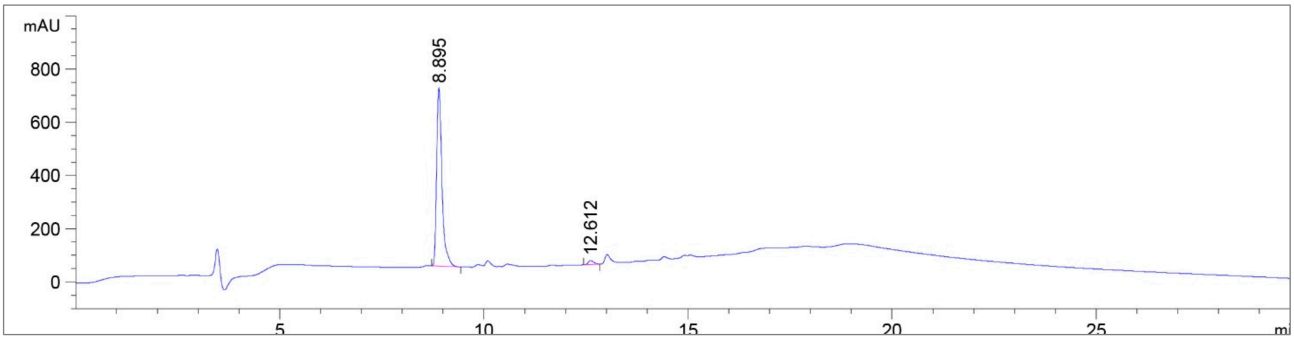

Product	m/z observed	Rt (min)	Area (%)
TFA·H_2N-Gly-Gly-Phe-Leu-OMe (**6**)	407.2	8.895	97.8118
N-Boc-Gly-Gly-Phe-Leu-OMe (**5**)	529.3 [M+Na^+]	12.612	2.1882

Figure S48. Chromatogram of intermediate TFA·H_2N-Gly-Gly-Phe-Leu-OMe (**6**) in the synthesis of Leu-Enkephalin precursor.

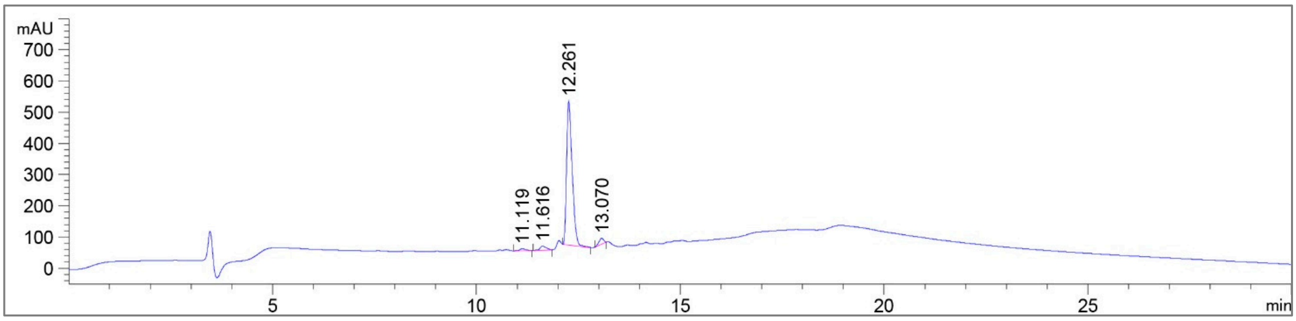

Product	m/z observed	Rt (min)	Area (%)
N-Boc-Tyr-**Gly-Gly**-Gly-Gly-Phe-Leu-OMe	784.4	11.119	1.2893
N-Boc-Tyr-**Gly-**Gly-Gly-Phe-Leu-OMe	727.3	11.616	2.8385
N-Boc-Tyr-Gly-Gly-Phe-Leu-OMe (**7**)	670.3	12.261	92.8154
N-Boc-Tyr-**Gly**-Phe-Leu-OMe	613.3	13.070	3.0568

Figure S49. Chromatogram of Leu-Enkephalin precursor (**7**).

Continuous protocol

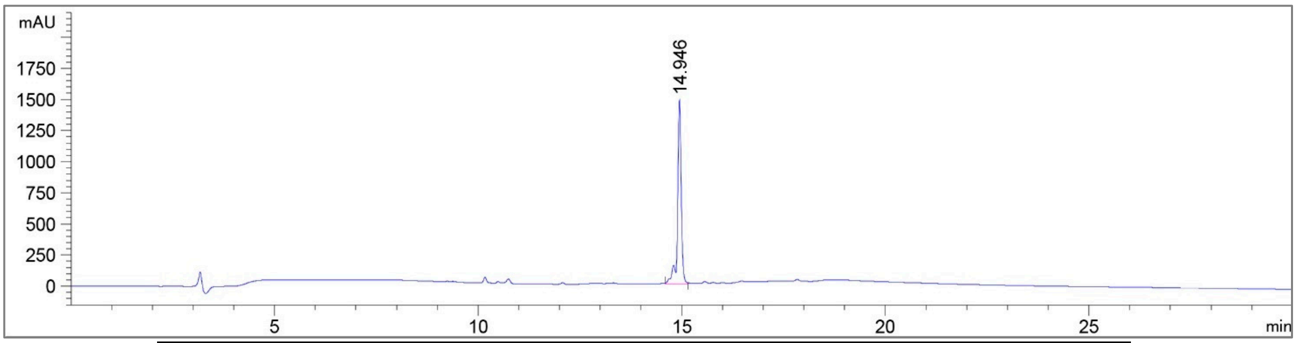

Product	m/z observed	Rt (min)
N-Boc-Phe-Leu-Ome (**1c**)	293.1	14.946

Figure S50. Chromatogram of intermediate *N*-Boc-Phe-Leu-OMe (**1c**) in the synthesis of Leu-Enkephalin precursor.

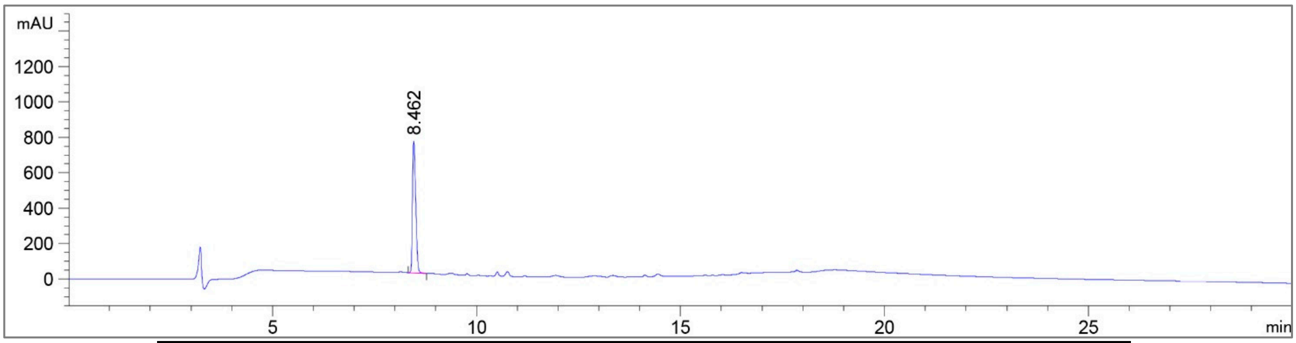

Product	m/z observed	Rt (min)
H_2N-Phe-Leu-Ome (**2**)	293.2	8.462

Figure S51. Chromatogram of intermediate H_2N-Phe-Leu-Ome (**2**) in the synthesis of Leu-Enkephalin precursor.

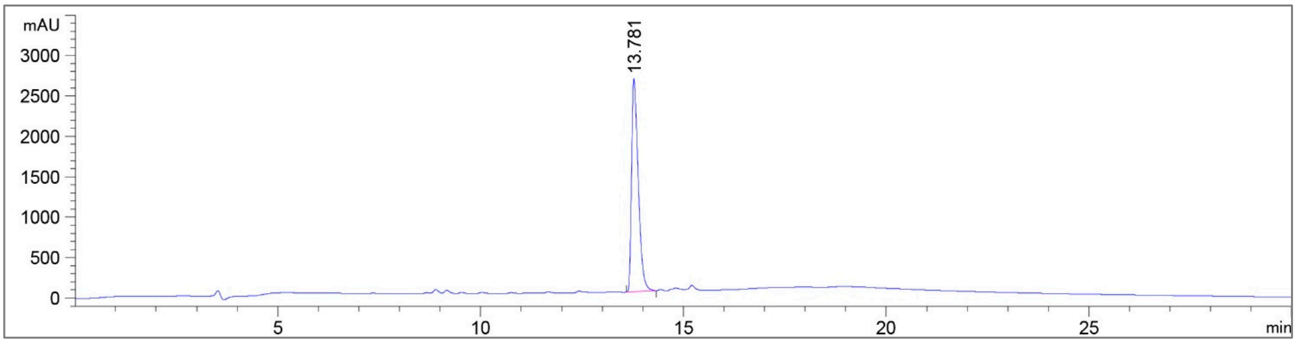

Product	m/z observed	Rt (min)
N-Boc-Gly-Phe-Leu-OMe (**3**)	450.0	13.781

Figure S52. Chromatogram of intermediate *N*-Boc-Gly-Phe-Leu-OMe (**3**) in the synthesis of Leu-Enkephalin precursor.

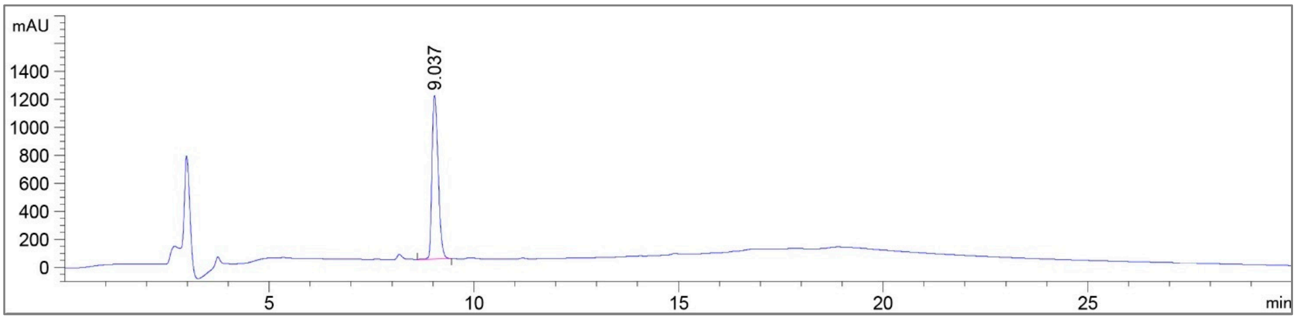

Product	m/z observed	Rt (min)
H_2N-Gly-Phe-Leu-OMe (**4**)	350.2	9.037

Figure S53. Chromatogram of intermediate H_2N-Gly-Phe-Leu-OMe (**4**) in the synthesis of Leu-Enkephalin precursor.

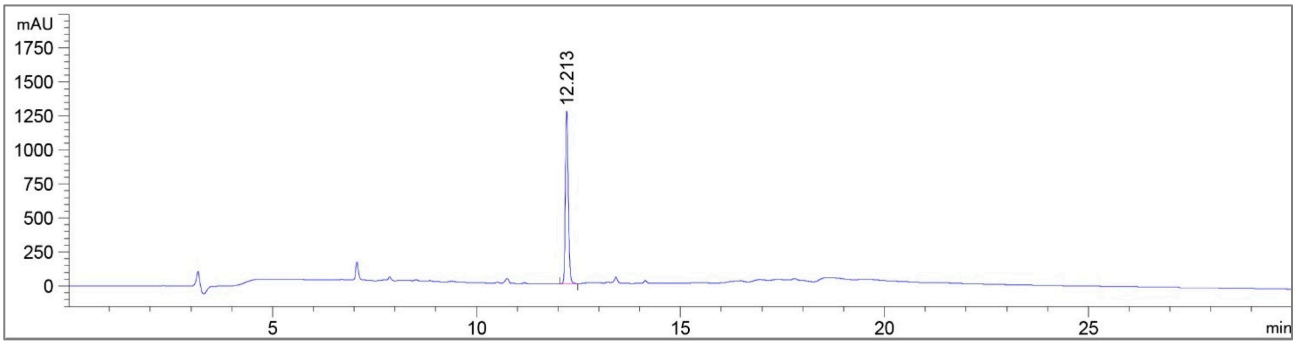

Product	m/z observed	Rt (min)
N-Boc-Gly-Gly-Phe-Leu-OMe (**5**)	507.2	12.213

Figure S54. Chromatogram of intermediate *N*-Boc-Gly-Gly-Phe-Leu-OMe (**5**) in the synthesis of Leu-Enkephalin precursor.

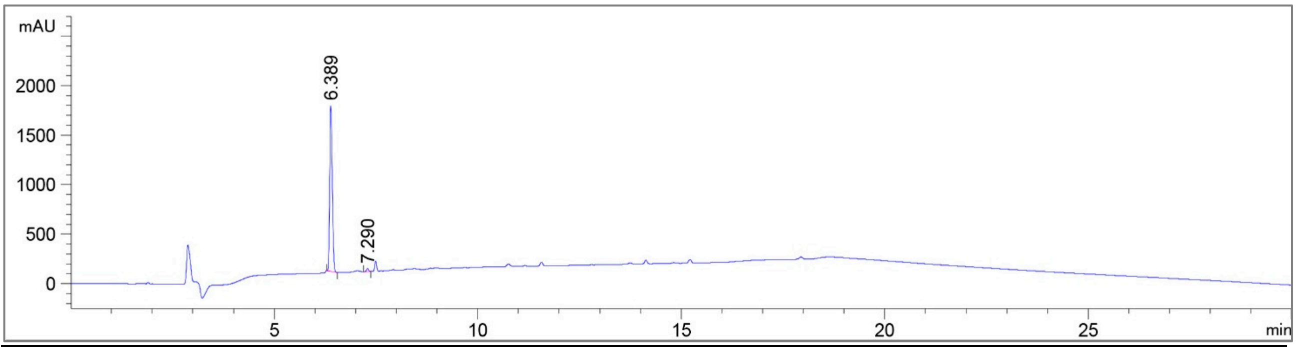

Product	m/z observed	Rt (min)	Area (%)
H_2N-Gly-Gly-Phe-Leu-OMe (**6**)	407.2	6.389	98.765
H_2N-**Gly-Gly**-Gly-Gly-Phe-Leu-OMe	520.3	7.290	1.235

Figure S55. Chromatogram of intermediate H_2N-Gly-Gly-Phe-Leu-OMe (**6**) in the synthesis of Leu-Enkephalin precursor.

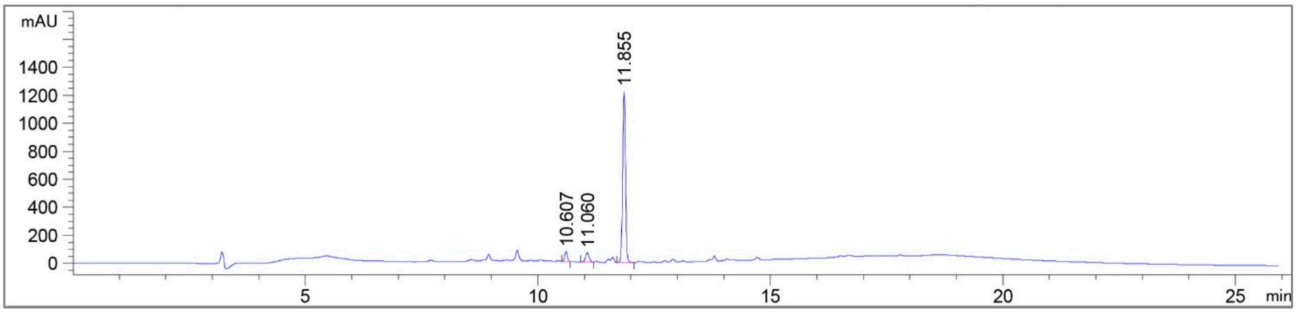

Product	m/z observed	Rt (min)	Area (%)	
N-Boc-Tyr-**Gly-Gly**-Gly-Gly-Phe-Leu-OMe	784.3	10.607	4.8128	
N-Boc-Tyr(OT3P)-Gly-Gly-Phe-Leu-OMe (**7**)*	988.3	11.060	6.0560	} 95
N-Boc-Tyr-Gly-Gly-Phe-Leu-OMe (**7**)	670.3	11.855	89.1312	

* Phosphorylated intermediate spontaneously hydrolysed to compound **7** after acidic work-up.

Figure S56. Chromatogram of Leu-Enkephalin precursor (**7**).

6. HPLC Chromatograms: Full LPPS of Leu-Enkephalin via *N*-Fmoc chemistry

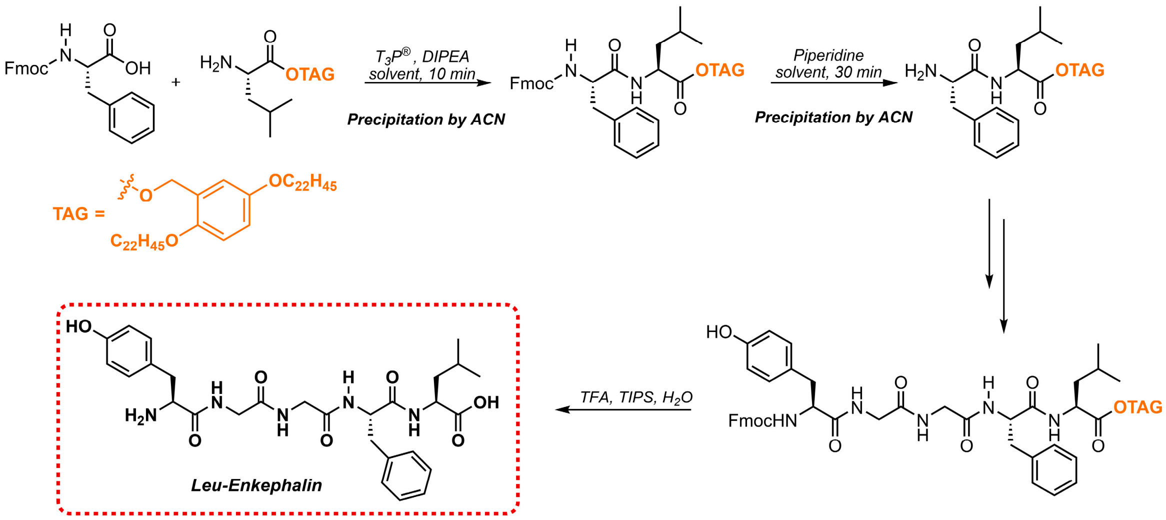

Scheme S3. LPPS of Leu-Enkephalin using polycarbon tag.

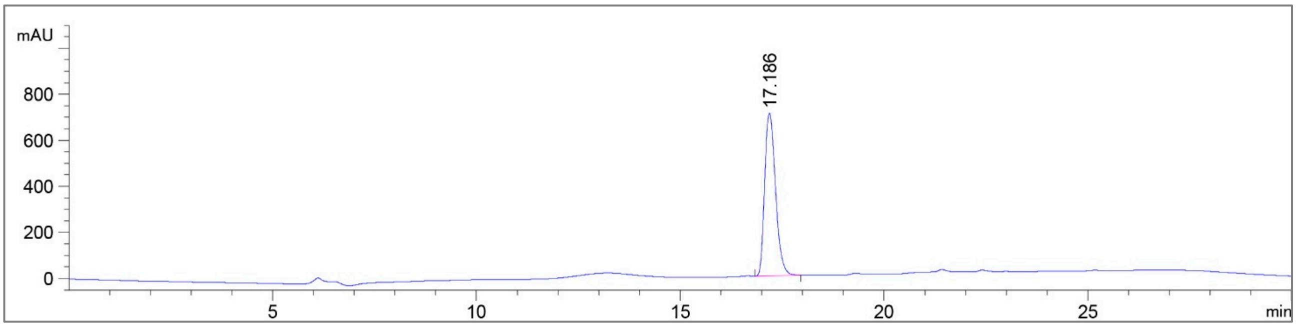

Product	m/z observed	Rt (min)
H_2N-Tyr-Gly-Gly-Phe-Leu-OH	556.2	17.186

Figure S57. Chromatogram of Leu-Enkephalin in DCM precipitating after each step during the synthesis.

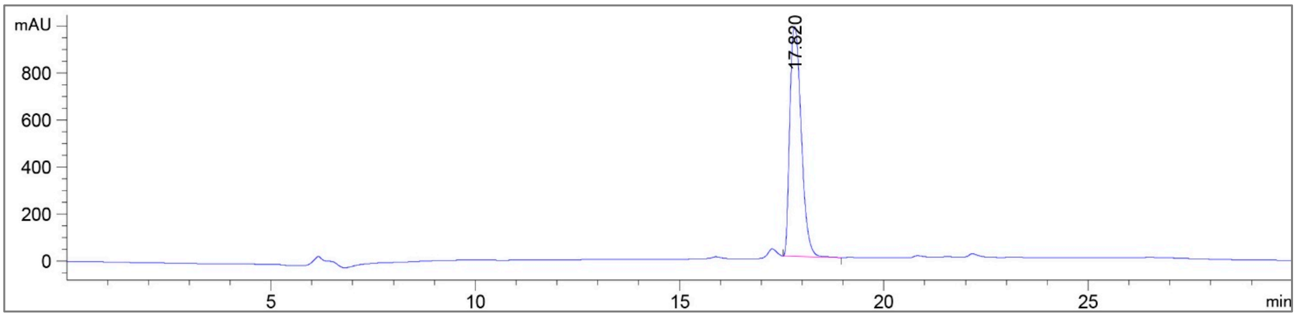

Product	m/z observed	Rt (min)
H_2N-Tyr-Gly-Gly-Phe-Leu-OH	556.2	17.820

Figure S58. Chromatogram of Leu-Enkephalin in DCM precipitating after deprotection during the synthesis.

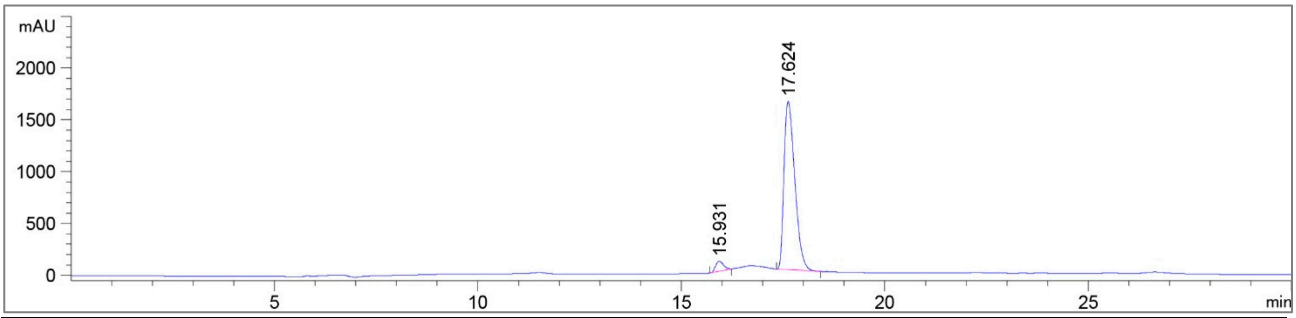

Product	m/z observed	Rt (min)	Area (%)
H_2N-Tyr-Gly-Gly-Phe-Leu-OH	556.2	17.624	95.8625
H_2N-Gly-Gly-Phe-Leu-OH (Des-Tyr)	393.1	15.931	4.1375

Figure S59. Chromatogram of Leu-Enkephalin in Anisole precipitating after each step during the synthesis.

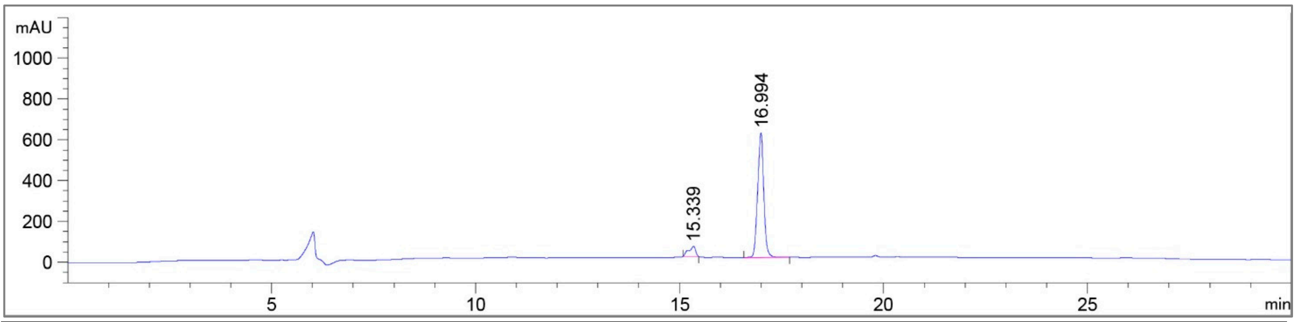

Product	m/z observed	Rt (min)	Area (%)
H_2N-Tyr-Gly-Gly-Phe-Leu-OH	556.2	16.994	91.3749
H_2N-Gly-Gly-Phe-Leu-OH (Des-Tyr)	393.1	15.339	8.6251

Figure S60. Chromatogram of Leu-Enkephalin in Anisole precipitating after deprotection during the synthesis. The peak around 6 min is related to TFA added directly into the vial.

914 Hslp huv ri Ohx0Hqnhskddlq v|qwkhwl}hg e| VSSV

To verify the stereochemical purity of the synthesized Leu-Enkephalin all the epimers as reference were synthesized through SPPS in DMF.

Manual syntheses of Leu-Enkephalin epimers were carried out at room temperature in glass syringes fitted with a polyethylene porous disc and connected to a vacuum source to remove excess reagents and solvents. The syntheses were carried out by using Wang-PS resin (200 mg, loading 1.2 mmol g−1). After swelling of the resin in 2 mL of DMF, a solution of *N*-Fmoc-Leu-OH (or *N*-(D)-Fmoc-Leu-OH; 2.5 eq. respect to the loading of the resin), DIC (1.25 eq.) and DMAP (0.1 eq.) in DMF, preactivated for 5 min, was charged onto the resin and stirred for 1 h and the resin was washed with DMF (3x2 mL). The Fmoc protective group was removed by 20% piperidine in DMF (2 × 2 mL, 15 min each) and the resin was washed with DMF (3 × 2 mL). N-Fmoc-Phe-OH (or *N*-Fmoc-(D)-Phe-OH), *N*-Fmoc-Gly-OH and *N*-Fmoc-Tyr(tBu)-OH (or *N*-Fmoc-(D)-Tyr(tBu)-OH; 2 eq.) were diluted in DMF (2, 5 mL), pre-activated by DIC and OxymaPure® (3 eq.) for 3 min and coupled to the resin in 60 min. After each cou-pling step, the Fmoc protective group was removed by treating the peptide resin with a 20% piperidine solution in DMF (2 × 2 mL, 15 min each) and the resin was washed with DMF (3 × 2 mL). After the Fmoc-cleavage of the N-terminal alpha-amino group, the peptide resin was washed with DMF (3 × 2 mL) and DCM (3 × 2 mL). The dry peptide resin was suspended in 5 mL of the mixture TFA/TIS/H_2O (95/2.5/2.5 v/v/v) and stirred for 2 h. The resin was filtered off and diisopropylether (20 mL) cooled to 4 °C was added to the solution. The peptide was filtered and dried in vacuo to obtain crude epimers of Leu-Enkephalin. The relative chromatograms are reported in Fig. S61–S64.

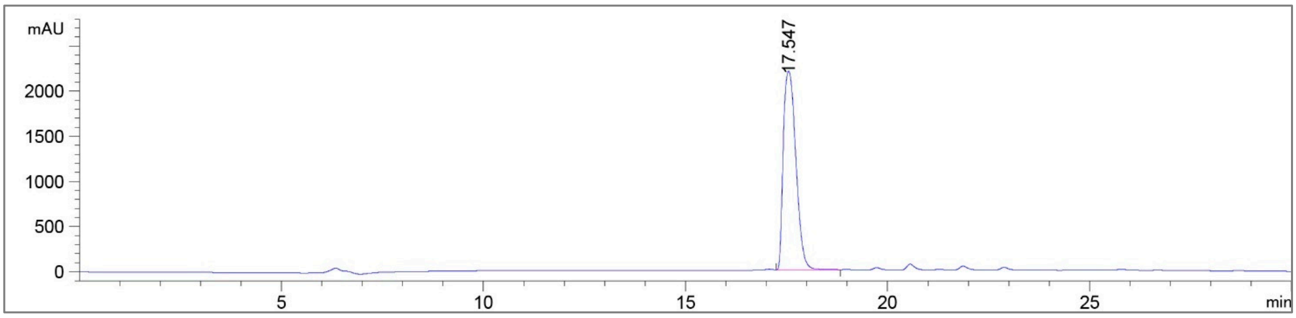

Figure S61. Chromatogram of Leu-Enkephalin synthetized by SPPS.

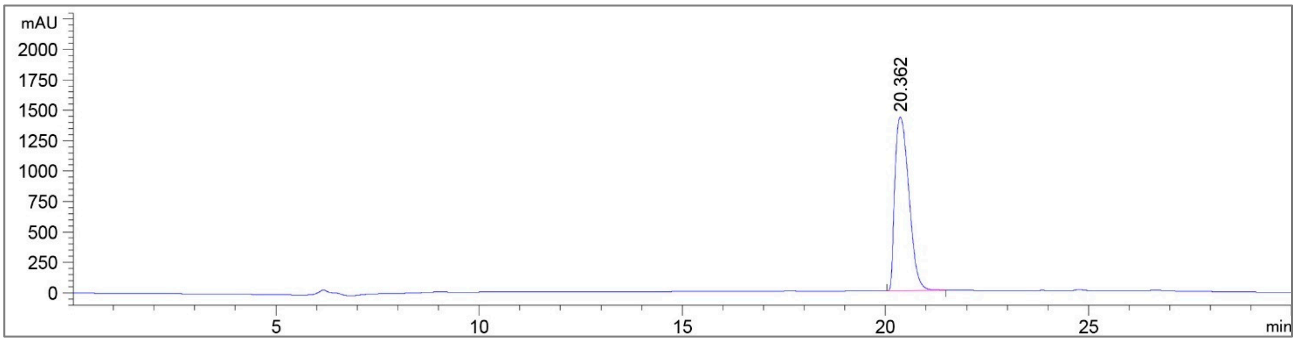

Figure S62. Chromatogram of Leu-Enkephalin epimer with D-Leu synthetized by SPPS.

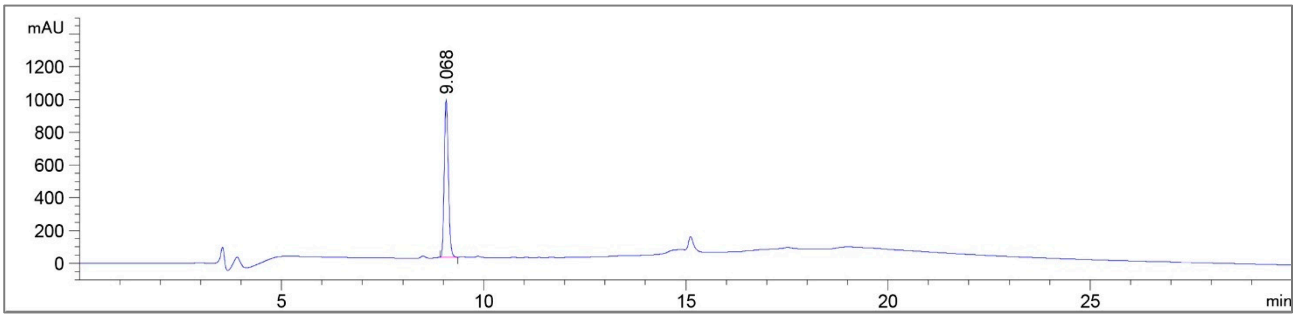

Figure S63. Chromatogram of Leu-Enkephalin epimer with D-Phe synthetized by SPPS.

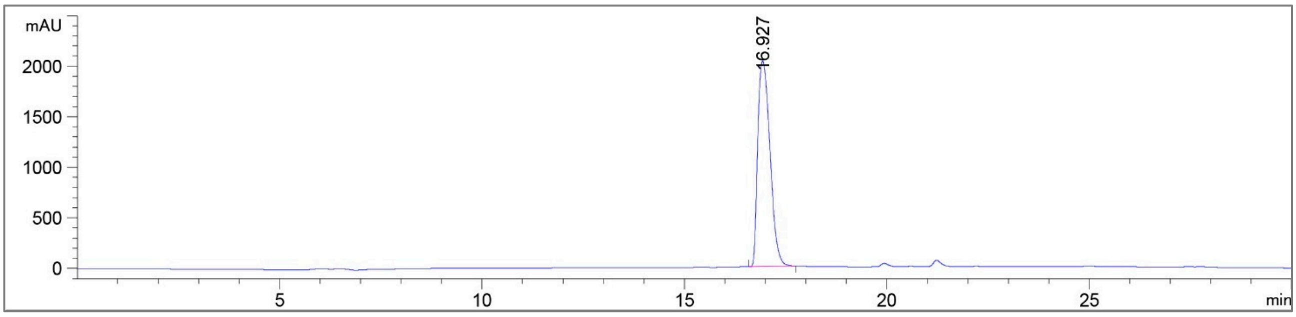

Figure S64. Chromatogram of Leu-Enkephalin epimer with D-Tyr synthetized by SPPS.

7. HPLC Chromatograms: Solvents and amino acids

Solvents

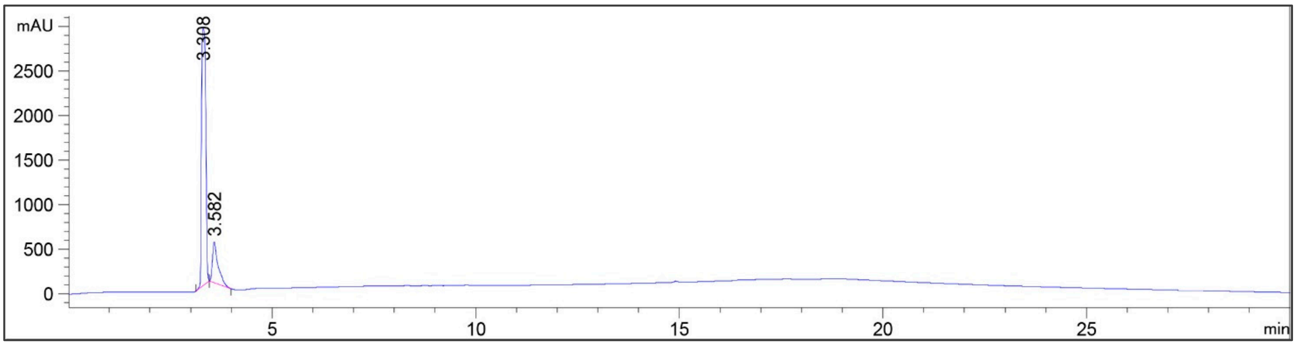

Figure S65. Chromatogram of DMF.

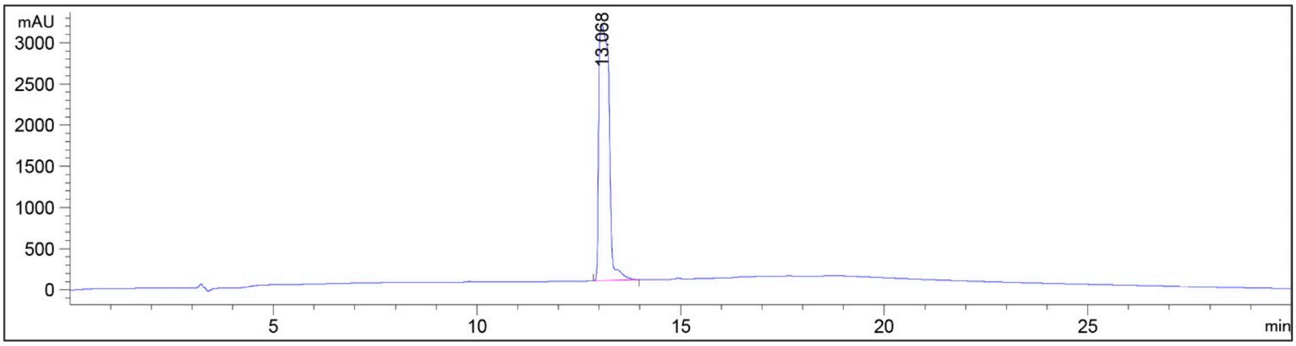

Figure S66. Chromatogram of Anisole.

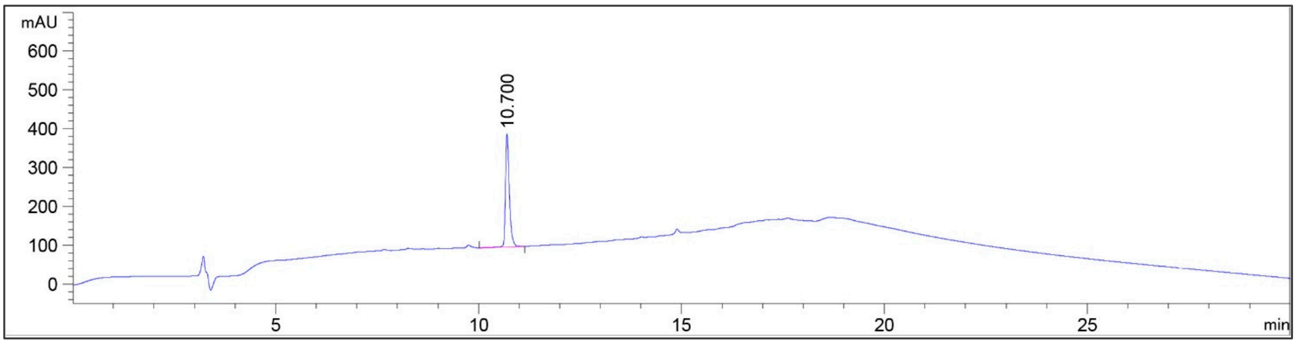

Figure S67. Chromatogram of PrOAc.

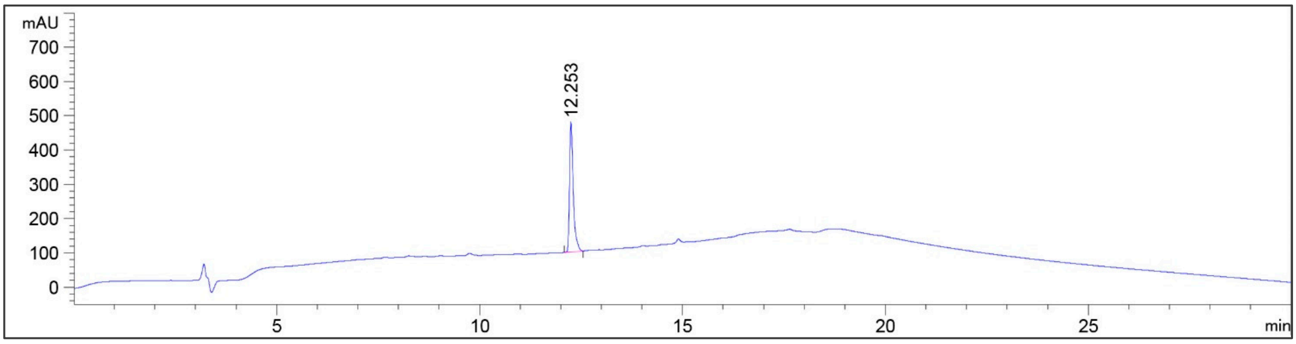

Figure S68. Chromatogram of tBuOAc.

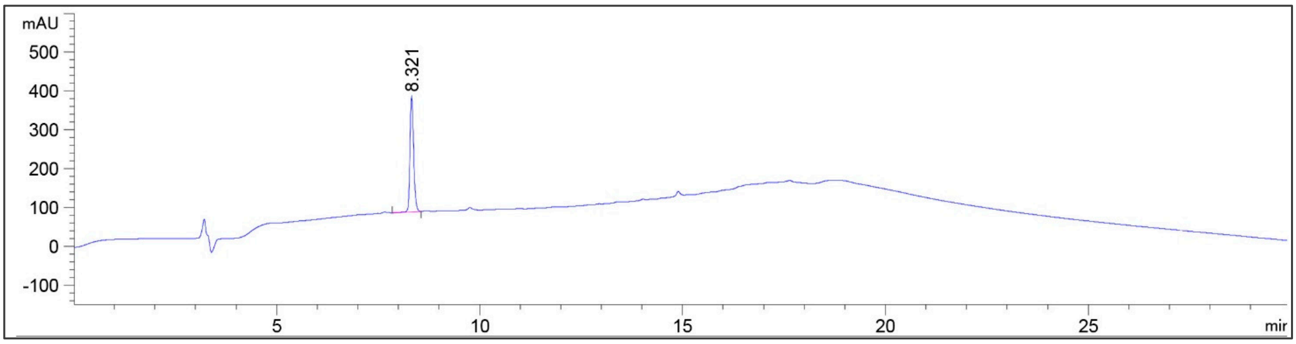

Figure S69. Chromatogram of EtOAc.

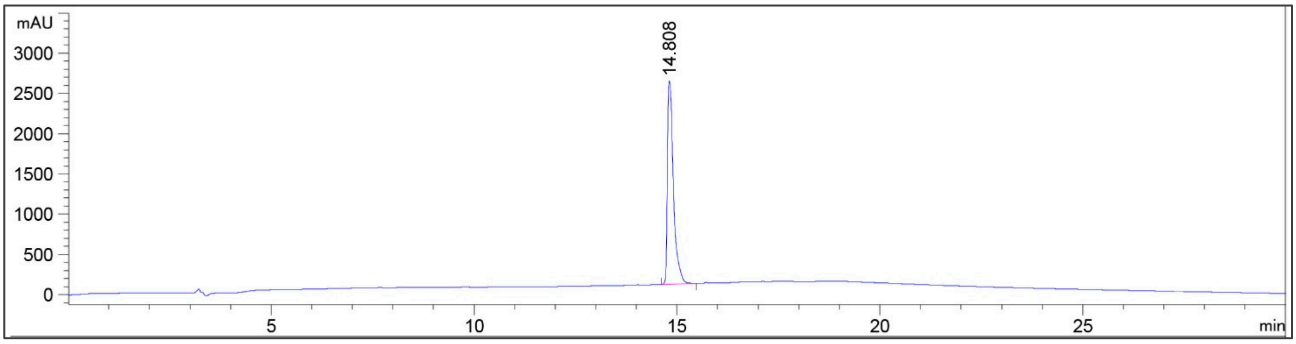

Figure S70. Chromatogram of NOP.

Amino acids

Amino acids were injected into HPLC by analytical method A, described in Chapter 1 of this file. Only in the cases of *N*-Fmoc-Thr(tBu)-OH, *N*-Fmoc-Ser(tBu)-OH and *N*-Fmoc-Asp(tBu)-OH, it was used method C because the respective dipeptides did not elute with the former method.

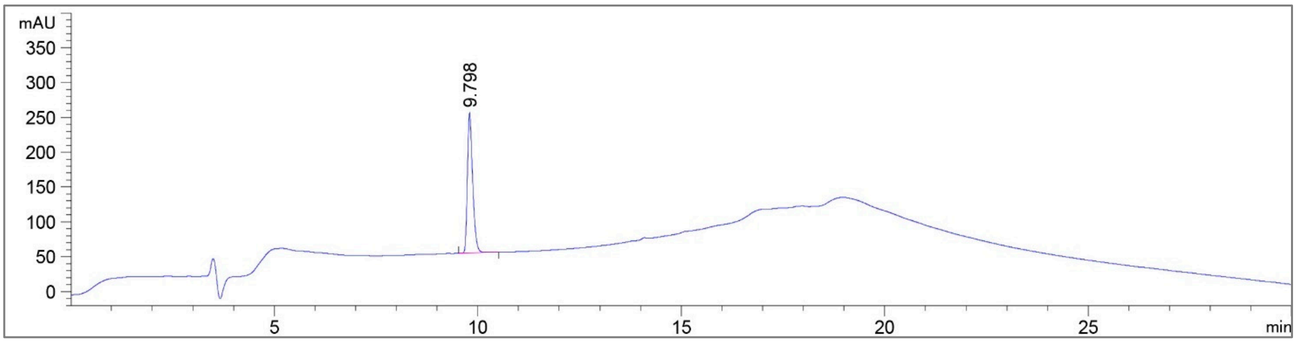

Figure S71. Chromatogram of *N*-Boc-Aib-OH.

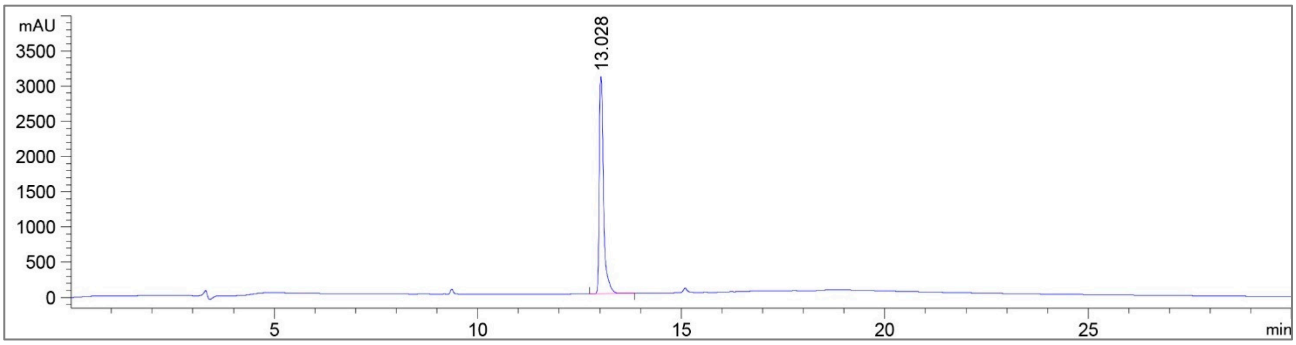

Figure S72. Chromatogram of *N*-Boc-Arg(PBF)-OH.

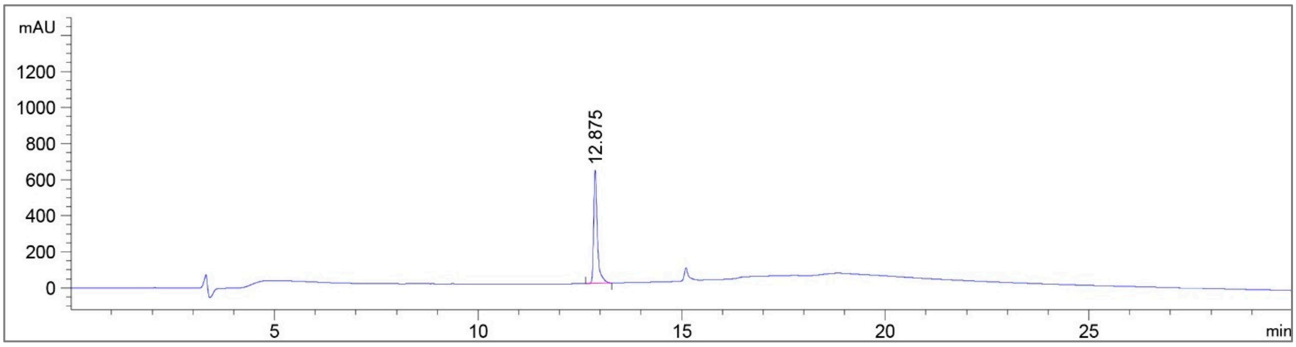

Figure S73. Chromatogram of *N*-Boc-Asp(Bzl)-OH.

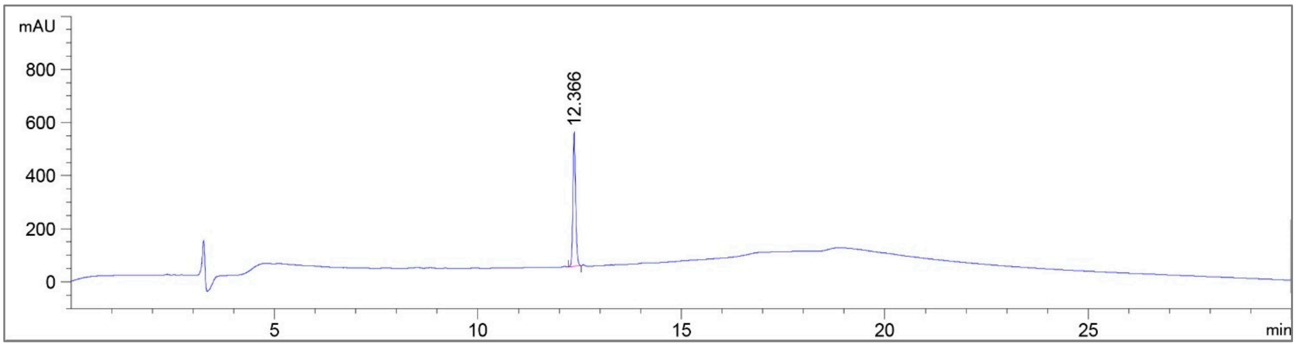

Figure S74. Chromatogram of *N*-Boc-Leu-OH.

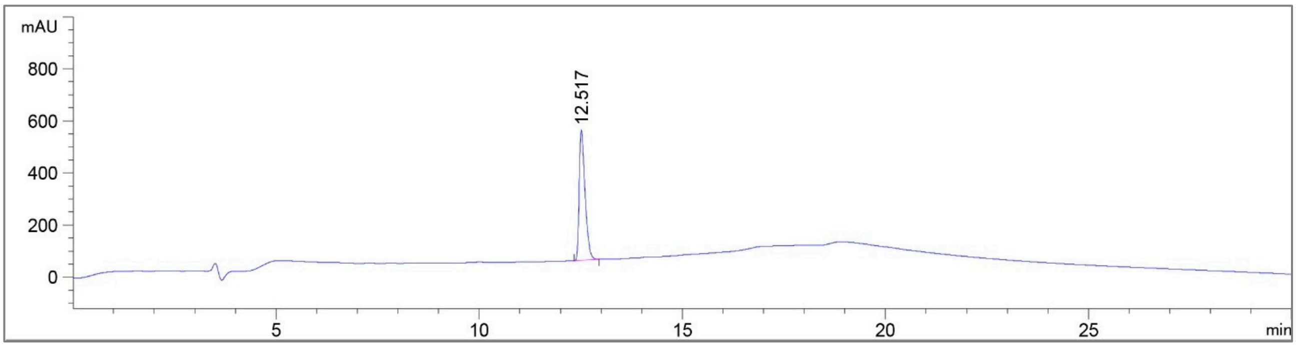

Figure S75. Chromatogram of *N*-Boc-Phe-OH.

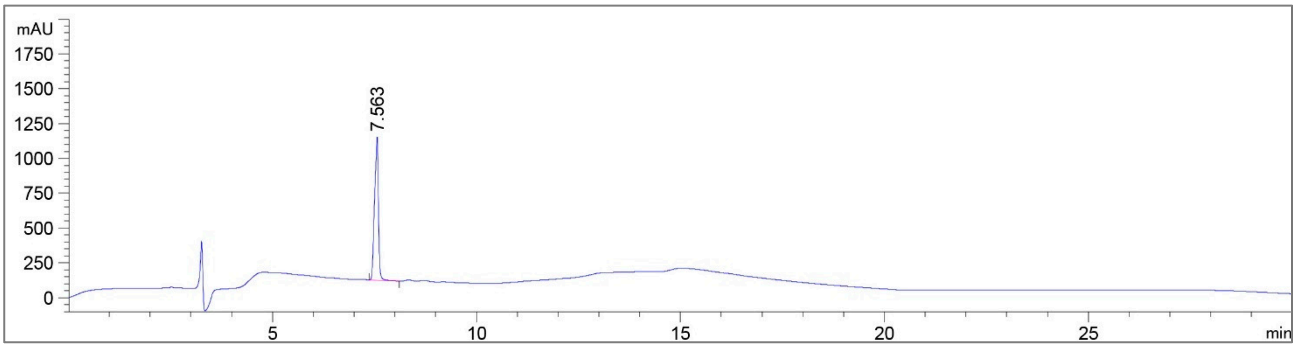

Figure S76. Chromatogram of *N*-Boc-Ser-OH.

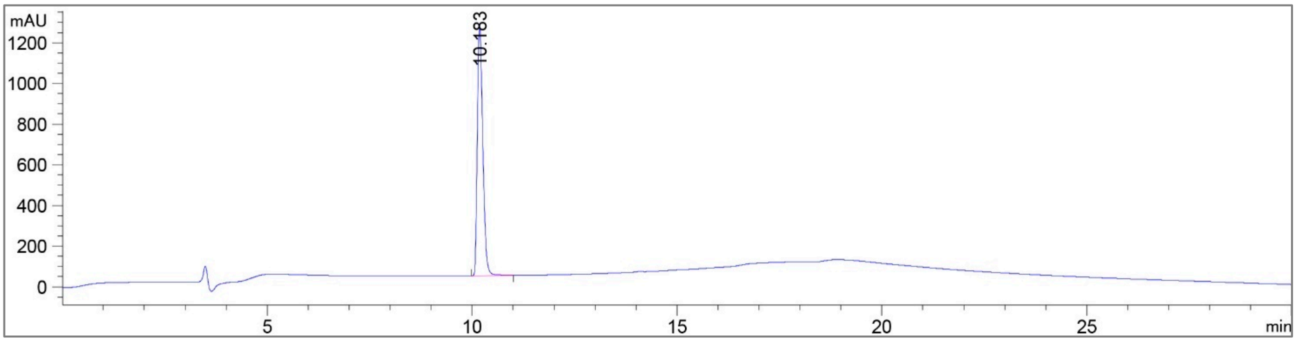

Figure S77. Chromatogram of *N*-Boc-Trp-OH.

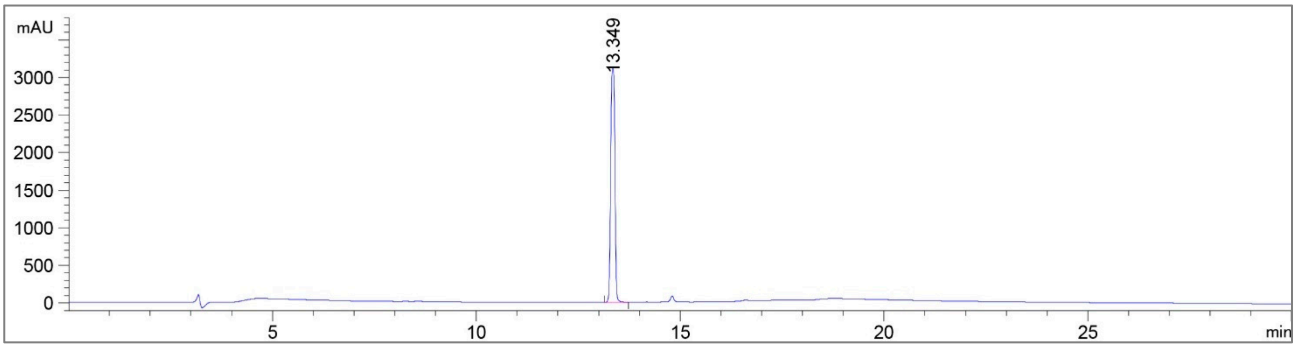

Figure S78. Chromatogram of *N*-Fmoc-Aib-OH.

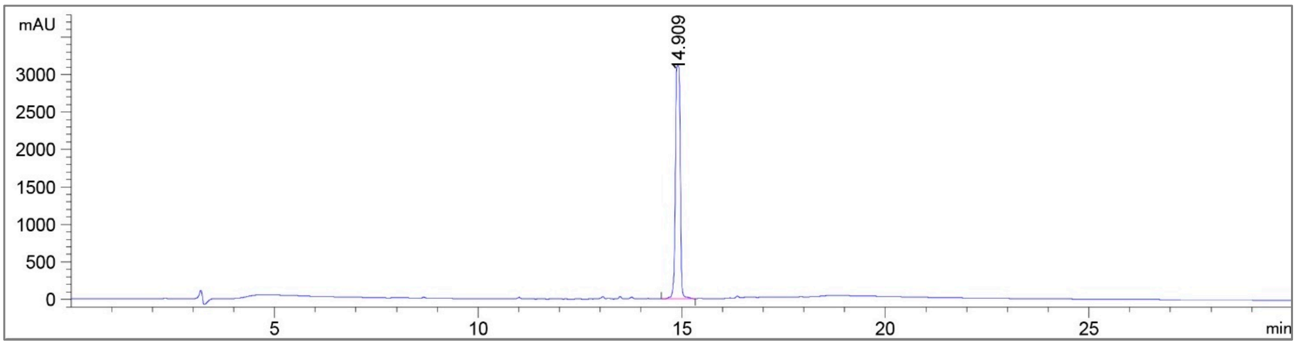

Figure S79. Chromatogram of *N*-Fmoc-Arg(PBF)-OH.

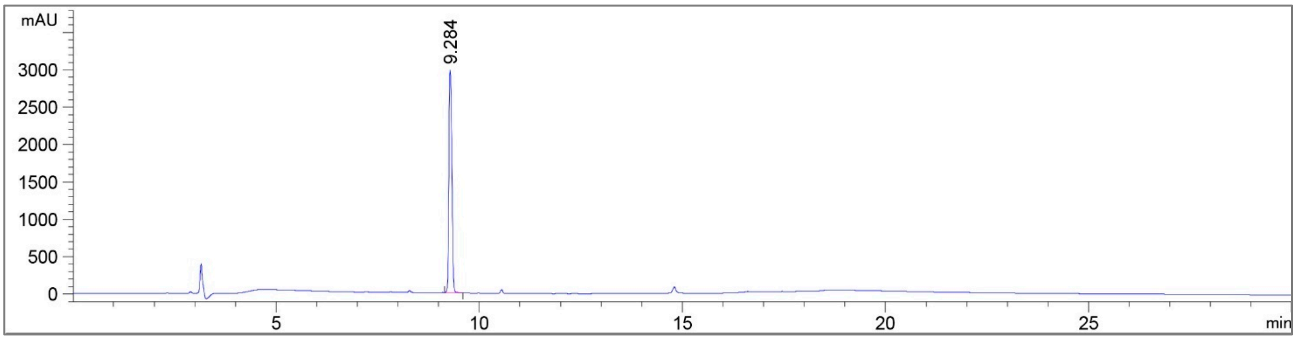

Figure S80. Chromatogram of *N*-Fmoc-Arg(HCl)-OH.

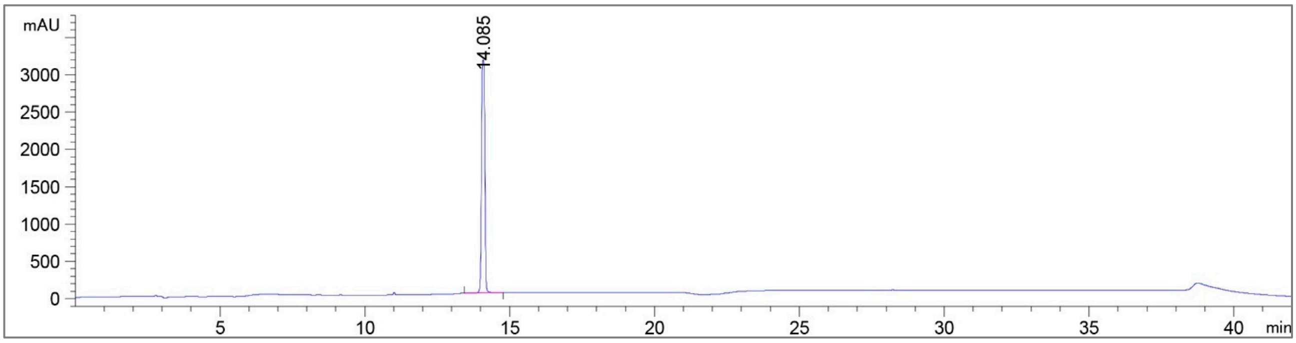

Figure S81. Chromatogram of *N*-Fmoc-Asp(tBu)-OH.

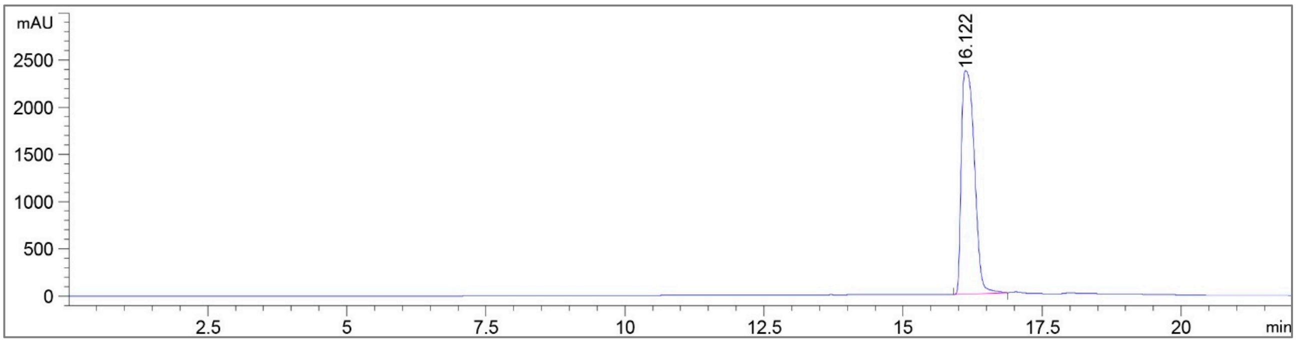

Figure S82. Chromatogram of *N*-Fmoc-Leu-OH.

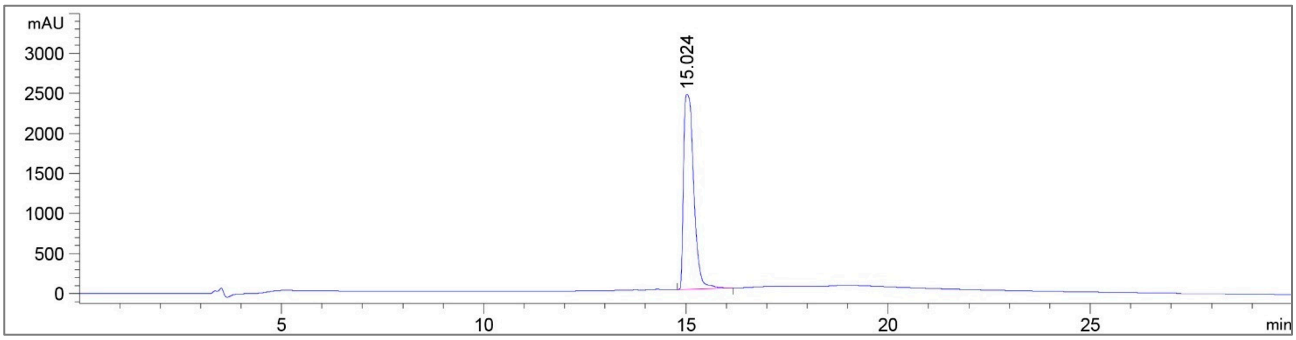

Figure S83. Chromatogram of *N*-Fmoc-Phe-OH.

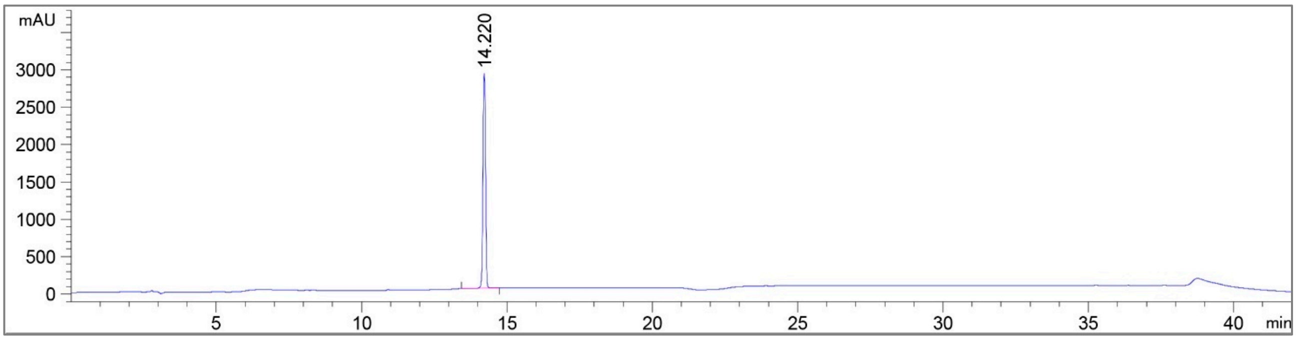

Figure S84. Chromatogram of *N*-Fmoc-Ser(tBu)-OH.

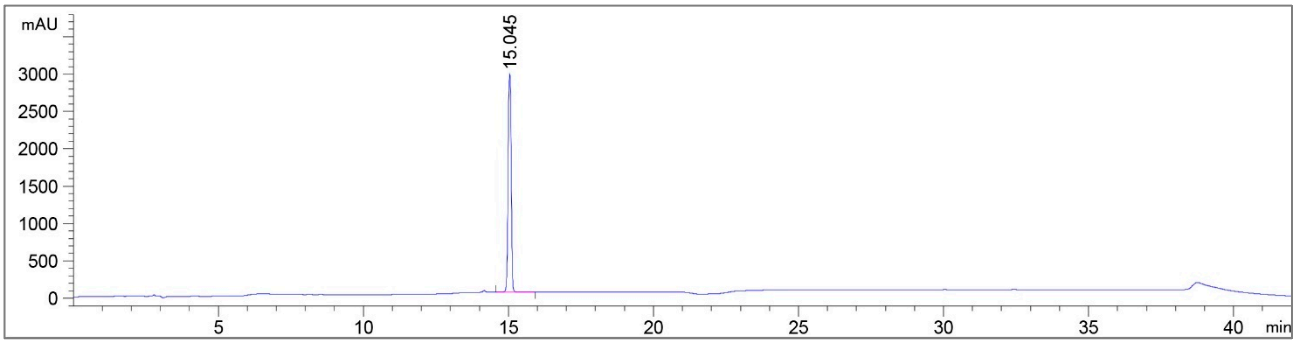

Figure S85. Chromatogram of *N*-Fmoc-Thr(tBu)-OH.

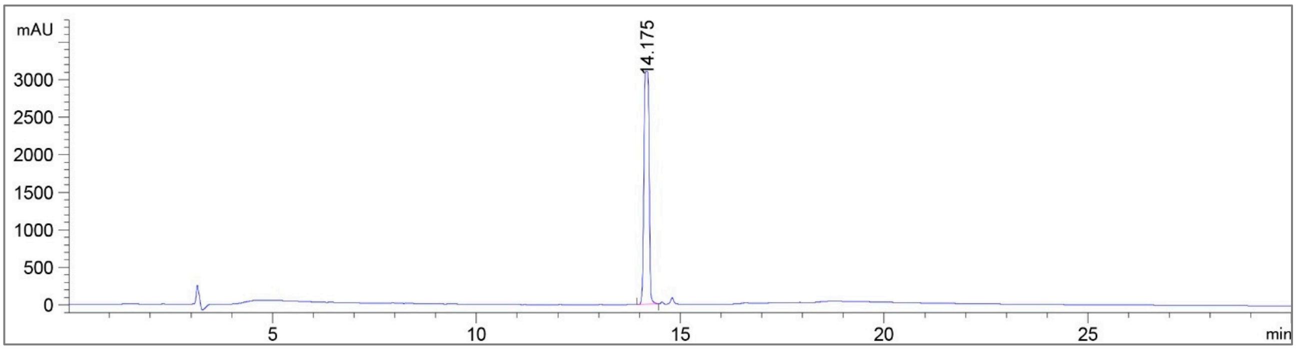

Figure S86. Chromatogram of *N*-Fmoc-Trp-OH.

8. NMR spectra

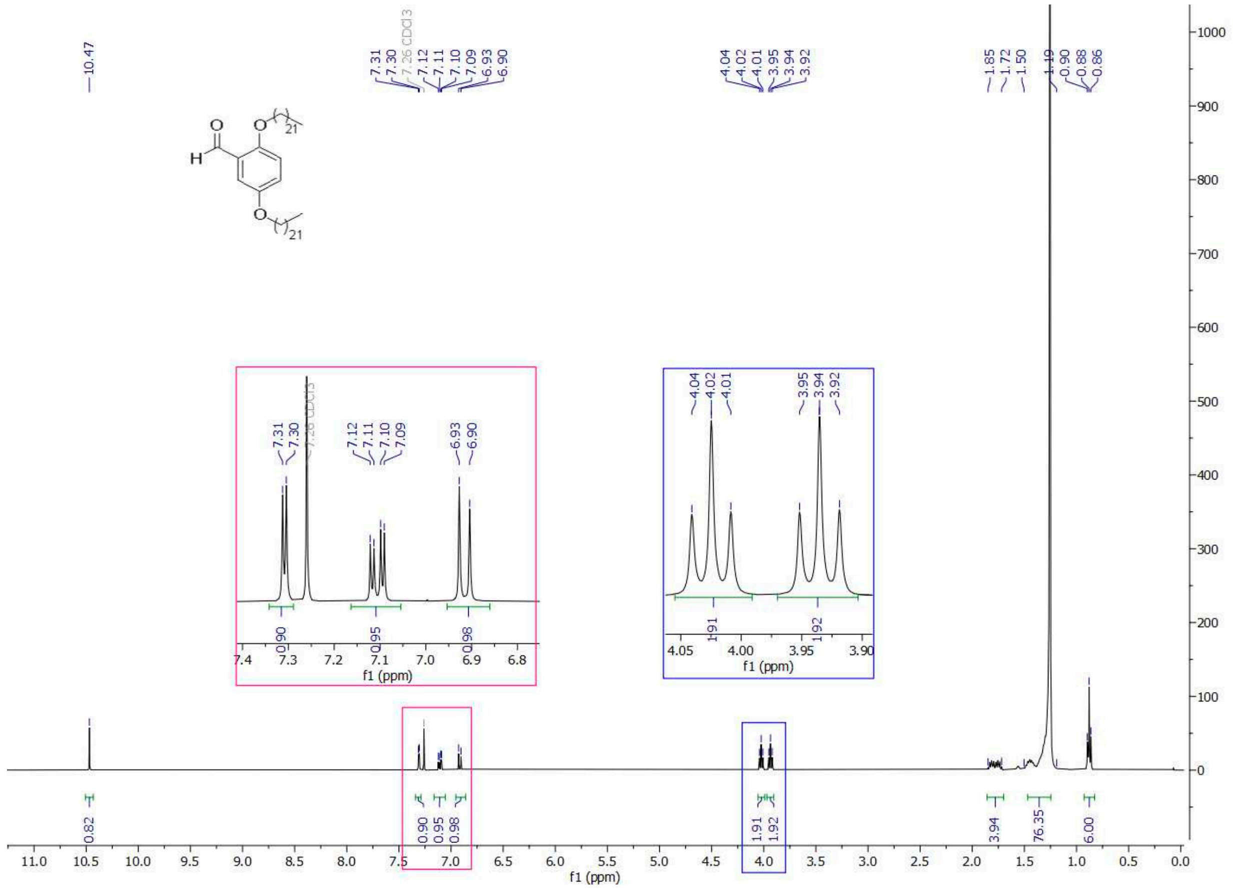

Figure S87. ^{1}H-NMR (400 MHz, $CDCl_3$) spectra of 2,5-di(dococyloxy)benzaldehyde.

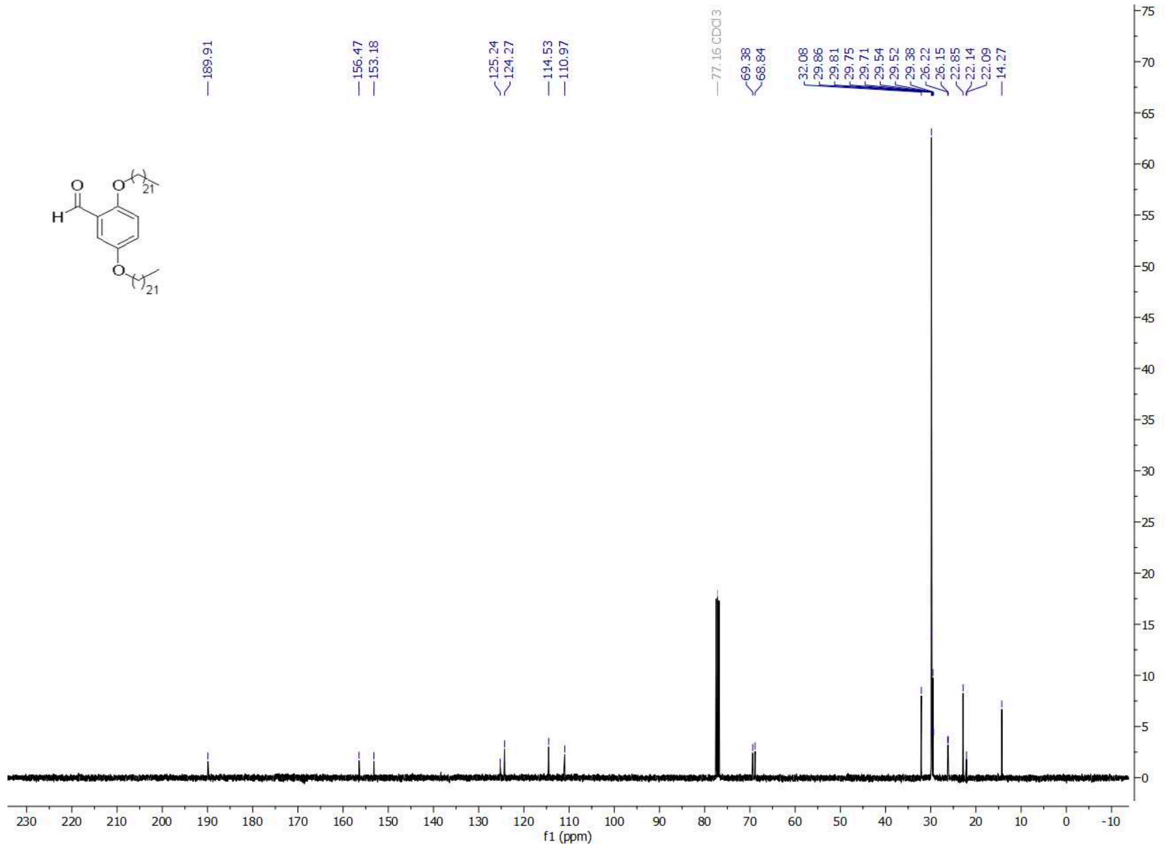

Figure S88. ^{13}C-NMR (100 MHz, $CDCl_3$) spectra of 2,5-di(dococyloxy)benzaldehyde.

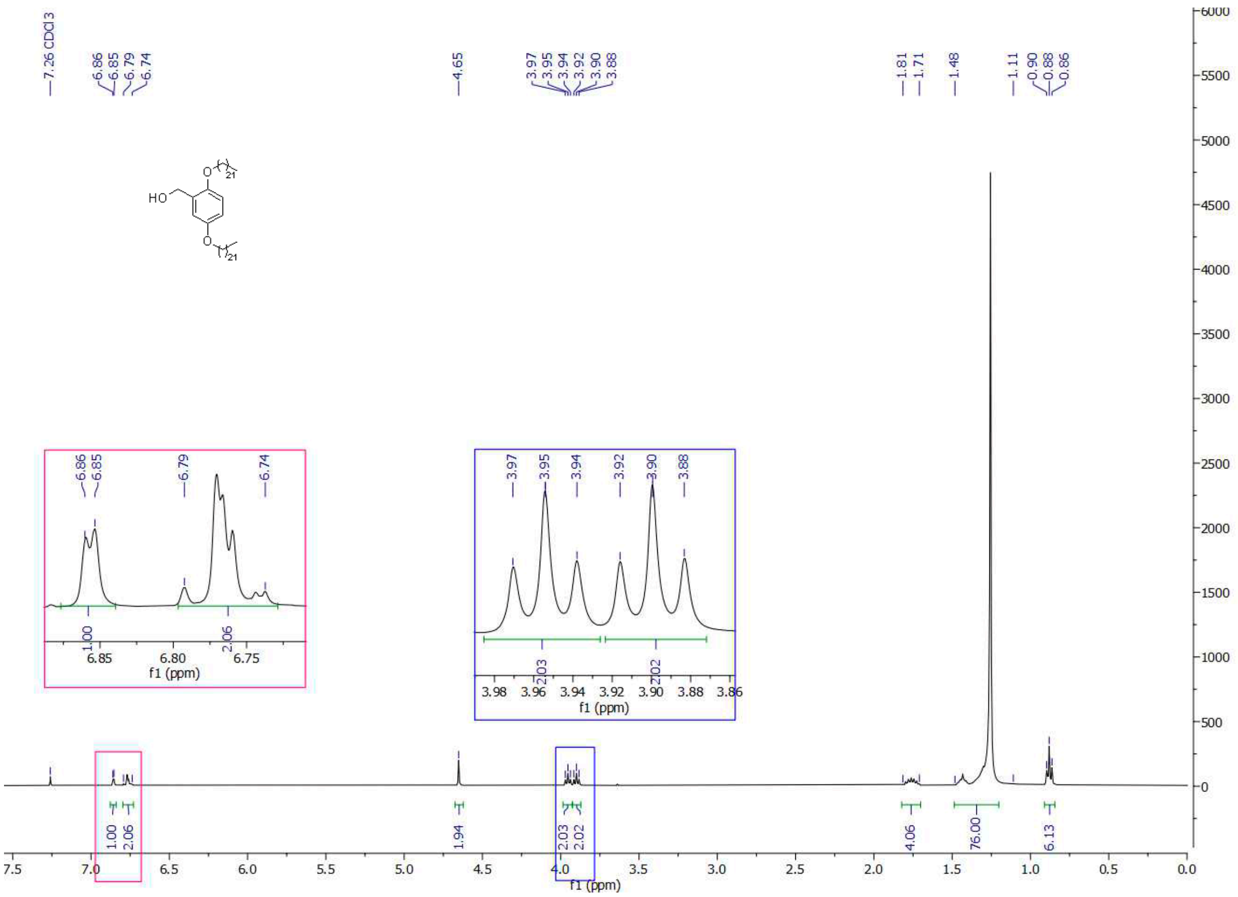

Figure S89. ^{1}H-NMR (400 MHz, $CDCl_3$) spectra of (2,5-bis(docosyloxy)phenyl)methanol.

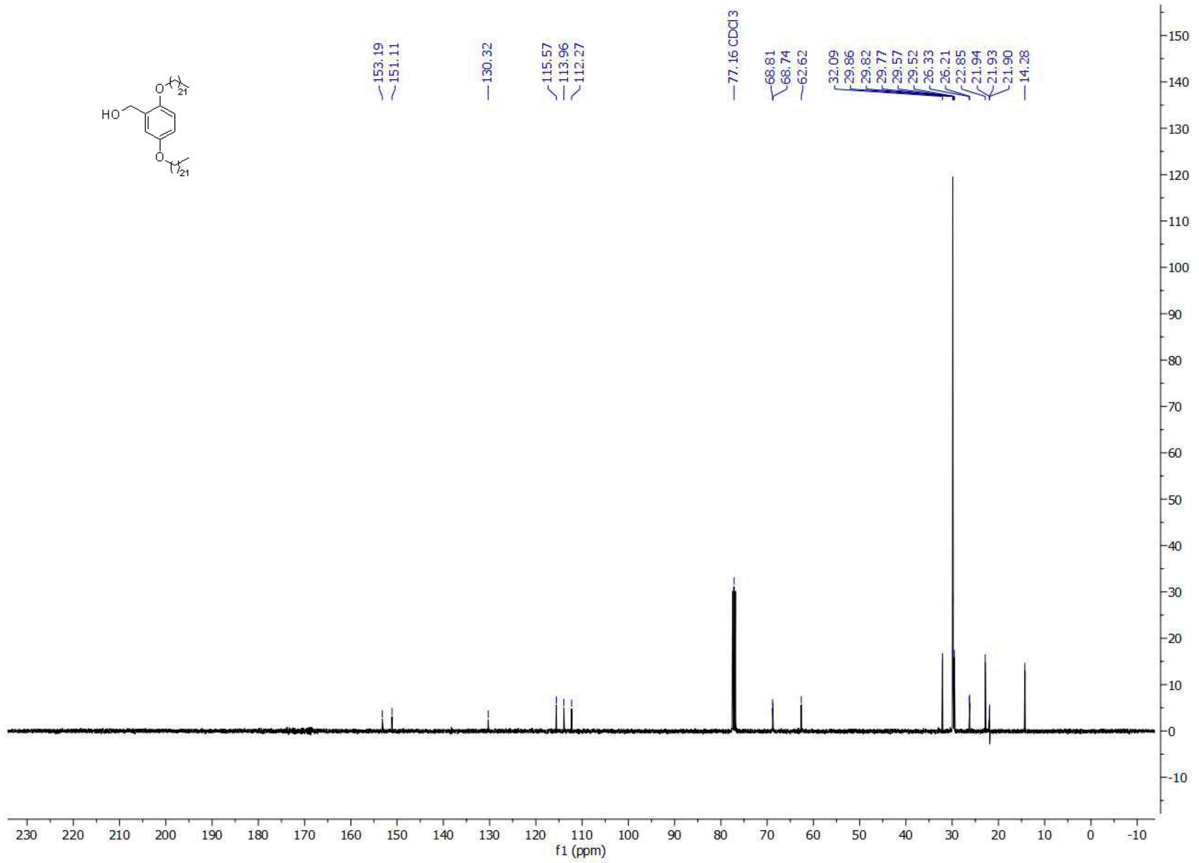

Figure S90. ^{13}C-NMR (100 MHz, $CDCl_3$) spectra of (2,5-bis(docosyloxy)phenyl)methanol.

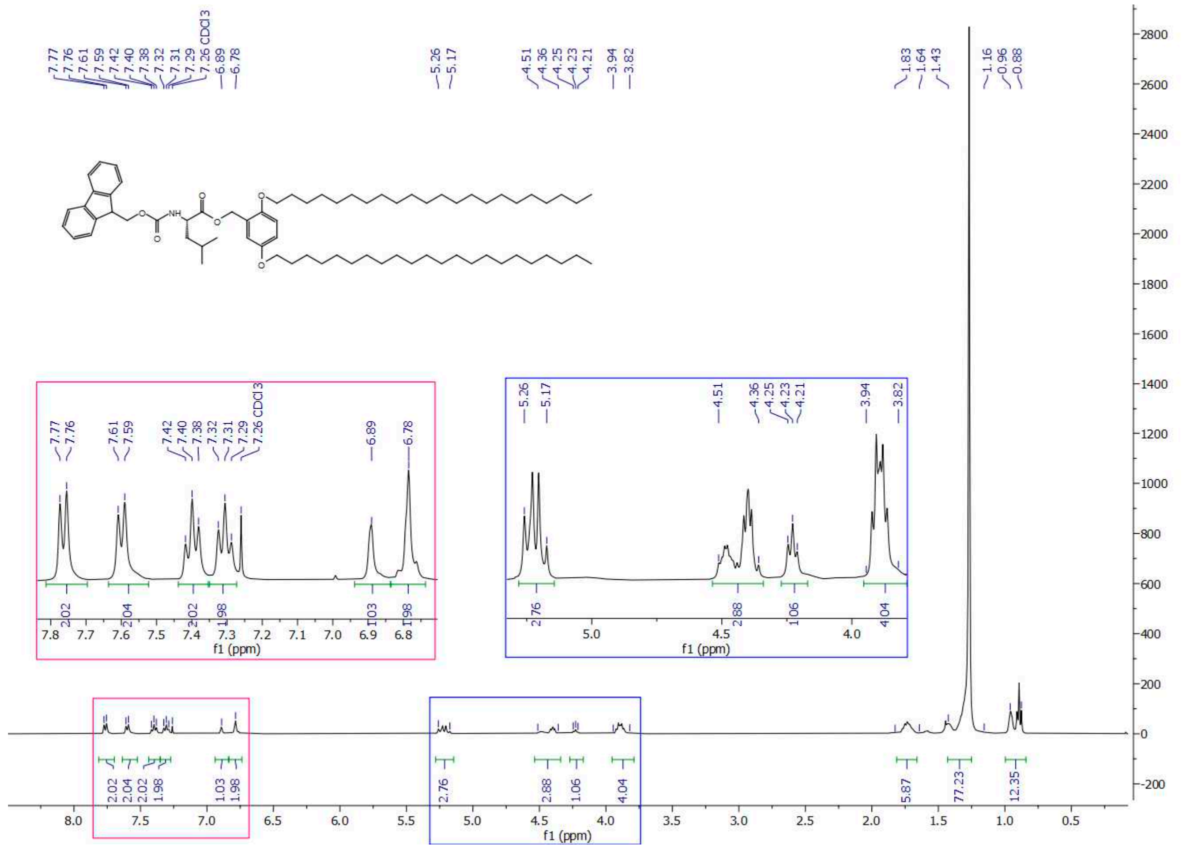

Figure S91. ^{1}H-NMR (400 MHz, $CDCl_3$) spectra of *N*-Fmoc-Leu-Tag.

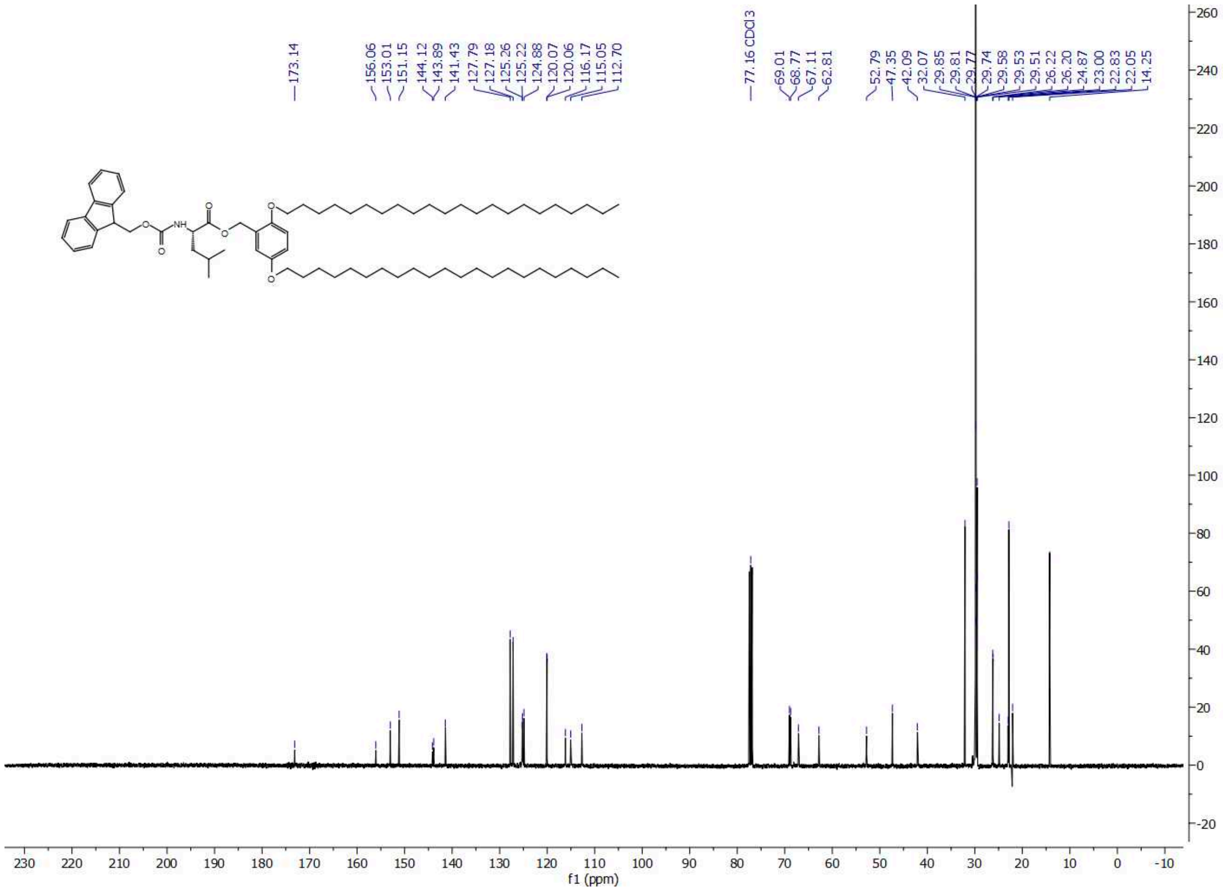

Figure S92. ^{13}C-NMR (100 MHz, $CDCl_3$) spectra of *N*-Fmoc-Leu-Tag.

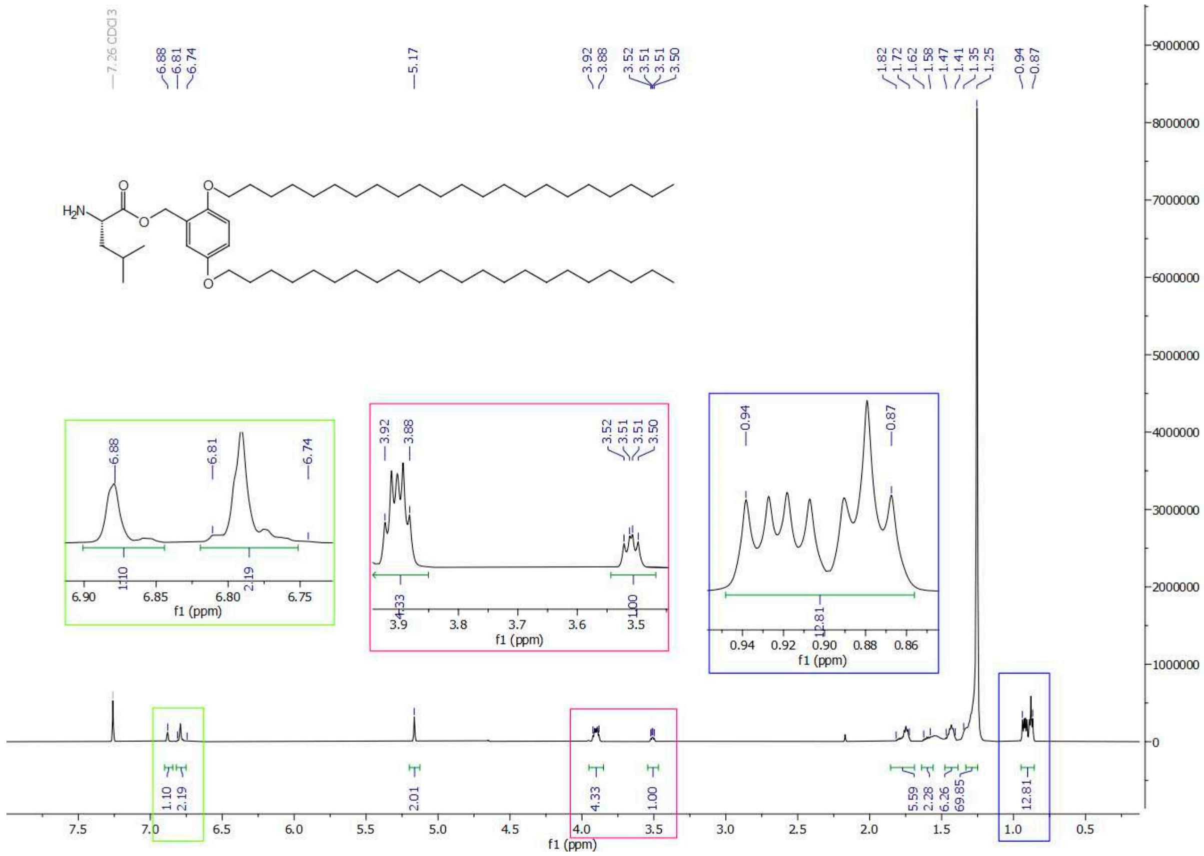

Figure S93. ^{1}H-NMR (600 MHz, $CDCl_3$) spectra of H_2N-Leu-Tag.

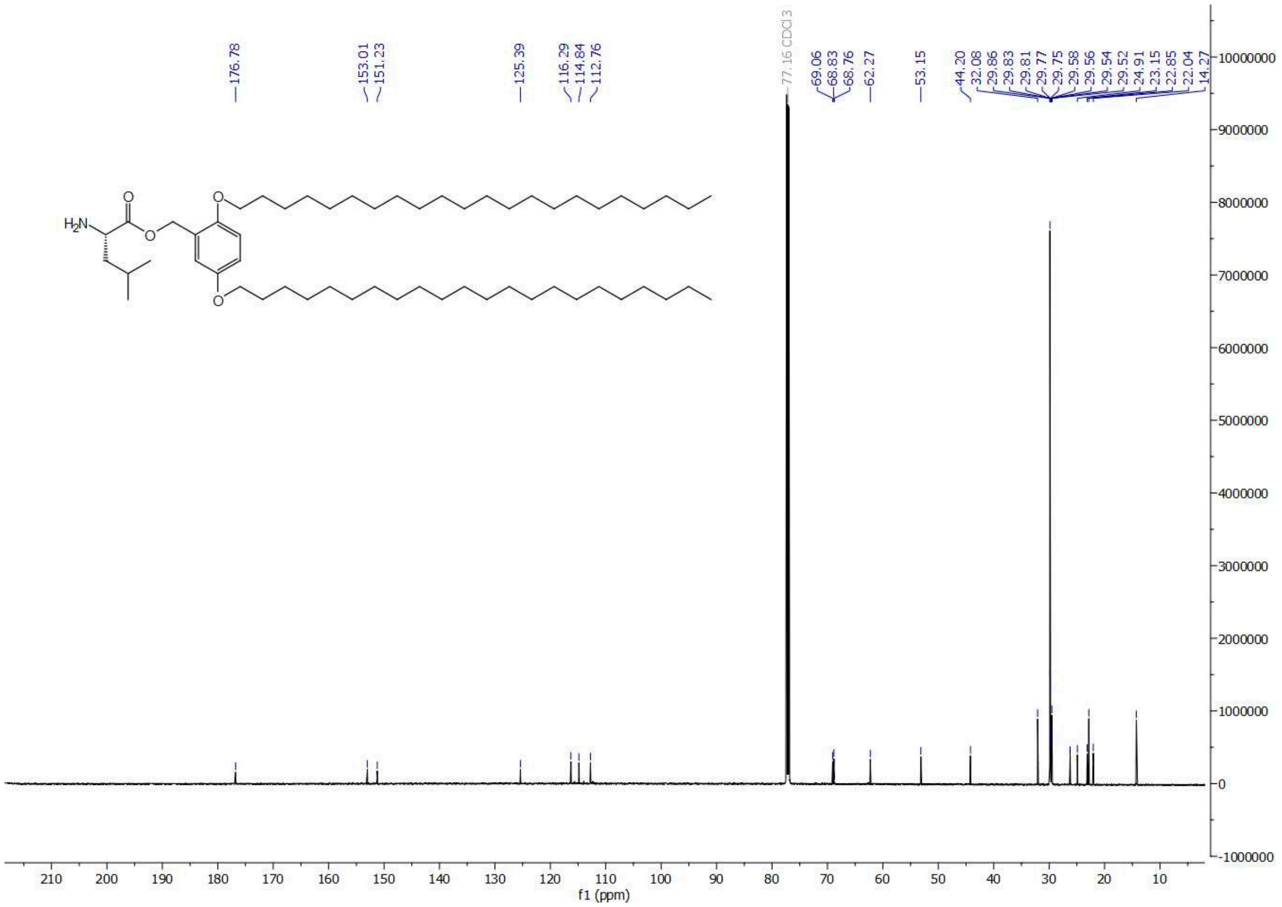

Figure S94. ^{13}C-NMR (150 MHz, $CDCl_3$) spectra of H_2N-Leu-Tag.

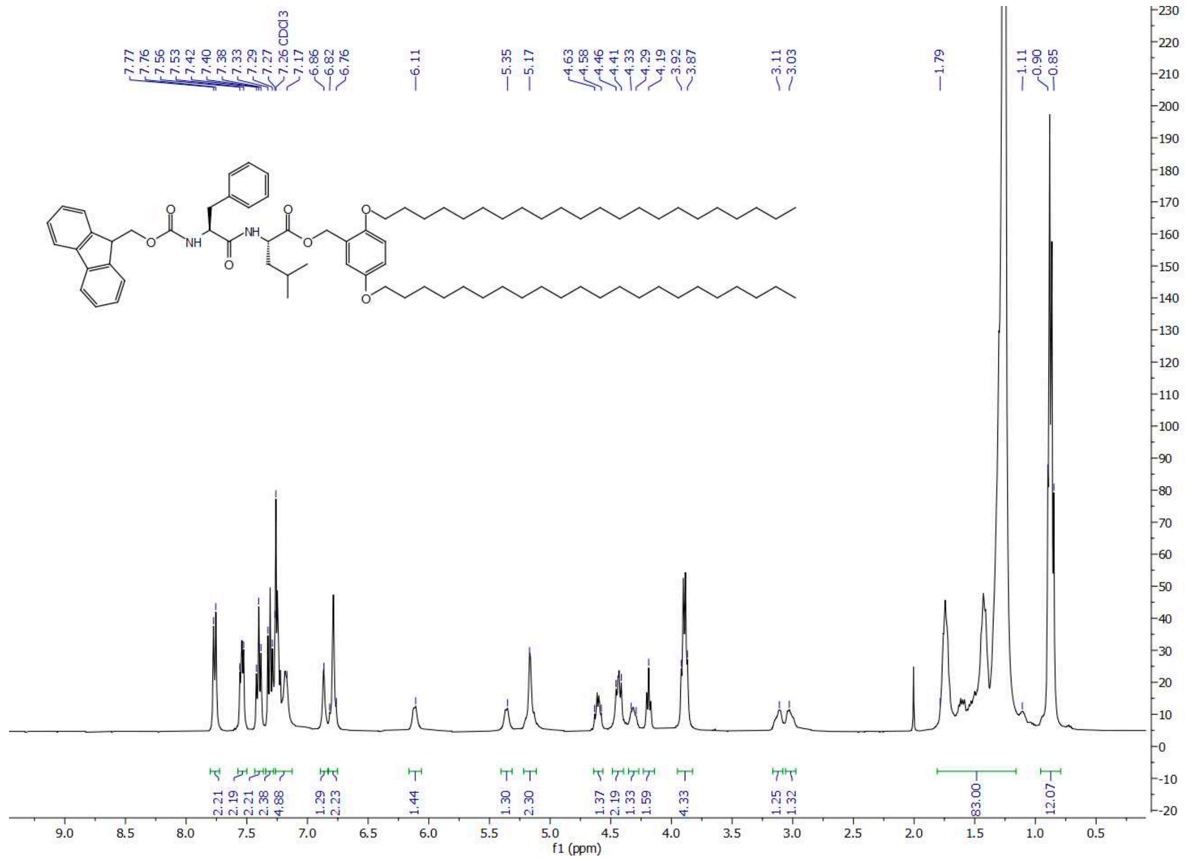

Figure S95. ^{1}H-NMR (400 MHz, $CDCl_3$) spectra of *N*-Fmoc-Phe-Leu-Tag.

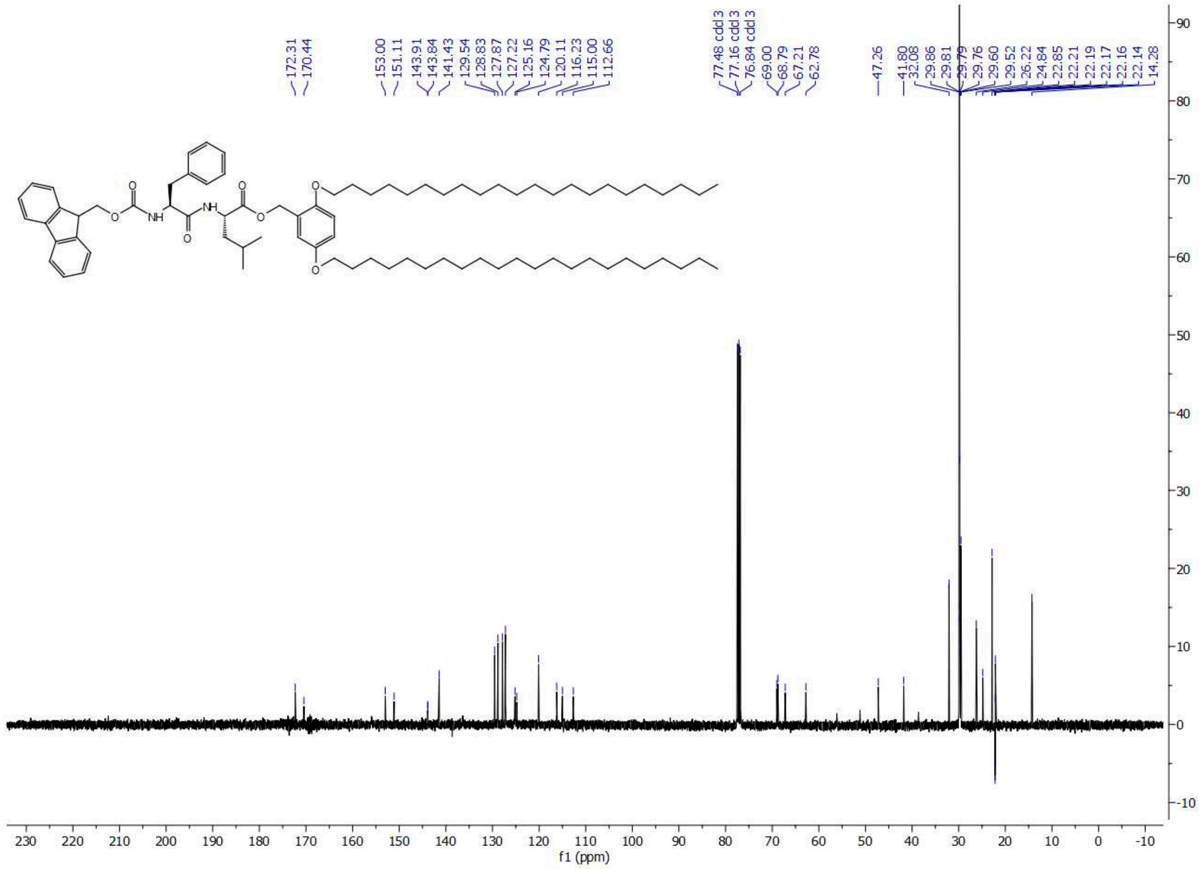

Figure S96. ^{13}C-NMR (100 MHz, $CDCl_3$) spectra of *N*-Fmoc-Phe-Leu-Tag.

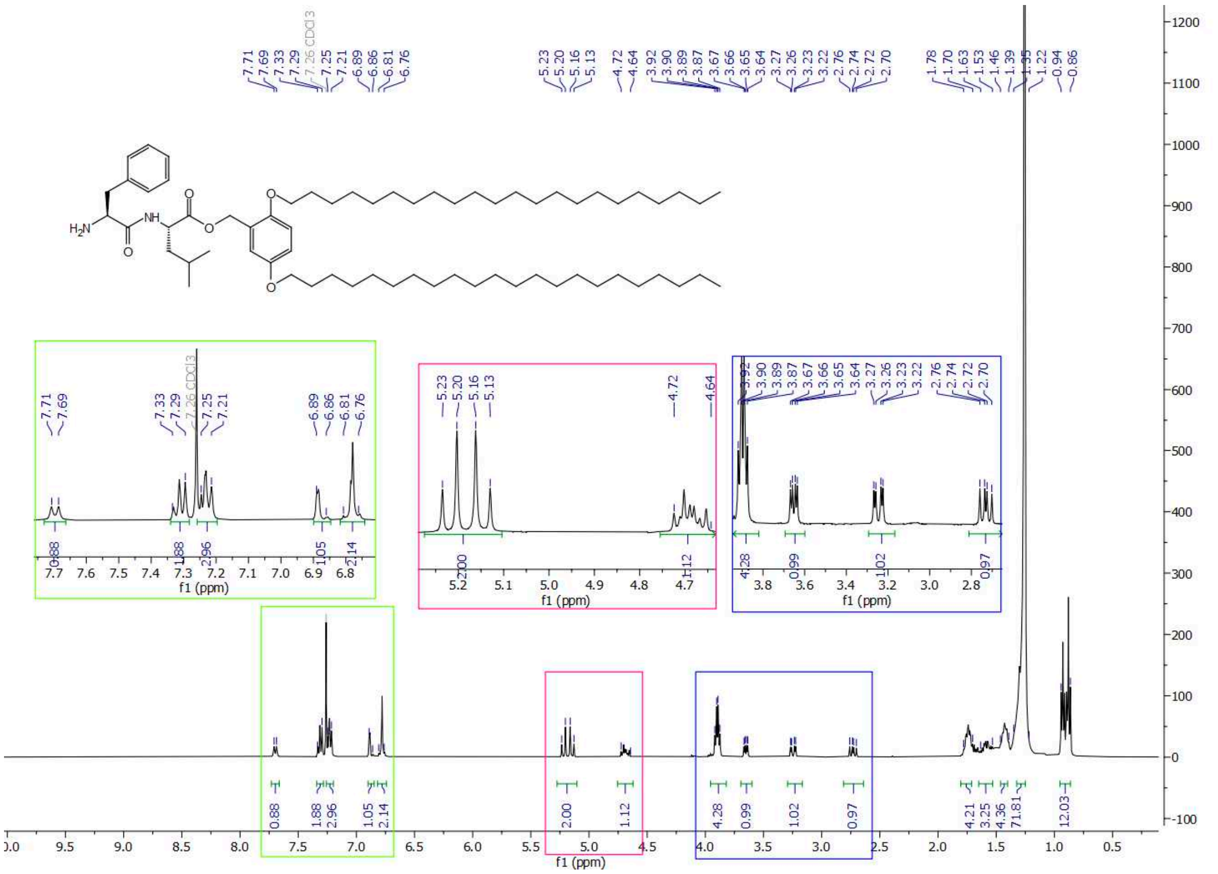

Figure S97. ^{1}H-NMR (600 MHz, $CDCl_3$) spectra of H_2N-Phe-Leu-Tag.

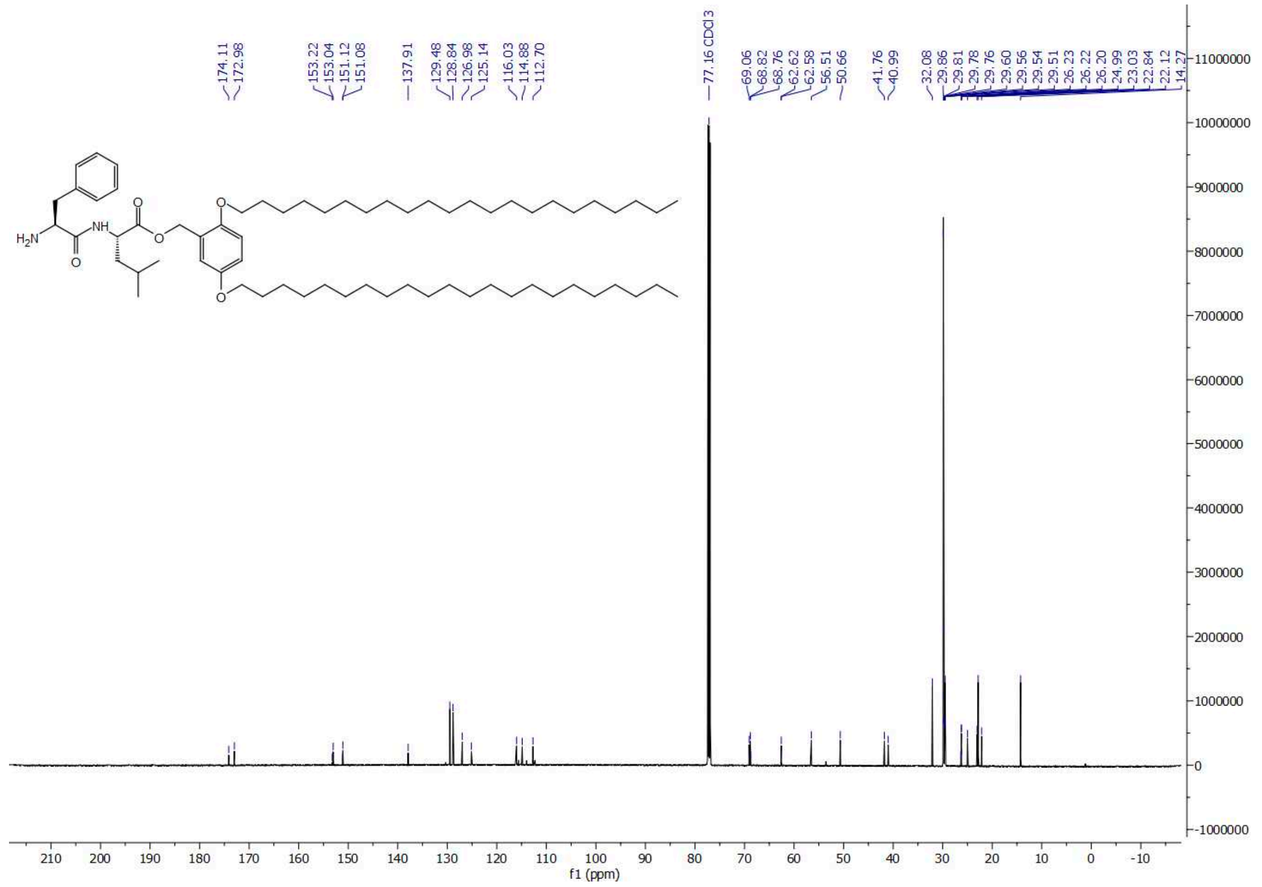

Figure S98. ^{13}C-NMR (150 MHz, $CDCl_3$) spectra of H_2N-Phe-Leu-Tag.

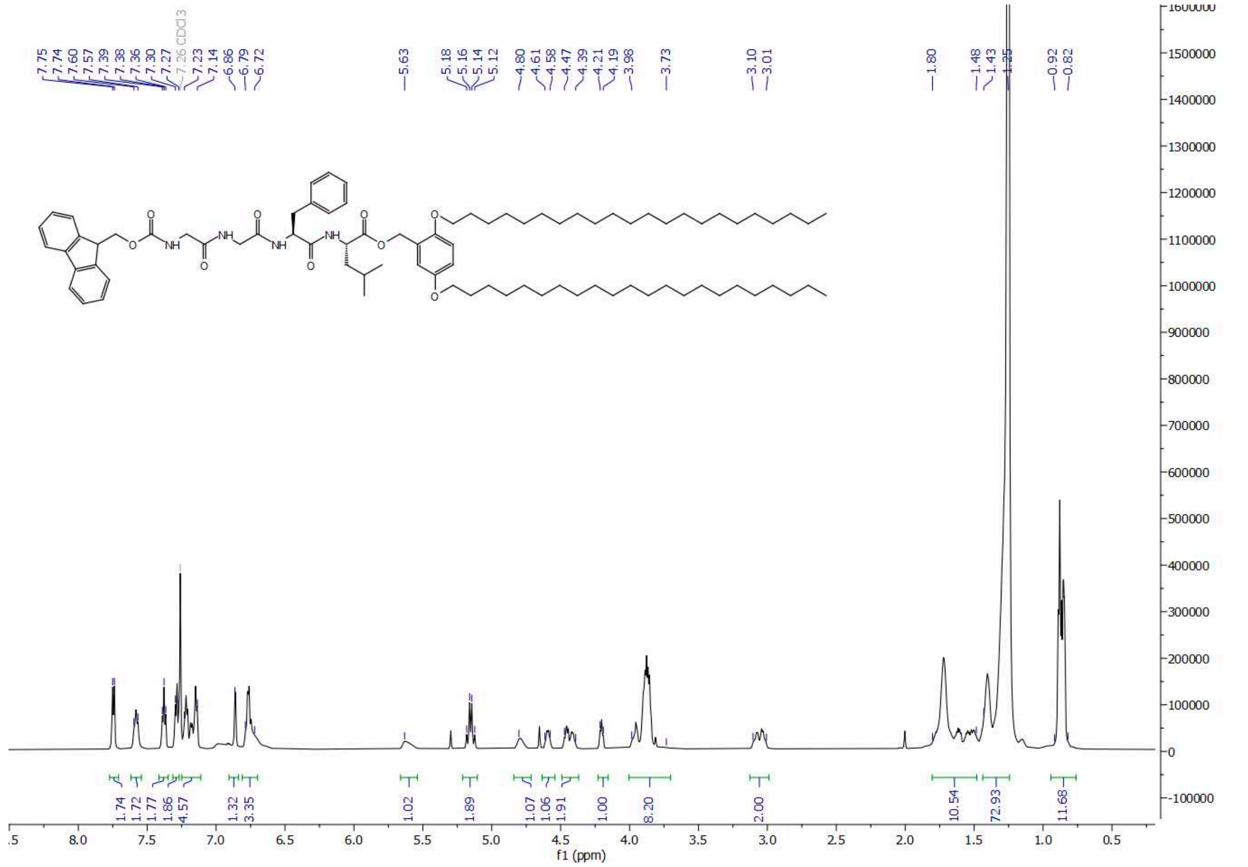

Figure S99. ^{1}H-NMR (600 MHz, $CDCl_3$) spectra of *N*-Fmoc-Gly-Gly-Phe-Leu-Tag.

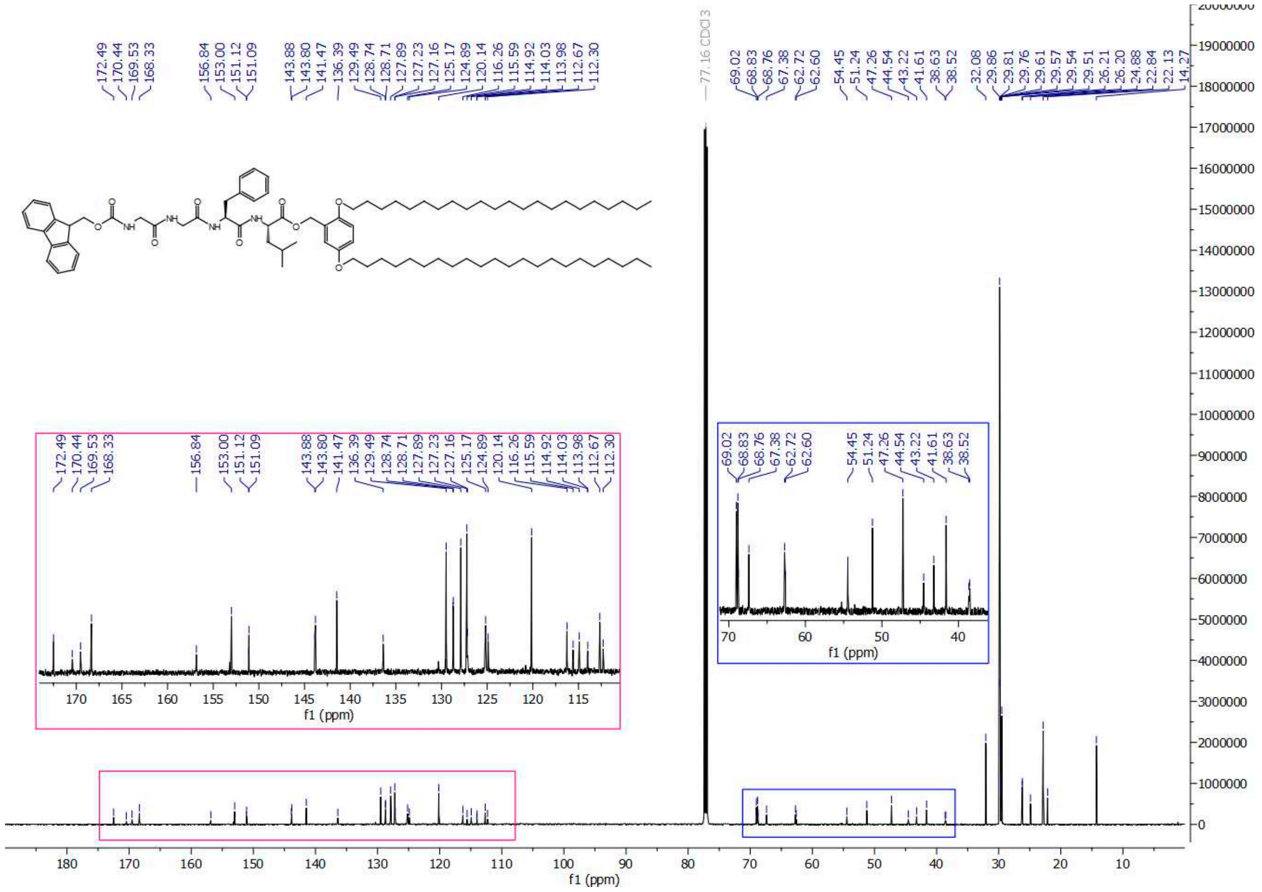

Figure S100. ^{13}C-NMR (150 MHz, $CDCl_3$) spectra of *N*-Fmoc-Gly-Gly-Phe-Leu-Tag.

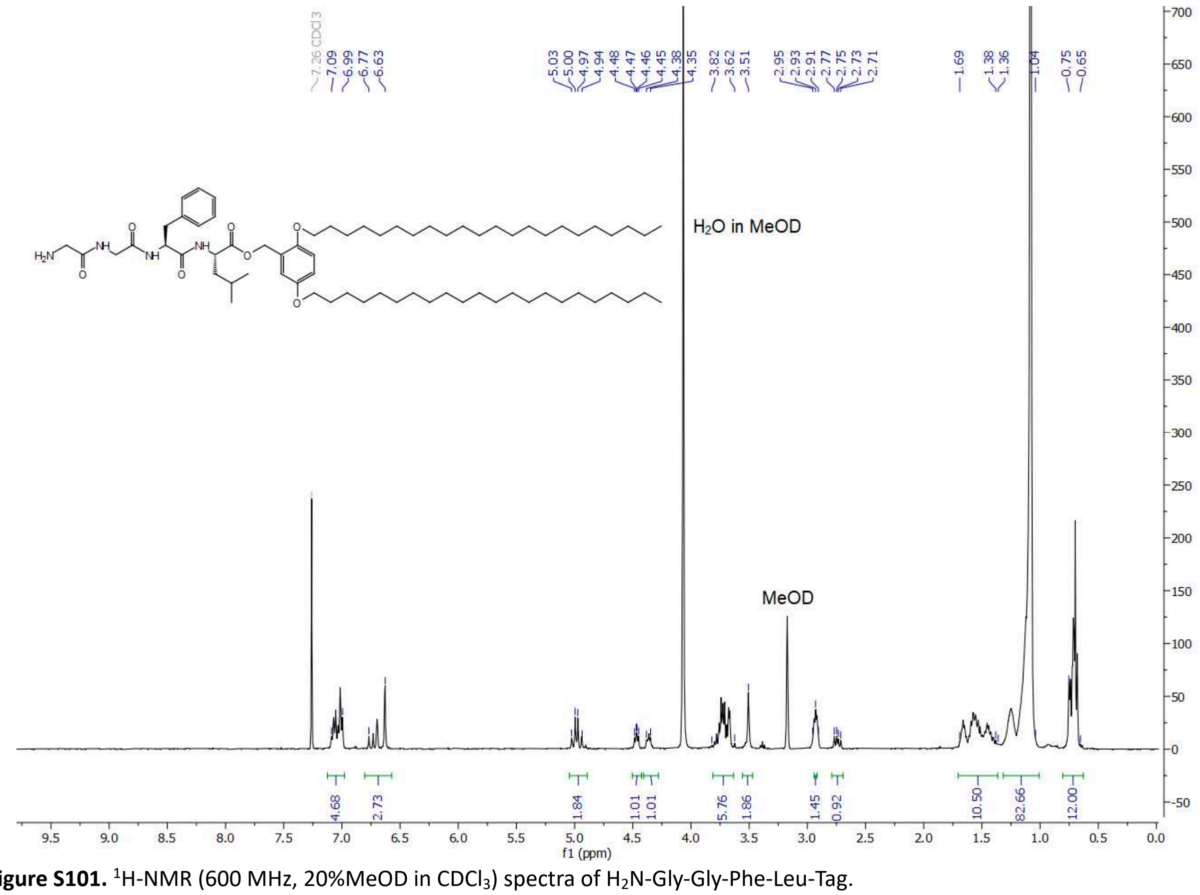

Figure S101. ^{1}H-NMR (600 MHz, 20%MeOD in $CDCl_3$) spectra of H_2N-Gly-Gly-Phe-Leu-Tag.

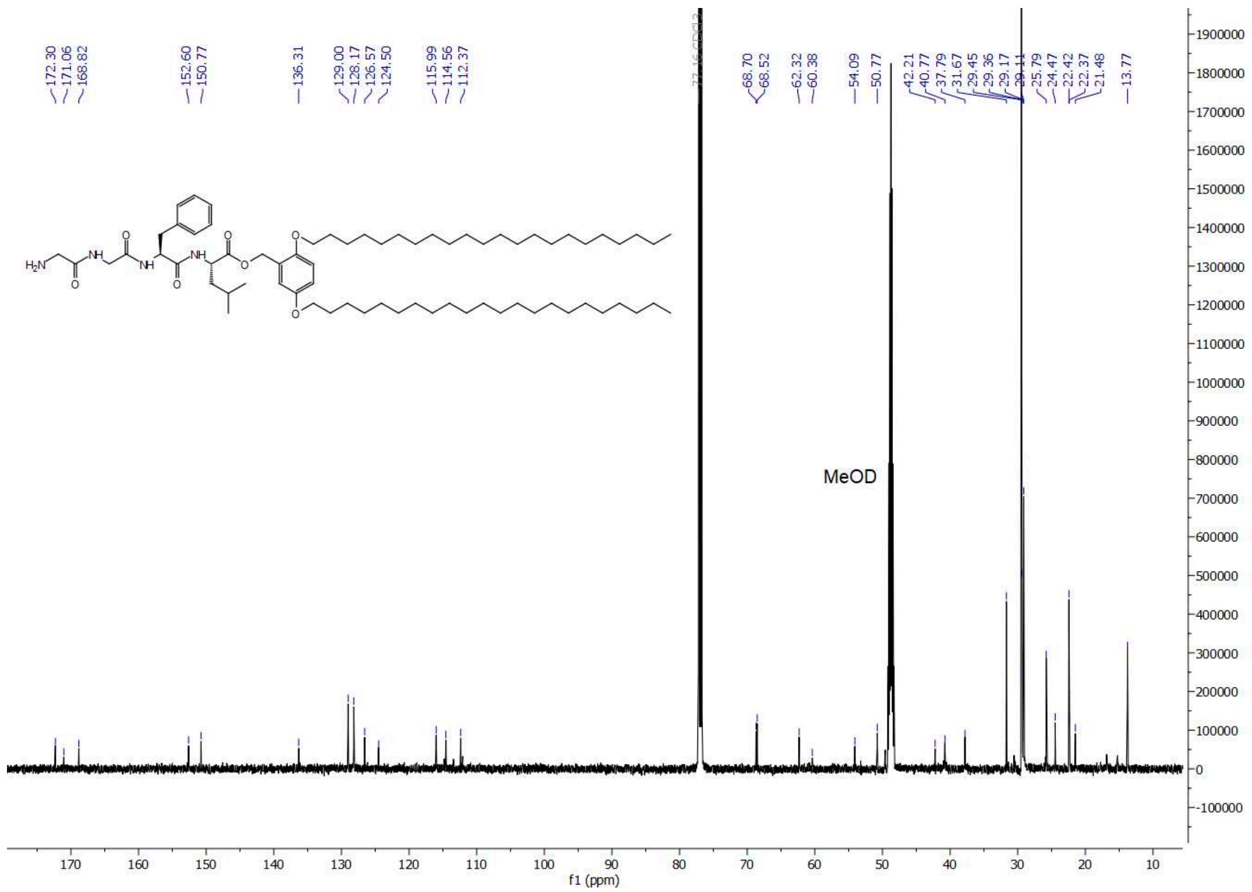

Figure S102. ^{13}C-NMR (150 MHz, 20%MeOD in $CDCl_3$) spectra of H_2N-Gly-Gly-Phe-Leu-Tag.

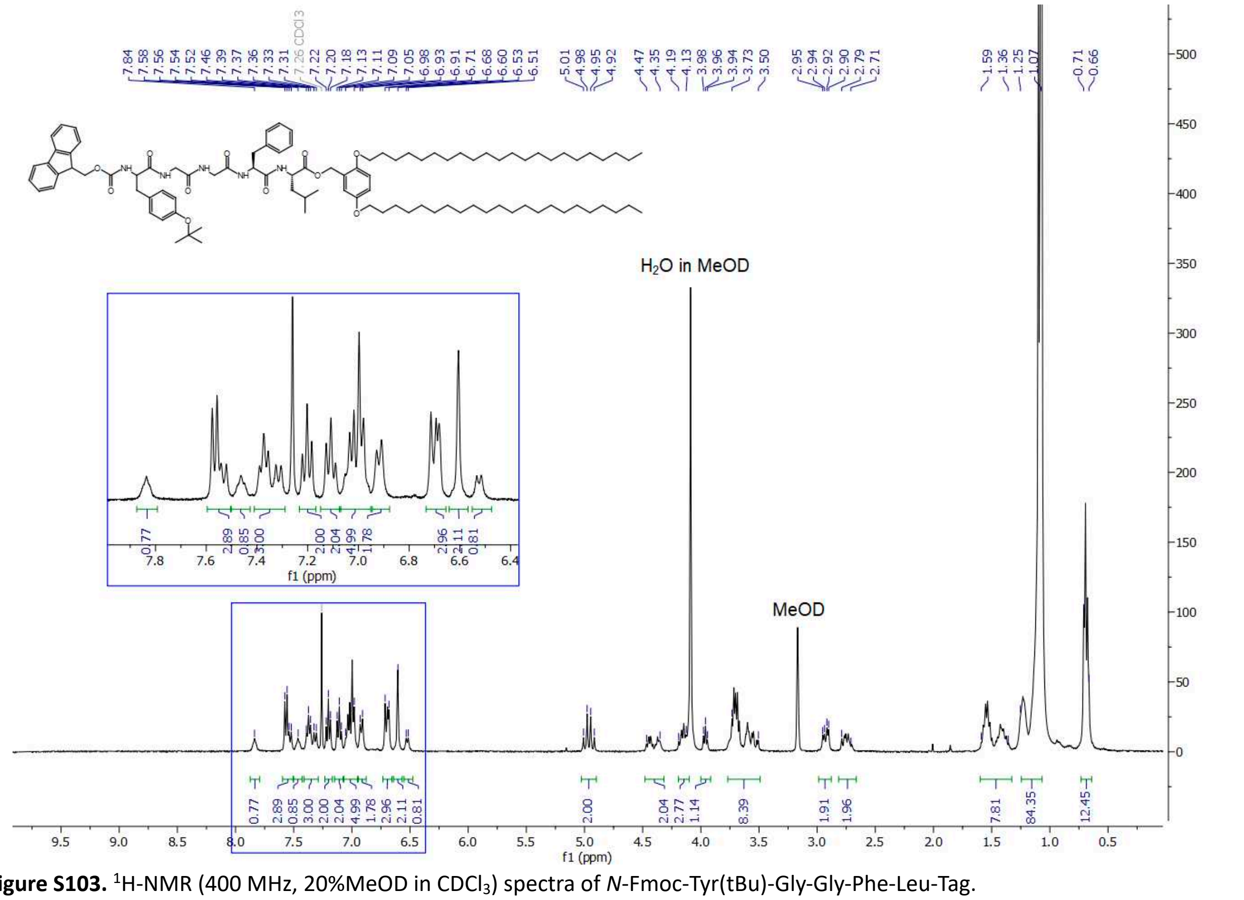

Figure S103. ^{1}H-NMR (400 MHz, 20%MeOD in $CDCl_3$) spectra of *N*-Fmoc-Tyr(tBu)-Gly-Gly-Phe-Leu-Tag.

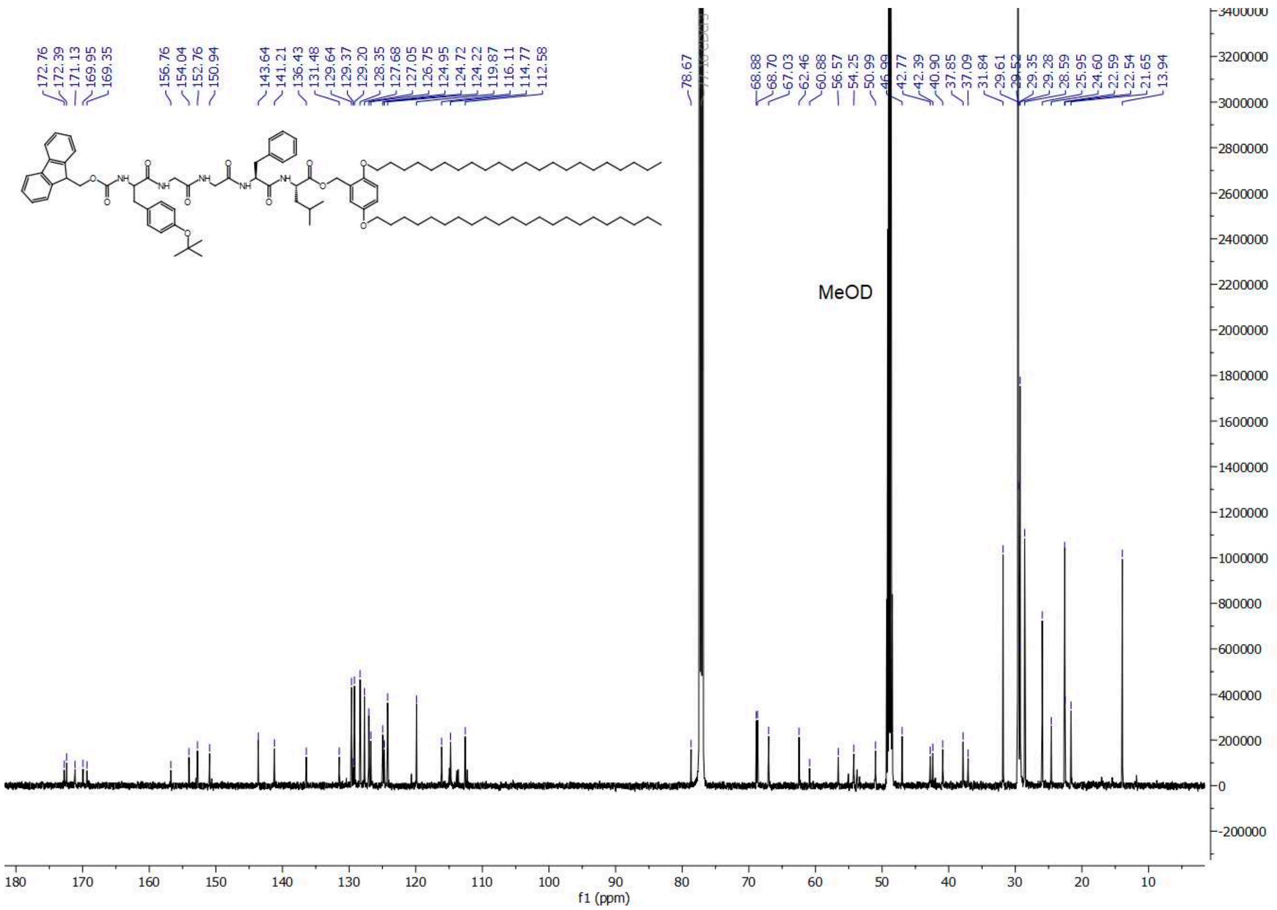

Figure S104. ^{13}C-NMR (150 MHz, 20%MeOD in $CDCl_3$) spectra of *N*-Fmoc-Tyr(tBu)-Gly-Gly-Phe-Leu-Tag.

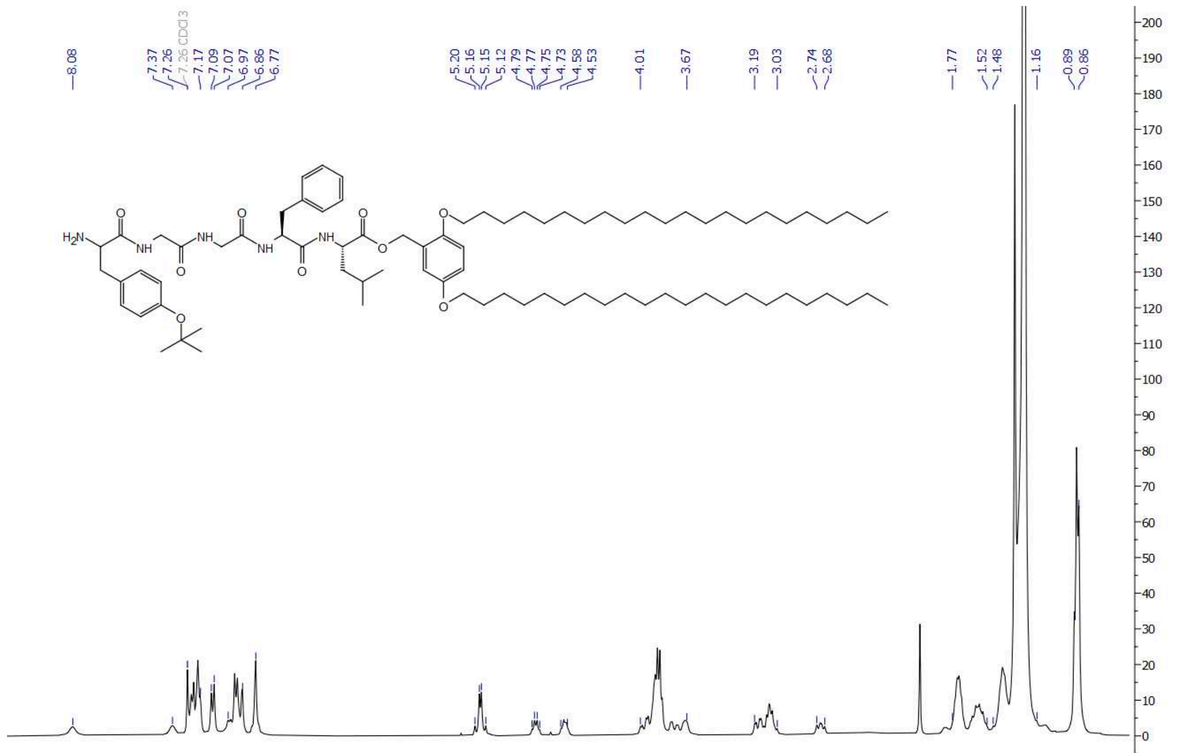

Figure S105. ^{1}H-NMR (400 MHz, $CDCl_3$) spectra of H_2N-Tyr(tBu)-Gly-Gly-Phe-Leu-Tag.

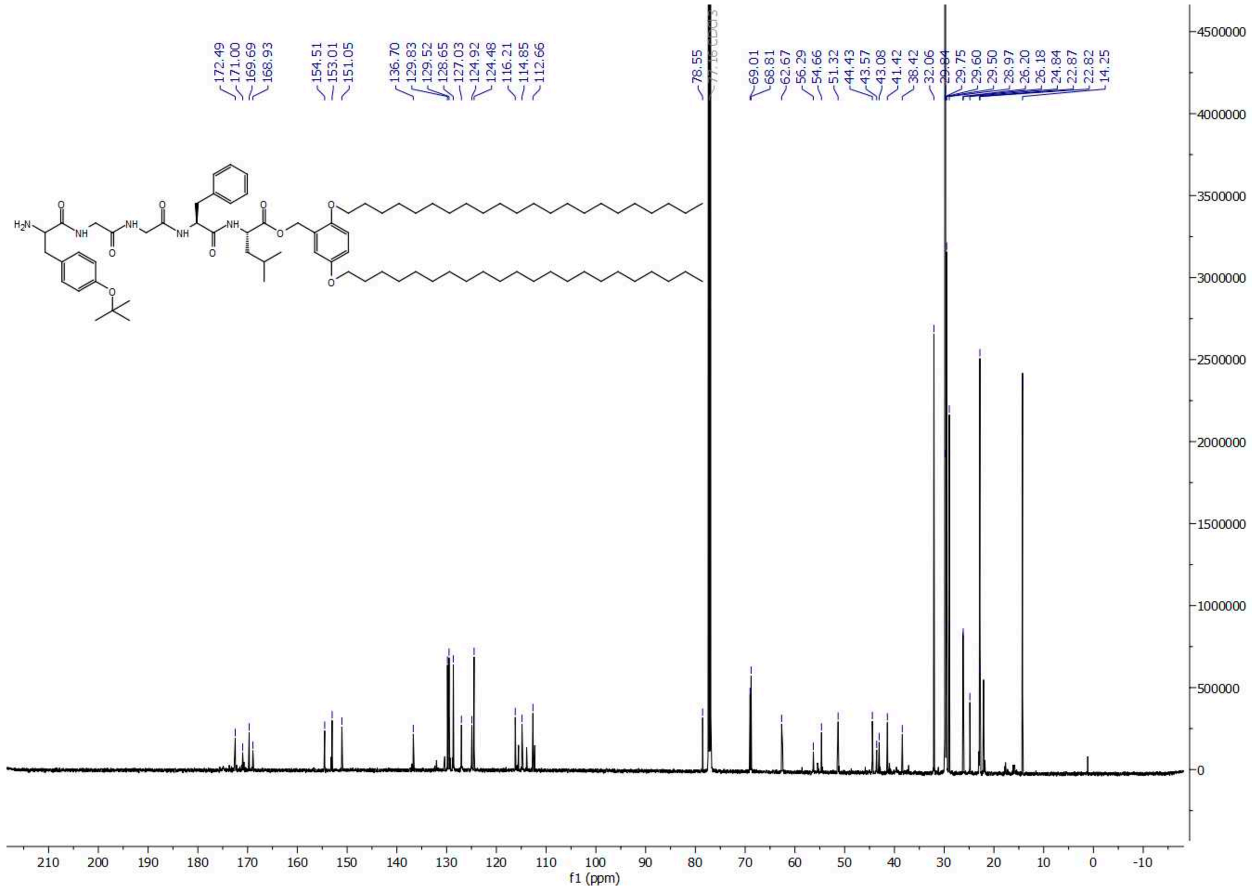

Figure S106. ^{13}C-NMR (150 MHz, $CDCl_3$) spectra of H_2N-Tyr(tBu)-Gly-Gly-Phe-Leu-Tag.
